# *N*-Alkyl-2-Quinolonopyrones Demonstrate Antimicrobial Activity against ESKAPE Pathogens Including *Staphylococcus aureus*

**DOI:** 10.1021/acsmedchemlett.2c00185

**Published:** 2022-07-19

**Authors:** Eoin Moynihan, Katrina Mackey, Mark A. T. Blaskovich, F. Jerry Reen, Gerard McGlacken

**Affiliations:** † School of Chemistry and Analytical and Biological Chemistry Research Facility, University College Cork, Cork T12 YN60, Ireland; ‡ Community for Open Antimicrobial Drug Discovery, Centre for Superbug Solutions, Institute for Molecular Bioscience, The University of Queensland, St. Lucia, Queensland 4072, Australia; § School of Microbiology, University College Cork, Cork T12 K8AF, Ireland

**Keywords:** Antibiotic, MRSA, 2-pyrone, SAR

## Abstract

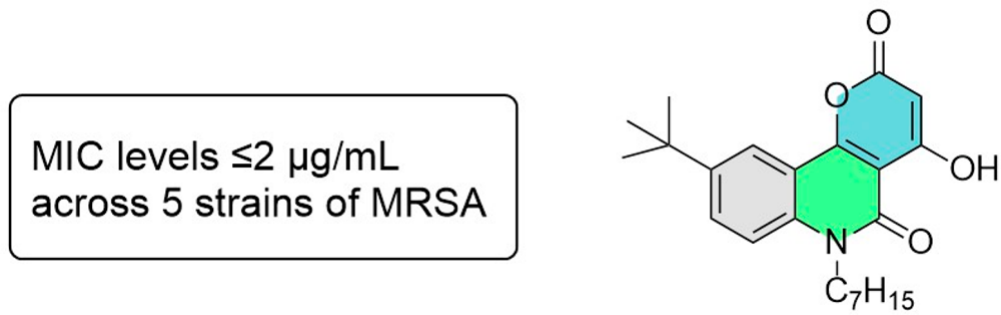

Antibiotic resistance has grown significantly in the last three decades, while research and development of new antibiotic classes has languished. Therefore, new chemical frameworks for the control of microbial behavior are urgently required. This study presents a novel suite of compounds, based on a tricyclic 4-hydroxy-2*H*-pyrano[3,2-*c*]quinoline-2,5(6*H*)-dione core, with significant antibiotic activity against the ESKAPE pathogens *Staphylococcus aureus* and *Enterococcus faecalis* and the “accidental pathogen” *Staphylococcus epidermidis*. A potent analogue with an *N*-heptyl-9-*t-*Bu substitution pattern emerged as a hit with MIC levels ≤2 μg/mL across four strains of MRSA. In addition, the same compound proved highly potent against *Enterococcus* spp. (0.25 μg/mL).

Antimicrobial resistance (AMR) poses a significant challenge to society, one that if unmet, will result in significant mortality from infections that are currently manageable in the clinic. The “perfect storm” of increased resistance within populations of key opportunistic pathogens (such as the ESKAPE group: *Enterococcus* sp., *Staphylococcus aureus*, *Klebsiella pneumoniae*, *Acinetobacter baumanii*, *Pseudomonas aeruginosa*, and *Enterobacter* sp.) and a decline in the “discovery” of new antibiotic classes is of serious concern.^1^ Apart from antibiotic stewardship, the key to addressing this challenge remains the development of new and effective antimicrobials, and yet, we are approaching three decades of what has been described as the discovery void.^2,3^ New antibiotics brought to market over the last 30 years have typically been modifications of existing antibiotic classes, whereby mechanisms of resistance tend to be selected for within a very short period of time.

4-Quinolones are well-established as broad spectrum antibiotics.^4^ In addition, 4-quinolones (in particular, 2-alkyl-4-(1*H*)-quinolones (AHQs)) have been identified as “signaling” molecules in a number of bacterial species.^5^ Signaling enables microbes to communicate effectively at the population level, both within species (intraspecies) and across the species/kingdom divide (interspecies/interkingdom).^6^ The Pseudomonas quinolone signal (PQS) and its des-hydroxy precursor (HHQ) are particularly well-established as AHQ signals in *Pseudomonas aeruginosa*.^7^ There is also some literature precedent that describes the antibacterial and antifungal activity of HHQ, PQS, and their synthetic analogues.^8,9^

So, while SAR studies of the 2-alkyl-4-quinolones have been well-developed in numerous contexts, there are no reports on the corresponding *N*-alkyl-2-quinolones (Figure 1). We initially sought to investigate the bioactivity of *N-*alkyl-2-quinolones in a number of highly relevant bacterial species. We first prepared *N*-alkyl-2-quinolones based on a convenient method described by Lutz and co-workers.^10^ Following alkylation of anthranilic acid, *O*-acetoxy quinolones could be formed by refluxing with acetic anhydride in acetic acid.

**Figure 1:**
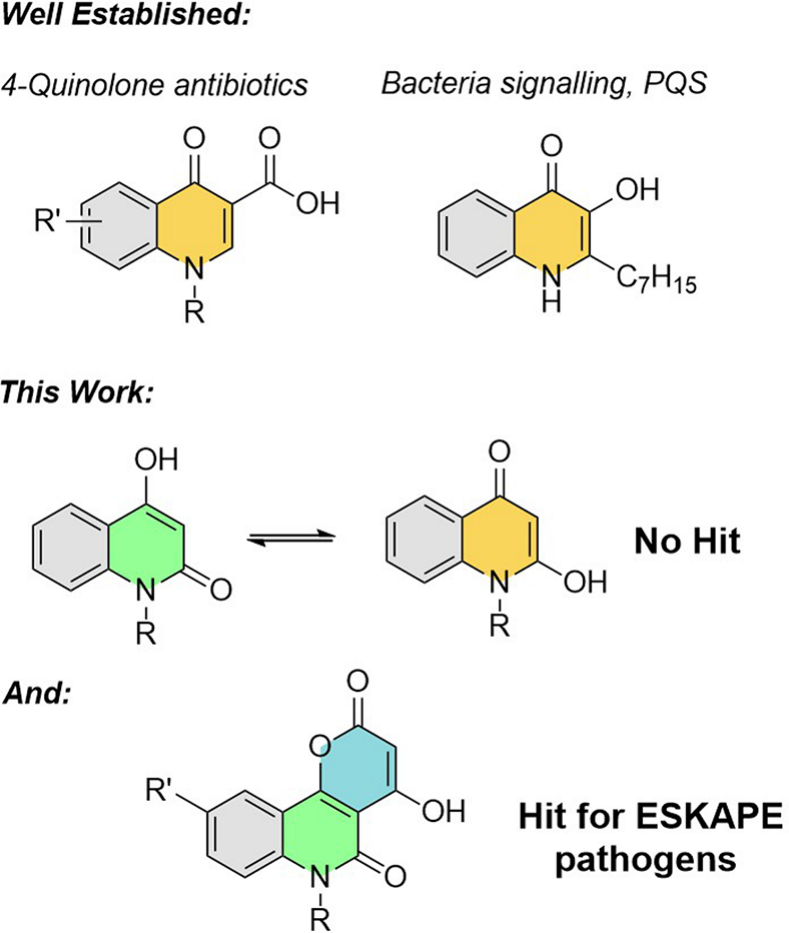
Quinolone derivatives with antimicrobial activity.

This method was not well-suited to the synthesis of analogues about the carbocyclic ring, as substituted anthranilic acid derivatives are not very readily accessible.^11^ Instead, we proposed that substituted *N*-alkyl anilines would react with 1 equiv of diethyl malonate in a high boiling point solvent to give the same quinolone product. However, instead, we observed the formation of tricyclic 4-hydroxy-2*H*-pyrano[3,2-*c*]quinoline-2,5(6*H*)-dione derivatives.^12^ Compounds of this type have been described before, although synthetic studies are limited.^13,14^ Our efforts to prevent “overreaction” with dimalonate were not successful. However, we were presented with convenient access to two different groups of compounds possessing structural features relevant to or required for various bioactivities (see Supporting Information). Thus, compounds **1**–**13** were prepared via reaction of *N*-alkyl amine and diethyl malonate in refluxing diphenylether. Precipitation with hexane produced the desired 4-hydroxy-2*H*-pyrano[3,2-*c*]quinoline-2,5(6*H*)-dione derivatives (Scheme 1**)**.

**Scheme 1:**
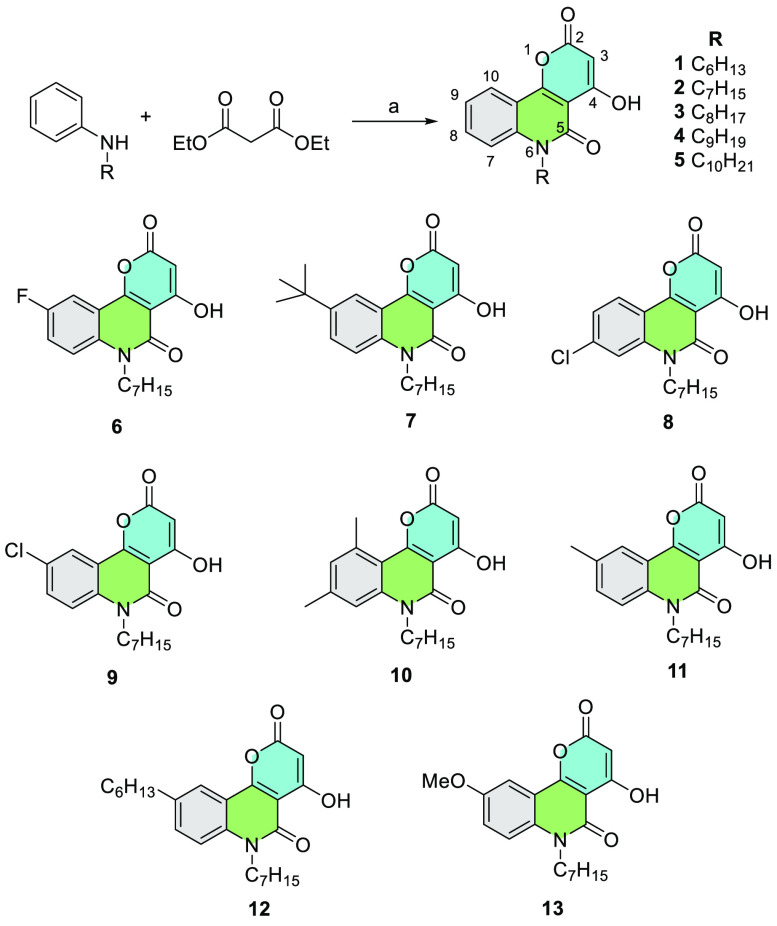
Derivative Compounds Tested for Antibacterial Activity against ESKAPE Pathogens Reagents and conditions: Ph_2_O, reflux, 1 h, 1–99% (see SI).

We first investigated inhibition of growth of a strain of methicillin-resistant *S. aureus* (MRSA) by *N*-alkyl-2-quinolones (10 in total) with different alkyl chain lengths (see Supporting Information). Unfortunately, none of the compounds displayed any antibacterial activity (data not shown).

We then tested pyranoquinolone **2** (Scheme 1) possessing an *n*-heptyl group, as this is a direct analogue of HHQ. Intriguingly, initial observations suggested excellent anti-MRSA activity, which warranted further investigation, both in expanding the types of structures tested and the bacterial strains they were tested against. Thus, we broadened our examination of the tricyclic *N*-alkyl-pyranoquinolone scaffold by alteration of the *N*-alkyl group, affording **1**–**5**. Minimum inhibitory concentrations (MICs) were obtained for a number of *S. aureus* strains, including methicillin-sensitive (MSSA) and -resistant (MRSA) strains, a vancomycin intermediate (VISA) strain, and a clinical daptomycin-resistant isolate (DapRSA) (Table 1). Of the *N*-alkyl-pyranoquinolones tested, the *n-*hexyl, *n-*heptyl, *n-*octyl, and *n-*nonyl analogues (**1**–**4**) all showed better activity than the *n-*decyl analogue (**5**), which was substantially less active against most strains. An *n*-nonyl chain appeared optimal, with compound **4** possessing an MIC of 1–4 μg/mL against all *S. aureus* strains tested.

**Table 1:** MICs for *N*-Alkyl-4-hydroxy-2*H*-pyrano[3,2-*c*]quinoline-2,5(6*H*)-diones in *S. aureus* Strains^a^

	GP_001 ATCC 25923 (MSSA)	GP_020 ATCC 43300 (MRSA)	GP_021 ATCC 33591 (MRSA)	GP_035 ATCC 700699 (MRSA, VISA)	GP_036 Clinical Isolate (MRSA, DapRSA)
compound no.	MIC (μg/mL)				
**1**	16;16	8;8	16;16	8;8	8;8
**2**	8;8	4;4	8;8	8;8	4;4
**3**	8;8	2;2	4;8	4;4	4;8
4	**4;4**	**2;2**	**4;2**	**2;2**	**1;1**
**5**	>64;>64	4;4	>64;>64	>64;>64	>64;>64
**6**	16;8	2;4	8;4	4;4	2;4
7	**2;2**	**1;1**	**2;2**	**1;2**	**1;1**
**9**	8;16	2;1	>128;>128	128;>128	1;1
**10**	>256;>256	>256;>256	>256;>256	n.d.	n.d
**11**	>128;>128	1;1	128;64	>128;>128	>128;128
**12**	>256;>256	>256;>256	>256;>256	n.d.	n.d
**13**	16,8	≤8;≤ 8	≤8;≤ 8	n.d.	n.d

a GP: Gram positive; ATCC: American Type Cell Culture.

The *n-*heptyl tricyclic *N*-alkyl-pyranoquinolone framework was chosen to conduct additional SAR studies by substitution on the carbocyclic ring. In general, substitution at the 9-position was targeted, as this was the most accessible site synthetically. For the new analogues, compound **7**, followed by compound **6** (*t*-Bu and F groups, respectively, at the 9-position) were the most consistently potent across the *S. aureus* strains tested. Both compound **12** (*n*-hexyl group at the 9-position) and compound **10** (8,10-dimethyl) were inactive against the three initial *S. aureus* strains tested.

Interestingly, while the chloro derivative **9** showed poor activity in the GP021 and GP035 strains, it was potent against the other three test strains. Compound **7** stands out as being of interest for further development against MRSA.

It was interesting to note that MRSA strain ATCC 43300 appeared to be generally more susceptible across all the lead molecules. The *cydAB* genes previously shown to underpin susceptibility in *S. aureus* and *S. epidermidis* to the AQ derivative HQNO^15^ were comparable in all test strains, and no ATCC-43300-specific synonymous SNPs were identified in comparison with the test strains.

We then turned to proteomic analysis, which we hoped might provide some insight into the molecular mechanism through which these compounds elicit their growth inhibitory effects. While the majority of proteins found to be differentially encoded in ATCC 43300 when compared with the other test strains were mobile genetic elements (typically transposases), a loss of function mutation in the gene encoding the surface-attached protein SasA was unique to ATCC 43300 (Figure 2). Surface-attached proteins have previously been shown to play a significant role in the host–pathogen interaction and with respect to antimicrobial resistance in *S. aureus*.^16,17^ This will form the basis of further investigations that seek to uncover the molecular mechanism underpinning the differential sensitivity to quinolone derivatives reported in this study. Similarly, previously identified hotspot mutations of the quinolone antibiotic targets GyrA, ParC and ParE were only identified in ATCC 700699 (see Supporting Information).

**Figure 2:**
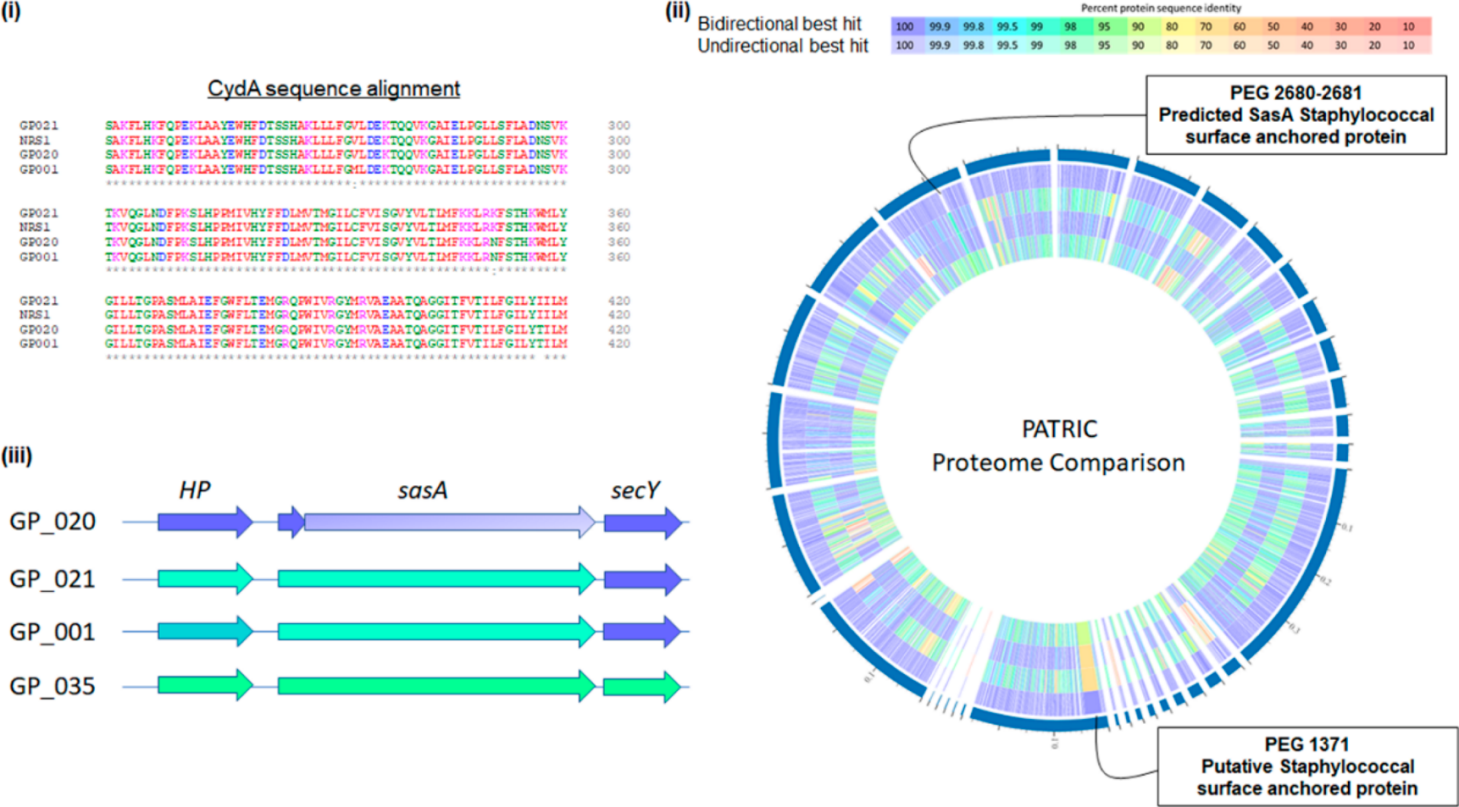
(i) CydA alignment from test strains reveals no ATCC-43300-specific amino acid alterations. (Full sequence alignments provided in Supporting Information). (ii) PATRIC proteome alignment analysis of the *Staphylococcus aureus* strains used in this study. List of tracks, from outside to inside: GP_020: *S. aureus* strain ATCC 43300; GP_021: *S. aureus* strain ATCC 33591; GP_001: *S. aureus* strain ATCC 25923; GP_035: *S. aureus* strain NRS1. (iii) Genome comparisons reveal loss of function variant of SasA surface-attached protein in GP_020.

To determine if the scope of activity was specific for the *S. aureus* species, MIC testing was broadened to test other ESKAPE pathogens (Table 2). It was observed that several compounds exhibited activity against some *Staphylococcus epidermidis* and *Enterococcus* spp. strains. In particular, compound **7** showed excellent potency against the *S. epidermidis* GP_033 (VISE) strain. While MICs against *Enterococcus* sp. GP_024 (Type Strain) were quite poor, *n*-decyl analogue **5**, which had not been noteworthy in other assays, was quite potent against the GP_026 (VRE) strain.

**Table 2:** MICs for Pyranoquinolines in Other Pathogenic Species

	*Enterococcus* spp.		*S. epidermidis*	
compound no.	GP_024 ATCC 35667 (type strain)	GP_026 ATCC 700221 (VRE)	GP_017 ATCC 12228 (PCI 1200 NRS 231)	GP_033 NRS 60 (VISE)
	MIC (μg/mL)			
**1**	>64;>64	16;8	8;8	2;8
**2**	>128;128	16;16	8;4	4;8
**3**	>128; 128	4;1	4;4	1;4
**4**	>4;>64	4;4	2;2	2;2
**5**	>64;>64	**2;0.5**	2;4	>64;>64
**6**	>64;>64	32;32	4;4	8;8
**7**	>128;>128	4;4	2;2	**0.25;0.5**
**9**	>128;>128	64;64	2;1	8;8
**11**	128;128	32;64	16;16	64;>128

In conclusion, we demonstrated very good antibacterial activity across a range of bacteria including a number of MRSA strains. Activity is underpinned by a relatively underexplored tricyclic 4-hydroxy-2*H*-pyrano[3,2-*c*]quinoline-2,5(6*H*)-dione core. A potent analogue with an *N*-heptyl-9-*t*-Bu substitution pattern emerged as a hit with MIC levels ≤2 μg/mL across five strains of *S. aureus*, including resistant isolates. In addition, the same compound proved highly potent against *Enterococcus* spp. (0.25 μg/mL).

Activity was observed at the species and strain level, perhaps unsurprising given the extensive phenotypic and genotypic heterogeneity evolving within microbial populations.

Finally, while the activity is biocidal, it is nevertheless interesting that the framework with longer alkyl chains (akin to signaling compounds such as PQS (Figure 1)) gives the best biological activity. Further investigation of the mechanisms of action of the compounds described herein may offer some valuable insights, and a precise approach where opportunistic pathogens are disarmed, or specifically targeted at species or strain level, could improve the clinical management of infection in the future.

*Chemical synthesis*. Details of the chemical synthesis and characterization of compounds are provided in the supplementary data.

*MIC screening*. Details of strains, culture conditions, and MIC screening protocols are contained within the supplementary data.

*Comparative genomic analysis*. Genome sequences were accessed through the PATRIC informatics interface.^18^ Outputs were exported to Excel and screened visually for unique genes. BLAST analysis through the PATRIC platform enabled identification of CydAB proteins in test genomes, and alignments were performed using Clustal Omega.

## Supporting Information Available

Biological data and details of the chemical synthesis, characterization data, and NMR spectra (PDF)

## Abbreviations

PQS: 2-heptyl-3,4-dihydroxyquinoline (Pseudomonas quinolone signal)
HHQ: 4-hydroxy-2-heptyl-quinoline
MRSA: methicillin-resistant *Staphylococcus aureus*
VRE: vancomycin-resistant *Enterococcus*
MIC: minimum inhibitory concentration
VISE: vancomycin intermediate *Staphylococcus epidermidis*
