# Supplementary material for: N-Alkyl-2-Quinolonopyrones Demonstrate Antimicrobial Activity against ESKAPE Pathogens Including Staphylococcus aureus
Source: ACS Med Chem Lett. 2022 Jul 19;13(8):1358–62. doi: 10.1021/acsmedchemlett.2c00185 (PMC9377017; doi:10.1021/acsmedchemlett.2c00185)
Supplement: Supplementary file 1 — ml2c00185_si_001.pdf [file ml2c00185_si_001.pdf]

N-Alkyl-2-quinolonopyrones demonstrate antimicrobial activity against ESKAPE pathogens including Staphylococcus aureus.

Eoin Moynihan,[a] Katrina Mackey,[a] Mark A.T. Blaskovich,[b] F. Jerry Reen*,[c] Gerard McGlacken*[a]

[a]School of Chemistry and Analytical and Biological Chemistry Research Facility, University College Cork, Cork, Ireland. [b]Community for Open Antimicrobial Drug Discovery, Centre for Superbug Solutions, Institute for Molecular Bioscience, The University of Queensland, St. Lucia, Queensland 4072, Australia. [c]School of Microbiology, University College Cork, Cork, Ireland.

*Email: Dr Gerard P. McGlacken g.mcglacken@ucc.ie

Dr F. Jerry Reen j.reen@ucc.ie

Table Of Contents

**Content**	**Page**
General Information	S2
Synthesis of pyranoquinolones and N-alkyl-4-hydroxy-2(1*H*)-quinolones	S3-S10
^{1}H and ^{13}C NMR spectra	S11-S46
Biological Data	S47-S55
Biological Methods	S56-S60
References	S61

Supplementary Chemical Synthesis and Data

General Information

Melting point determinations were performed by the open capillary method and are reported uncorrected. ^{1}H and ^{13}C NMR spectra were recorded at 300 K in $CDCl_3$ at 300 and 75 MHz spectrometer unless otherwise specified, with tetramethylsilane (TMS) as the internal standard. Chemical shifts (δ_H and δ_C) were expressed as parts per million (ppm) positive shift being downfield from TMS; coupling constants (*J*) are expressed in hertz (Hz). High-resolution mass spectra (HRMS) were obtained on a TOF MS instrument with ESI source. High-resolution mass spectra were recorded only for new compounds. Literature citations are provided for known compounds and representative characterisation data. IR spectra were recorded on an FT-IR spectrometer as a thin film (liquid samples) or applied as a solution in chloroform, and the chloroform was allowed to evaporate (solid samples). Column chromatography was carried out using 60 Å (35–70 µm) silica. Reaction solvents were used as bulk and not pre-dried.

General method for synthesis of pyranoquinolones

An *N*-alkylated aniline[1-7] and diethyl malonate (1 equiv.) were mixed together in a syringe. Diphenylether (0.7 mL/mmol) was heated to reflux under N_2 and the mixture of reagents was added dropwise. The reaction mixture was then allowed to reflux for a further 1h and then allowed to cool to room temperature. Hexane was added until precipitation was observed and the flask was placed in a refrigerator overnight. The precipitate was isolated by filtration, washed copiously with hexane and dried under vacuum.

Note: When precipitation was not observed, the products were isolated by column chromatography (silica gel, 95:5 DCM/MeOH). Yields are given with regard to the aniline being the limiting reagent. The yield of compound **9** is given over 2 steps and is notably low due to difficulty in purification of the *N*-alkyl aniline.

6-Hexyl-4-hydroxy-2*H*-pyrano[3,2-*c*]quinoline-2,5(6*H*)-dione, 1

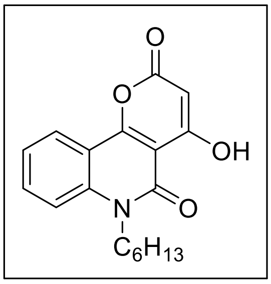

yellow solid (0.171 g, 62%). mp 126-128 °C; IR (NaCl) ν_{max}: 3458 (br., OH), 2932, 2858 (C-H stretch), 1746, 1678 (C=O stretch), 1574, 1556 (C=C stretch) cm^{-1}; ^{1}H NMR (300 MHz, $CDCl_3$): δ 13.25 (1H, s), 8.35 (1H, dd, *J* = 8.1, 1.5 Hz), 7.78 (1H, ddd, *J* = 7.9, 1.5, 1.5 Hz), 7.40-7.53 (2H, m), 5.67 (1H, s), 4.33 (2H, t, *J* = 7.9 Hz), 1.71-1.85 (2H, m), 1.25-1.54 (6H, m), 0.92 (3H, t, *J* = 7.2 Hz); ^{13}C NMR (75 MHz, $CDCl_3$): δ 169.0, 162.8, 161.5, 159.3, 138.2, 134.1, 125.0, 124.0, 115.1, 114.0, 100.0, 90.9, 42.6, 31.4, 27.6, 26.6, 22.5, 14.0; MS (ESI) *m/*z: 314 [$(M+H)^+$, 100%], HRMS (ESI): *m/*z calcd for $C_{18}H_{20}NO_4$ [$M+H^+$]: 314.1392, found 314.1388.

6-Heptyl-4-hydroxy-2*H*-pyrano[3,2-*c*]quinoline-2,5(6*H*)-dione, 2

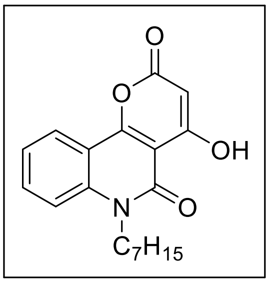

yellow solid (0.180 g, 75%). mp 119-121 °C; IR (NaCl) ν_{max}: 2955, 2925, 2856 (C-H stretch), 1748, 1673 (C=O stretch), 1571 (C=C stretch) cm^{-1}; ^{1}H NMR (300 MHz, $CDCl_3$): δ 13.25 (1H, s), 8.35 (1H, dd, *J* = 8.1, 1.5 Hz), 7.78 (1H, ddd, *J* = 8.0, 1.6, 1.5 Hz), 7.41-7.53 (2H, m), 5.67 (1H, s), 4.33 (2H, t, *J* = 8.0 Hz), 1.71-1.85 (2H, m), 1.22-1.54 (8H, m), 0.90 (3H, t, *J* = 7.0 Hz); ^{13}C NMR (75 MHz, $CDCl_3$): δ 169.0,

162.9, 161.5, 159.3, 138.2, 134.1, 125.0, 124.0, 115.1, 114.0, 100.0, 90.9, 42.6, 31.7, 28.9, 27.6, 26.9, 22.5, 14.0; MS (ESI) *m/*z: 328 [(M+H)$^+$, 100%], HRMS (ESI): *m/*z calcd for $C_{19}H_{22}NO_4$ [M+H$^+$]: 328.1549, found 328.1544.

4-Hydroxy-6-octyl-2*H*-pyrano[3,2-*c*]quinoline-2,5(6*H*)-dione, 3

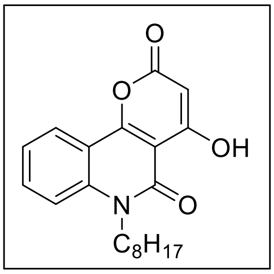

yellow solid (0.072 g, 51%). mp 120-122 °C, IR (NaCl) ν_{max}: 2921, 2853 (C-H stretch), 1740, 1677 (C=O stretch), 1574 (C=C stretch) cm^{-1}; ^{1}H NMR (300 MHz, $CDCl_3$): δ 12.99 (1H, br. s), 8.35 (1H, dd, *J* = 8.1, 1.5 Hz), 7.78 (1H, ddd, *J* = 8.0, 1.6, 1.5 Hz), 7.39-7.25 (2H, m), 5.67 (1H, s), 4.33 (2H, t, *J* = 8.1 Hz), 1.71-1.85 (2H, m), 1.21-1.51 (10H, m), 0.89 (3H, t, *J* = 6.8 Hz); ^{13}C NMR (75 MHz, $CDCl_3$): δ 169.0, 162.9, 161.5, 159.3, 138.2, 134.1, 125.0, 124.1, 115.1, 114.1, 100.0, 90.9, 42.6, 31.7, 29.2, 29.1, 27.6, 26.9, 22.6, 14.0; MS (ESI) *m/*z: 342 [(M+H)$^+$, 100%], HRMS (ESI): *m/*z calcd for $C_{20}H_{24}NO_4$ [M+H$^+$]: 342.1705, found 342.1700.

4-Hydroxy-6-nonyl-2*H*-pyrano[3,2-*c*]quinoline-2,5(6*H*)-dione, 4

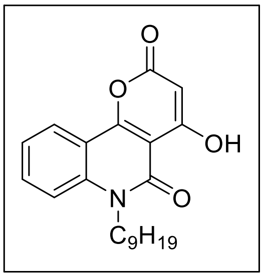

yellow solid (0.023 g, 14%). mp 116-118 °C; IR (NaCl) ν_{max}: 2920, 2854 (C-H stretch), 1738, 1675 (C=O stretch), 1574 (C=C stretch) cm^{-1}; ^{1}H NMR (300 MHz, $CDCl_3$): δ 13.25 (1H, br. s), 8.33 (1H, dd, *J* = 8.2, 1.4 Hz), 7.78 (1H, ddd, *J* = 7.9, 1.5, 1.5 Hz), 7.40-7.53 (2H, m), 5.66 (1H, s), 4.33 (2H, t, *J* = 7.8 Hz), 1.71-1.86 (2H, m), 1.17-1.55 (12H, m), 0.88 (3H, t, *J* = 7.2 Hz); ^{13}C NMR (75 MHz, $CDCl_3$): δ 169.0, 162.9, 161.5, 159.4, 138.2, 134.0, 125.1, 124.0, 115.1, 114.1, 100.0, 90.9, 42.6, 31.8, 29.4, 29.3, 29.2, 27.6, 26.9, 22.6, 14.1; MS (ESI) *m/*z: 356 [(M+H)$^+$, 100 %], HRMS (ESI): *m/*z calcd for $C_{21}H_{26}NO_4$ [M+H$^+$]: 356.1862, found 356.1848.

6-Decyl-4-hydroxy-2*H*-pyrano[3,2-*c*]quinoline-2,5(6*H*)-dione, 5

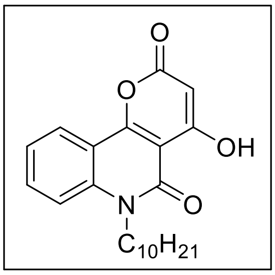

yellow solid (0.132 g, 85%). mp 108-110 °C; IR (NaCl) ν_{max}: 3446 (br., OH), 2921, 2851 (C-H stretch), 1733, 1671 (C=O stretch), 1576 (C=C stretch) cm^{-1}; ^{1}H NMR (300 MHz, $CDCl_3$): δ 13.25 (1H, br s), 8.35 (1H, dd, *J* = 8.3, 1.4 Hz), 7.78 (1H, ddd, *J* = 7.9, 1.6, 1.5 Hz), 7.40-7.53 (2H, m), 5.67 (1H, s), 4.33 (2H, t, *J* = 7.9 Hz), 1.72-1.84 (2H, m), 1.19-1.52 (14H, m), 0.88 (3H, t, *J* = 7.0 Hz); ^{13}C NMR (75 MHz, $CDCl_3$): δ 169.0, 162.9, 161.6, 159.4, 138.2, 134.0, 125.1, 124.0, 115.1, 114.1, 100.1, 90.9, 42.6, 31.8, 29.5, 29.3, 27.6, 26.9, 22.7, 14.1; MS (ESI) *m/*z: 370 [(M+H)$^+$, 100%]; HRMS (ESI): *m/*z calcd for $C_{22}H_{28}NO_4$ [M+H$^+$]: 370.2008, found 370.2005.

9-Fluoro-6-heptyl-4-hydroxy-2*H*-pyrano[3,2-*c*]quinoline-2,5(6*H*)-dione, 6

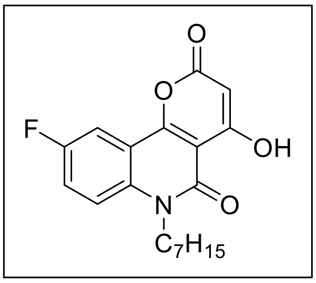

yellow solid (0.277 g, 85%). mp 123-125 °C; IR (NaCl) ν_{max}: 2929, 2857 (C-H stretch), 1753, 1672 (C=O stretch), 1565 (C=C stretch), 1424 (C-F) cm^{-1}; ^{1}H NMR (300 MHz, $CDCl_3$): δ 13.22 (1H, s), 8.01 (1H, dd, *J* = 8.3, 2.5 Hz), 7.44-7.58 (2H, m), 5.69 (1H, s), 4.32 (2H, t, *J* = 7.9 Hz), 1.70-1.85 (2H, m), 1.16-1.54 (8H, m), 0.90 (3H, t, *J* = 6.8 Hz); ^{13}C NMR (75 MHz, $CDCl_3$): δ 168.7, 162.5, 161.2, 158.7 (d, ^{1}J = 247 Hz), 158.4, 134.7 (d, ^{4}J = 2 Hz), 122.3 (d, ^{2}J = 24 Hz), 117.2 (d, ^{3}J = 8 Hz), 115.1 (d, ^{3}J = 9 Hz), 110.4 (d, ^{2}J = 24 Hz), 100.8, 91.5, 42.9, 31.7, 28.9, 27.7, 26.8, 22.6, 14.1; MS (ESI) *m/*z: 346 [(M+H)$^+$, 100%], HRMS (ESI): *m/*z calcd for $C_{19}H_{21}FNO_4$ [M+H$^+$]: 346.1455, found 346.1458.

9-(*tert*-Butyl)-6-heptyl-4-hydroxy-2*H*-pyrano[3,2-*c*]quinoline-2,5(6*H*)-dione, 7

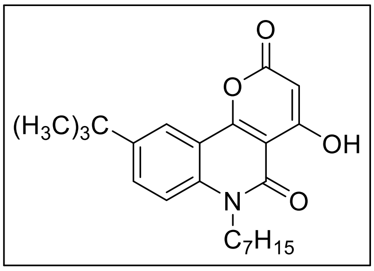

yellow solid (0.132 g, 48%). mp 117-120 °C; IR (NaCl) ν_{max}: 3449 (br, OH), 2924 (C-H stretch), 1729, 1675 (C=O stretch), 1574 (C=C stretch) cm^{-1}; ^{1}H NMR (300 MHz, $CDCl_3$): δ 13.40 (1H, s), 8.28 (1H, d, *J* = 2.4 Hz), 7.84 (1H, dd, *J* = 9.0, 2.4 Hz), 7.44 (1H, d, *J* = 9.1 Hz), 5.66 (1H, s), 4.32 (2H, t, *J* = 7.9 Hz), 1.71-1.86 (2H, m), 1.23-1.53 (17H, m), 0.90 (3H, t, *J* = 6.9 Hz); ^{13}C NMR (75 MHz, $CDCl_3$): δ 169.3, 162.7, 161.8, 159.6, 147.5, 136.2, 132.1, 120.9, 115.0,

113.7, 99.9, 90.7, 42.5, 34.8, 31.7, 31.2, 28.9, 27.7, 26.9, 22.6, 14.0; MS (ESI) *m/z*: 384 [(M+H)$^+$, 100%], HRMS (ESI): *m/z* calcd for $C_{23}H_{30}NO_4$ [M+H$^+$]: 384.2175, found 384.2172.

8-Chloro-6-heptyl-4-hydroxy-2*H*-pyrano[3,2-*c*]quinoline-2,5(6*H*)-dione, 8

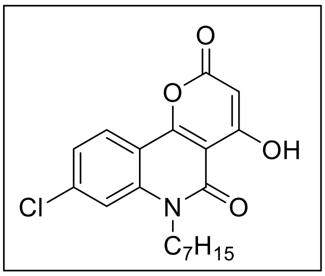

brown solid (0.048 g, 40%). mp 125-127 °C; IR (NaCl) ν_{max}: 2925 (C-H stretch), 1756, 1672 (C=O stretch), 1566 (C=C stretch) cm^{-1}; ^{1}H NMR (300 MHz, $CDCl_3$): δ 12.99 (1H, s), 8.25 (1H, d, *J* = 8.7 Hz), 7.47 (1H, s), 7.41 (1H, d, *J* = 8.8 Hz), 5.65 (1H, s), 4.28 (2H, t, *J* = 8.1 Hz), 1.69-1.85 (2H, m), 1.28-1.55 (8H, m), 0.90 (3H, t, *J* = 6.7 Hz); ^{13}C NMR (75 MHz, $CDCl_3$): δ 168.7, 162.9, 161.1, 158.9, 140.7, 138.9, 126.3, 124.7, 115.2, 112.2, 100.0, 91.1, 42.8, 31.7, 28.9, 27.5, 26.8, 22.5, 14.0; MS (ESI) *m/z*: 362 [(M+H)$^+$, 100%], HRMS (ESI): *m/z* calcd for $C_{19}H_{21}ClNO_2$ [M+H$^+$]: 362.1159, found 362.1162.

9-Chloro-6-heptyl-4-hydroxy-2*H*-pyrano[3,2-*c*]quinoline-2,5(6*H*)-dione, 9

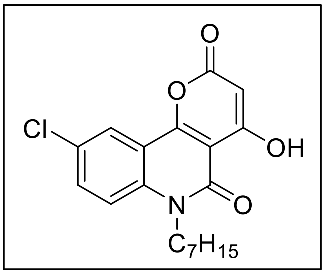

brown solid (0.040 g, 1% (over 2 steps)). mp 129-131 °C; IR (NaCl) ν_{max}: 3455 (br., OH), 2924, 2854 (C-H stretch), 1734, 1673 (C=O stretch) cm^{-1}; ^{1}H NMR (300 MHz, $CDCl_3$): δ 13.11 (1H, s), 8.32 (1H, d, *J* = 2.5 Hz), 7.72 (1H, dd, *J* = 9.2, 2.5 Hz), 7.43 (1H, d, *J* = 9.2 Hz), 5.69 (1H, s), 4.30 (2H, t, *J* = 8.0 Hz), 1.69-1.84 (2H, m), 1.18-1.52 (8H, m), 0.90 (3H, t, *J* = 6.8 Hz); ^{13}C NMR (75 MHz, $CDCl_3$): δ 168.7, 162.6, 161.1, 158.2, 136.6, 134.2, 130.2, 124.3, 116.7, 115.1, 100.7, 91.5, 42.1, 31.7, 28.9, 27.6, 26.8, 22.6, 14.1; MS (ESI) *m/z*: 362 [(M+H)$^+$, 100%], HRMS (ESI): *m/z* calcd for $C_{19}H_{21}ClNO_4$ [M+H$^+$]: 362.1159, found 362.1161.

6-Heptyl-4-hydroxy-8,10-dimethyl-2*H*-pyrano[3,2-*c*]quinoline-2,5(6*H*)-dione, 10

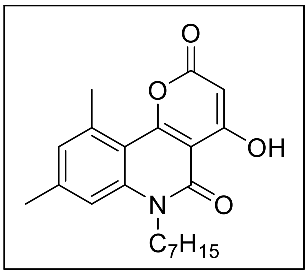

tan solid (0.169 g, 75%). mp 124-127 °C; IR (NaCl) ν_{max}: 3459 (br., OH), 2928 (C-H stretch), 1747, 1667 (C=O stretch), 1556 (C=C stretch) cm^{-1}; ^{1}H NMR (300 MHz, $CDCl_3$): δ 13.68 (1H, s), 7.14 (1H, s), 7.06 (1H, s), 5.62 (1H, s), 4.32 (2H, t, *J* = 8.0 Hz), 2.94 (3H, s), 2.51 (3H, s), 1.70-1.84 (2H, m), 1.20-1.53 (8H, m), 0.90 (3H, t, *J* = 6.8 Hz); ^{13}C NMR (75 MHz, $CDCl_3$): δ 169.6, 162.9, 161.7, 161.6, 144.3, 139.8, 139.5, 129.4, 113.4, 111.1, 99.3, 89.9, 42.9, 31.7, 28.9, 27.4, 26.8, 24.7, 22.6, 22.3, 14.0; MS (ESI) *m/*z: 356 [(M+H)$^+$, 100%], HRMS (ESI): *m/*z calcd for $C_{21}H_{26}NO_4$ [M+H$^+$]: 356.1862, found 356.1857.

6-Heptyl-4-hydroxy-9-methyl-2*H*-pyrano[3,2-*c*]quinoline-2,5(6*H*)-dione, 11

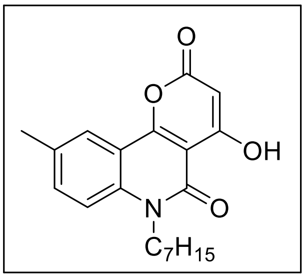

yellow solid (0.277 g, 82%).mp 126-128 °C; IR (NaCl) ν_{max}: 2926, 2856 (C-H stretch), 1745, 1676 (C=O stretch) cm^{-1}; ^{1}H NMR (300 MHz, $CDCl_3$): δ 13.36 (1H, s), 8.15 (1H, d, *J* = 1.6 Hz), 7.59 (1H, dd, *J* = 8.9, 2.1 Hz), 7.38 (1H, d, *J* = 8.9 Hz), 5.66 (1H, s), 4.31 (2H, t, *J* = 7.9 Hz), 2.49 (3H, s), 1.70-1.85 (2H, m), 1.25-1.52 (8H, m), 0.90 (3H, t, *J* = 6.8 Hz); ^{13}C NMR (75 MHz, $CDCl_3$): δ 169.2, 162.6, 161.7, 159.1, 136.3, 135.5, 134.2, 124.6, 115.0, 113.9, 99.9, 90.7, 42.5, 31.7, 29.0, 27.7, 26.9, 22.6, 20.8, 14.1; MS (ESI) *m/*z: 342 [(M+H)$^+$, 100%], HRMS (ESI): *m/*z calcd for $C_{20}H_{24}NO_4$ [M+H$^+$]: 342.1705, found 342.1705.

6-Heptyl-9-hexyl-4-hydroxy-2*H*-pyrano[3,2-*c*]quinoline-2,5(6*H*)-dione, 12

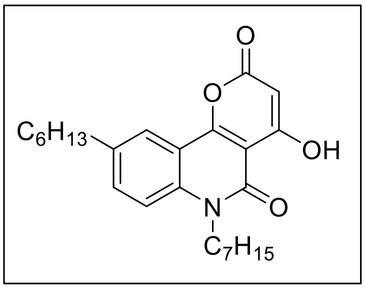

yellow solid (0.201 g, 99%). mp 121-123 °C; IR (NaCl) ν_{max}: 3451 (br., OH), 2925 (C-H stretch), 1730, 1673 (C=O stretch), 1576 (C=C stretch) cm^{-1}; ^{1}H NMR (300 MHz, $CDCl_3$): δ 13.38 (1H, s), 8.13 (1H, d, *J* = 1.9 Hz), 7.60 (1H, dd, *J* = 8.8, 2.1 Hz), 7.40 (1H, d, *J* = 8.8 Hz), 5.65 (1H, s), 4.31 (2H, t, *J* = 7.7 Hz), 2.74 (2H, t, *J* = 7.8 Hz), 1.61-1.84 (4H, m), 1.20-1.53 (14H, m), 0.83-0.97 (6H, m); ^{13}C NMR (75 MHz, $CDCl_3$): δ 169.3, 162.7, 161.8, 159.6, 139.3, 136.5, 134.9, 124.0, 115.0, 114.0, 99.9, 90.7, 42.6, 31.7, 31.6, 31.3, 28.9, 28.9, 26.9, 22.6, 14.0; MS

(ESI) *m/*z: 412 [$(M+H)^+$, 100%], HRMS (ESI): *m/*z calcd for $C_{25}H_{34}NO_4$ [$M+H^+$]: 412.2488, found 412.2483.

6-Heptyl-4-hydroxy-9-methoxy-2*H*-pyrano[3,2-*c*]quinoline-2,5(6*H*)-dione, 13

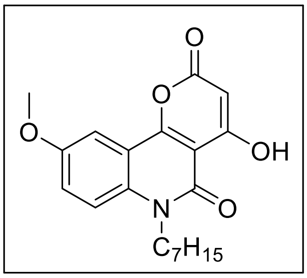

yellow solid (0.036 g, 32%). mp 132-135 °C; IR (NaCl) ν_{max}: 2931 (C-H stretch), 1741, 1674 (C=O stretch) cm^{-1}; ^1H NMR (300 MHz, $CDCl_3$): δ 13.53 (1H, s), 7.60 (1H, d, *J* = 2.6 Hz), 7.34-7.48 (2H, m), 5.67 (1H, s), 4.31 (2H, t, *J* = 7.9 Hz), 3.94 (3H, s), 1.70-1.83 (2H, m), 1.22-1.50 (8H, m), 0.89 (3H, t, *J* = 6.7 Hz); ^{13}C NMR (75 MHz, $CDCl_3$): δ 169.4, 162.1, 161.8, 158.7, 156.1, 132.8, 124.7, 116.7, 114.8, 104.7, 100.2, 90.9, 56.1, 42.7, 31.7, 29.0, 27.8, 26.9, 22.6, 14.1; MS (ESI) *m/*z: 358 [$(M+H)^+$, 100%], HRMS (ESI): *m/*z calcd for $C_{20}H_{24}NO_5$ [$M+H^+$]: 358.1654, found 358.1649.

<u>General method for synthesis of N-alkyl-4-hydroxy-2(1*H*)-quinolones</u>

A mixture of an N-alkyl-anthranilic acid[8-10] and acetic anhydride (4.8 equiv.) in acetic acid (2.2 M) was stirred at reflux under N_2 for 4 h. The reaction mixture was allowed to cool, was poured onto ice and 6M aqueous sodium hydroxide solution was then added until pH 10 was achieved. This mixture was allowed to stir at room temperature for 30 min. Then, concentrated aqueous HCl was slowly added until pH 5 was achieved. The mixture was allowed to cool to room temperature and the formed precipitate was isolated by filtration. The solid was precipitated by trituration using hot ethyl acetate and purified by recrystallisation using hot MeOH.

***N*-Hexyl-4-hydroxy-2(1*H*)-quinolone, 14**

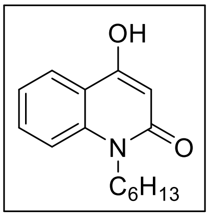

Spectral characteristics were consistent with previously reported data.[11] brown solid (0.518 g, 33%) mp 162-164 °C (lit.[11] 184-186 °C); ^{1}H NMR (300 MHz, DMSO-*δ6*): δ 7.97 (1H, d, *J* = 7.4 Hz), 7.52 (1H, t, *J* = 7.3 Hz), 7.36 (1H, d, *J* = 8.5 Hz), 7.12 (1H, t, *J* = 7.4 Hz), 5.63 (1H, s), 4.10 (2H, t, *J* = 7.6 Hz), 1.47-1.65 (2H, m), 1.22-1.43 (6H, m), 0.86 (3H, t, *J* = 6.5 Hz); ^{13}C NMR (75 MHz, DMSO-*δ6*): δ 163.5, 163.3, 139.6, 131.4, 124.1, 121.1, 118.1, 114.6, 97.7, 39.5, 31.5, 27.8, 26.4, 22.5, 14.4; MS (ESI) *m/*z: 244 [(M-H)$^-$, 40%].

***N*-Heptyl-4-hydroxy-2(1*H*)-quinolone, 15**

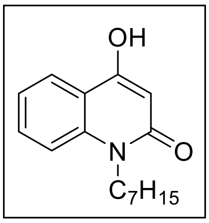

Spectral characteristics were consistent with previously reported data.[12] white solid (0.033 g, 7%). mp 132-134 °C, ^{1}H NMR (300 MHz, $CDCl_3$): δ 8.00 (1H, dd, *J* = 8.0, 1.4 Hz), 7.44 (1H, ddd, *J* = 7.9, 1.5, 1.4 Hz), 7.16 (1H, t, *J* = 7.6 Hz), 7.02 (1H, d, *J* = 8.6 Hz), 6.13 (1H, s), 3.85 (2H, t, *J* = 7.8 Hz), 1.47-1.68 (2H, m), 1.17-1.45 (8H, m), 0.88 (3H, t, *J* = 6.9 Hz); ^{13}C NMR (75 MHz, $CDCl_3$): δ 165.2, 163.7, 138.5, 130.8, 124.2, 121.6, 117.6, 114.3, 96.8, 42.1, 31.8, 30.0, 27.6, 26.9, 22.6, 14.1; MS (ESI) *m/*z: 260 [(M+H)$^+$, 100%], HRMS (ESI): *m/*z calcd for $C_{16}H_{22}NO_2$ [M+H$^+$]: 260.1651, found 260.1663.

***N*-Octyl-4-hydroxy-2(1*H*)-quinolone, 16**

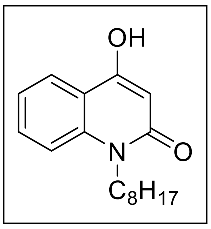

beige solid (0.124 g, 29%). mp 152-153 °C, IR (KBr) ν_{max}: 3433 (br., OH), 2927, 2742 (C-H stretch), 1634 (C=O stretch), 1609, 1566 (C=C stretch) cm^{-1}; ^{1}H NMR (300 MHz, $CDCl_3$): δ 8.00 (1H, dd, *J* = 8.0, 1.5 Hz), 7.45 (1H, ddd, *J* = 7.9, 1.5, 1.5 Hz), 7.16 (1H, t, *J* = 7.2 Hz), 7.04 (1H, d, *J* = 8.7 Hz), 6.13 (1H, s), 3.86 (2H, t, *J* = 8.5 Hz), 1.49-1.65 (2H, m), 1.16-1.44 (10H, m), 0.87 (3H, t, *J* = 7.0 Hz); ^{13}C NMR (75 MHz, $CDCl_3$): δ 165.1, 163.6, 138.5, 130.9, 124.2, 121.6, 117.6, 114.3, 96.9, 31.8, 29.3, 29.2, 27.6, 26.9, 22.6, 14.1; MS (ESI) *m/*z: 274 [(M+H)$^+$, 100%], HRMS (ESI): *m/*z calcd for $C_{17}H_{24}NO_2$ [M+H$^+$]: 274.1807, found 274.1804.

_N_-Nonyl-4-hydroxy-2(1_H_)-quinolone, 17

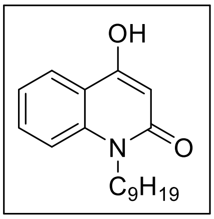

yellow solid (0.171 g, 38%). mp 147-149 °C; IR (KBr) ν_{max}: 3387 (br., OH), 2924, 2853 (C-H stretch), 1634 (C=O stretch), 1609, 1565 (C=C stretch) cm^{-1}; ^{1}H NMR (300 MHz, $CDCl_3$): δ 12.71 (1H, br. s), 8.01 (1H, dd, *J* = 8.1, 1.4 Hz), 7.46 (1H, ddd, *J* = 7.9, 1.5, 1.4 Hz), 7.16 (1H, t, *J* = 7.7 Hz), 7.06 (1H, d, *J* = 8.6 Hz), 6.14 (1H, s), 3.88 (2H, t, *J* = 7.7 Hz), 1.48-1.70 (2H, m), 1.16-1.45 (12H, m), 0.87 (3H, t, *J* = 6.9 Hz); ^{13}C NMR (75 MHz, $CDCl_3$): δ 165.1, 163.7, 138.5, 130.8, 124.2, 121.6, 117.6, 114.3, 96.9, 42.1, 31.9, 29.5, 29.3, 27.6, 26.9, 22.7, 14.1; MS (ESI) *m/z*: 288 [$(M+H)^+$, 100%], HRMS (ESI): *m/z* calcd for $C_{18}H_{26}NO_2$ [$M+H^+$]: 288.1964, found 288.1958.

_N_-Decyl-4-hydroxy-2(1_H_)-quinolone, 18

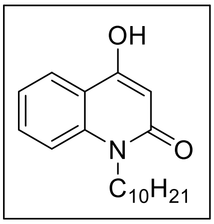

yellow solid (0.161 g, 38%). mp 142-144 °C; IR (KBr) ν_{max}: 2920, 2849 (C-H stretch), 1648 (C=O stretch), 1637, 1515 (C=C stretch) cm^{-1}; ^{1}H NMR (300 MHz, $CDCl_3$): δ 8.01 (1H, dd, *J* = 8.3, 1.4 Hz), 7.46 (1H, ddd, *J* = 7.8, 1.5, 1.5 Hz), 7.16 (1H, t, *J* = 7.5 Hz), 7.06 (1H, d, *J* = 8.6 Hz), 6.15 (1H, s), 3.89 (2H, t, *J* = 8.0 Hz), 1.50-1.68 (2H, m), 1.14-1.44 (14H, m), 0.87 (3H, t, *J* = 7.1 Hz); ^{13}C NMR (75 MHz, $CDCl_3$): δ 165.1, 163.7, 138.5, 130.8, 124.2, 121.6, 117.6, 114.2, 96.9, 42.1, 31.9, 29.6, 29.6, 29.3, 29.3, 27.6, 26.9, 22.7, 14.1; MS (ESI) *m/z*: 302 [$(M+H)^+$, 100%], HRMS (ESI): *m/z* calcd for $C_{19}H_{28}NO_2$ [$M+H^+$]: 302.2120, found 302.2112.

^{1}H and ^{13}C NMR Spectra

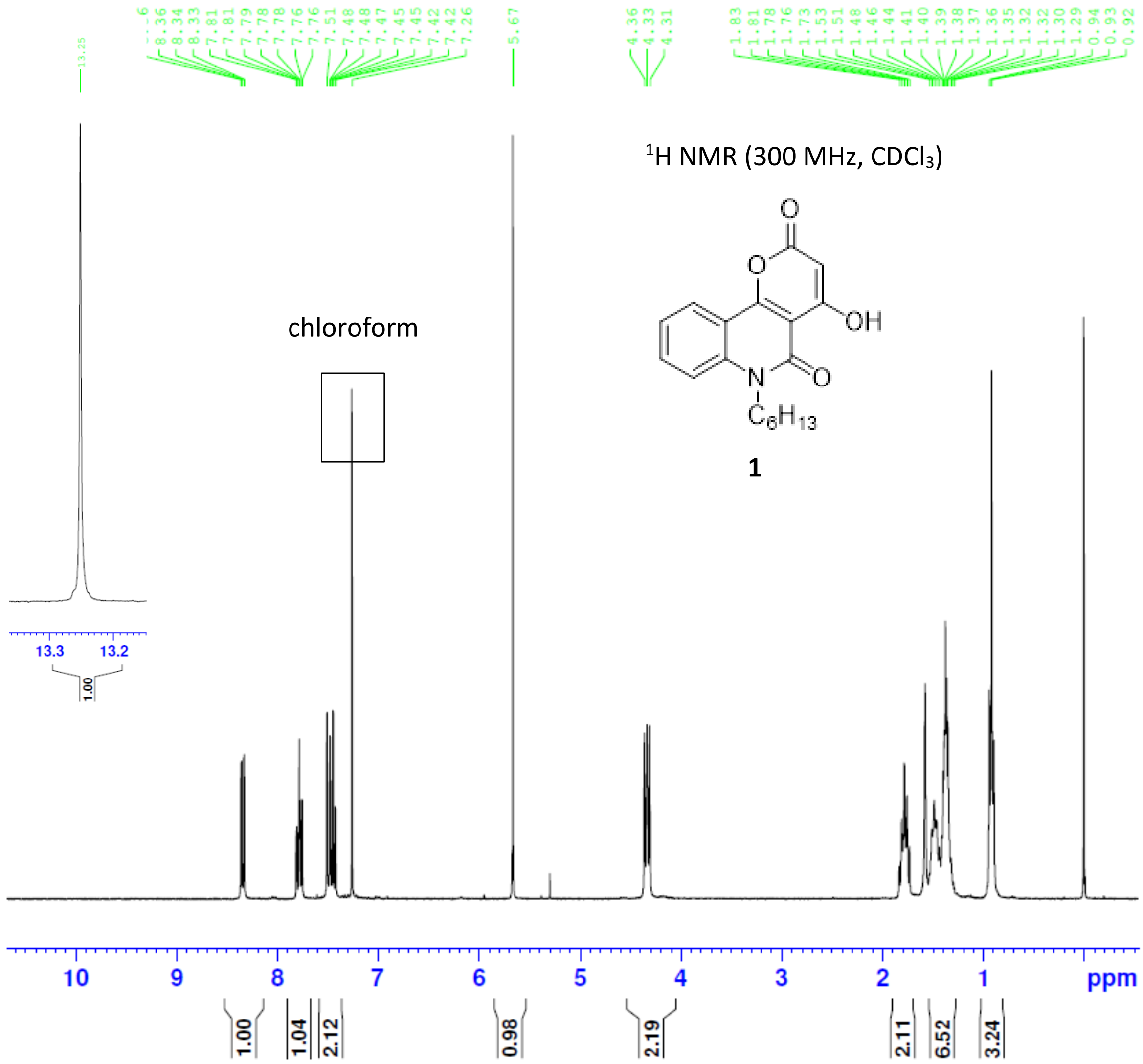

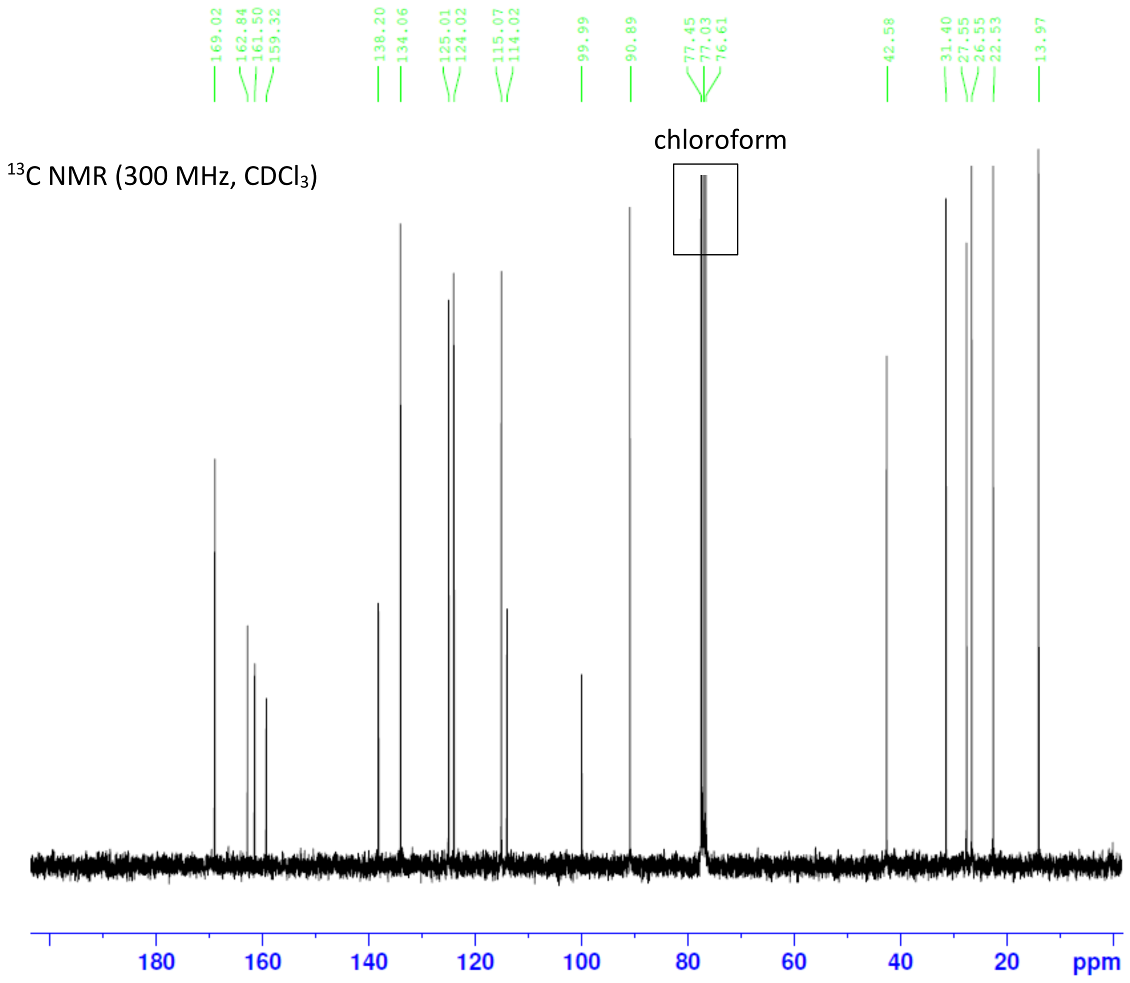
169.02
162.84
161.50
159.32
138.20
134.06
125.01
124.02
115.07
114.02
99.99
90.89
77.45
77.03
76.61
42.58
31.40
27.55
26.55
22.53
13.97
chloroform
13C NMR (300 MHz, CDCl3)
180
160
140
120
100
80
60
40
20
ppm

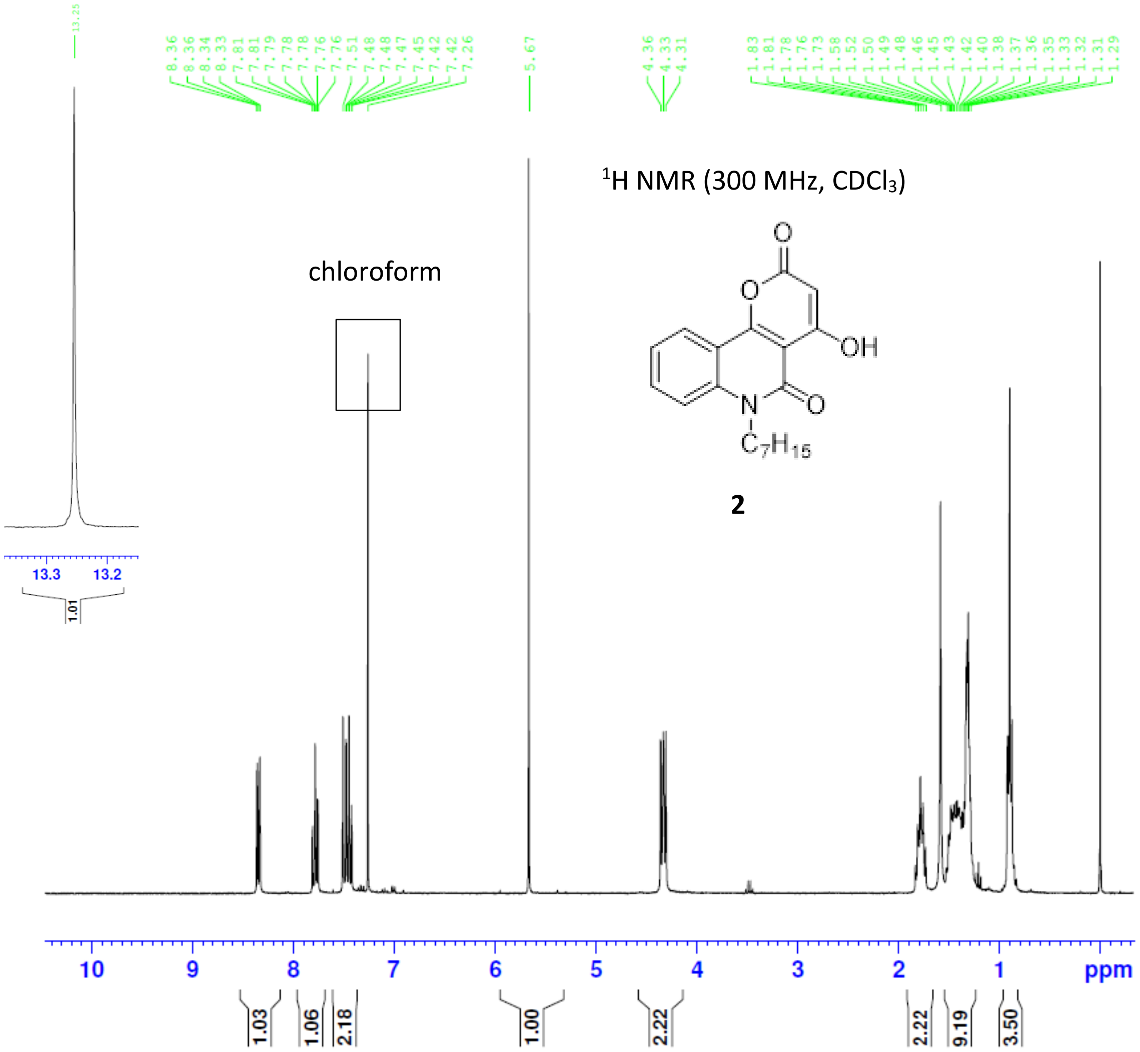
13.25
8.36 8.36 8.34 8.33 7.81 7.81 7.79 7.78 7.78 7.76 7.76 7.51 7.48 7.48 7.47 7.45 7.42 7.42 7.26
5.67
4.36 4.33 4.31
1.83 1.81 1.78 1.76 1.73 1.58 1.52 1.50 1.49 1.48 1.46 1.45 1.43 1.42 1.40 1.38 1.37 1.36 1.35 1.33 1.32 1.31 1.29
1H NMR (300 MHz, CDCl3)
chloroform
O
O
OH
N
O
C7H15
2
13.3 13.2
1.01
10 9 8 7 6 5 4 3 2 1 ppm
1.03 1.06 2.18 1.00 2.22 2.22 9.19 3.50

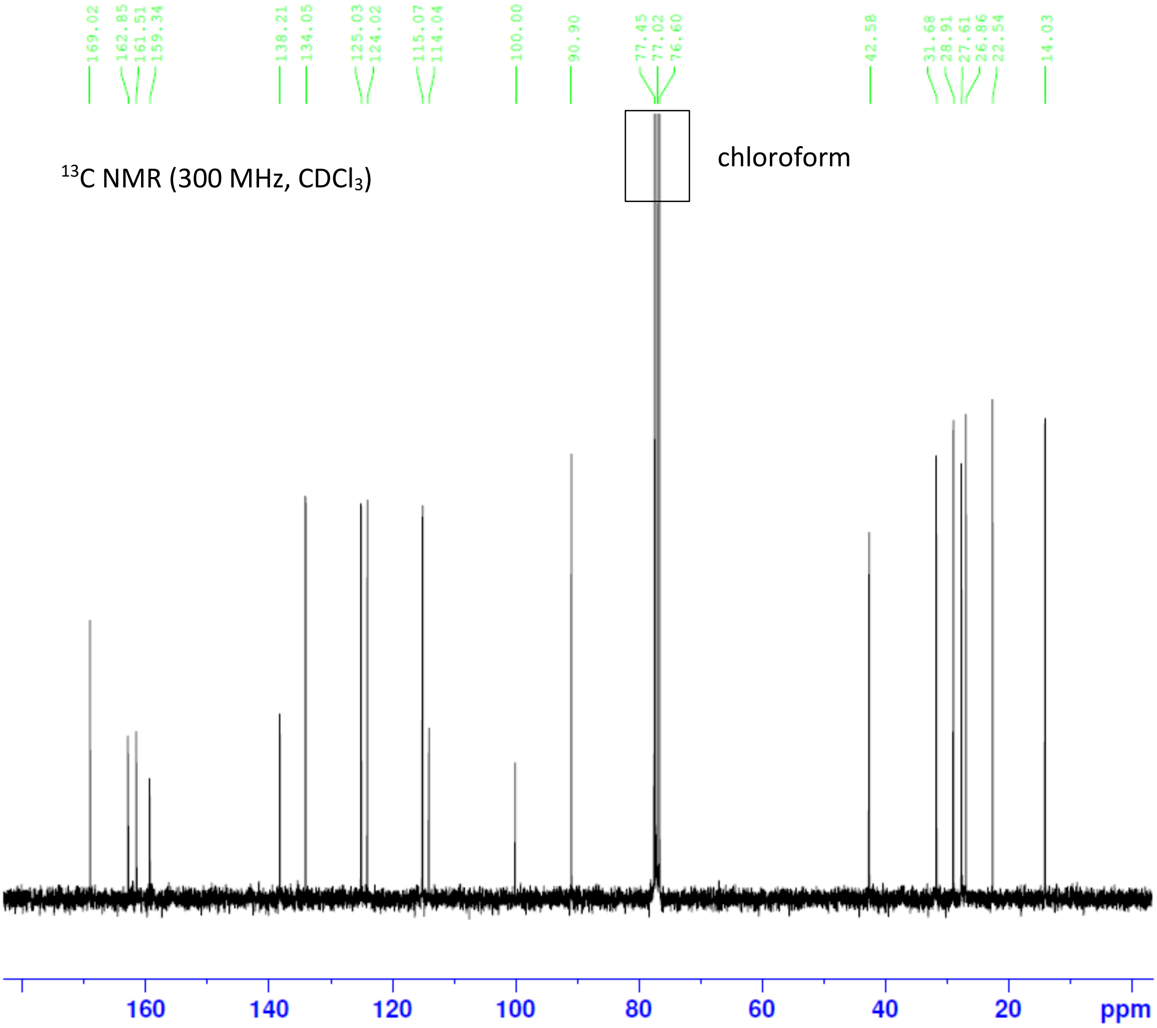
169.02
162.85
161.51
159.34
138.21
134.05
125.03
124.02
115.07
114.04
100.00
90.90
77.45
77.02
76.60
42.58
31.68
28.91
27.61
26.86
22.54
14.03
13C NMR (300 MHz, CDCl3)
chloroform
160
140
120
100
80
60
40
20
ppm

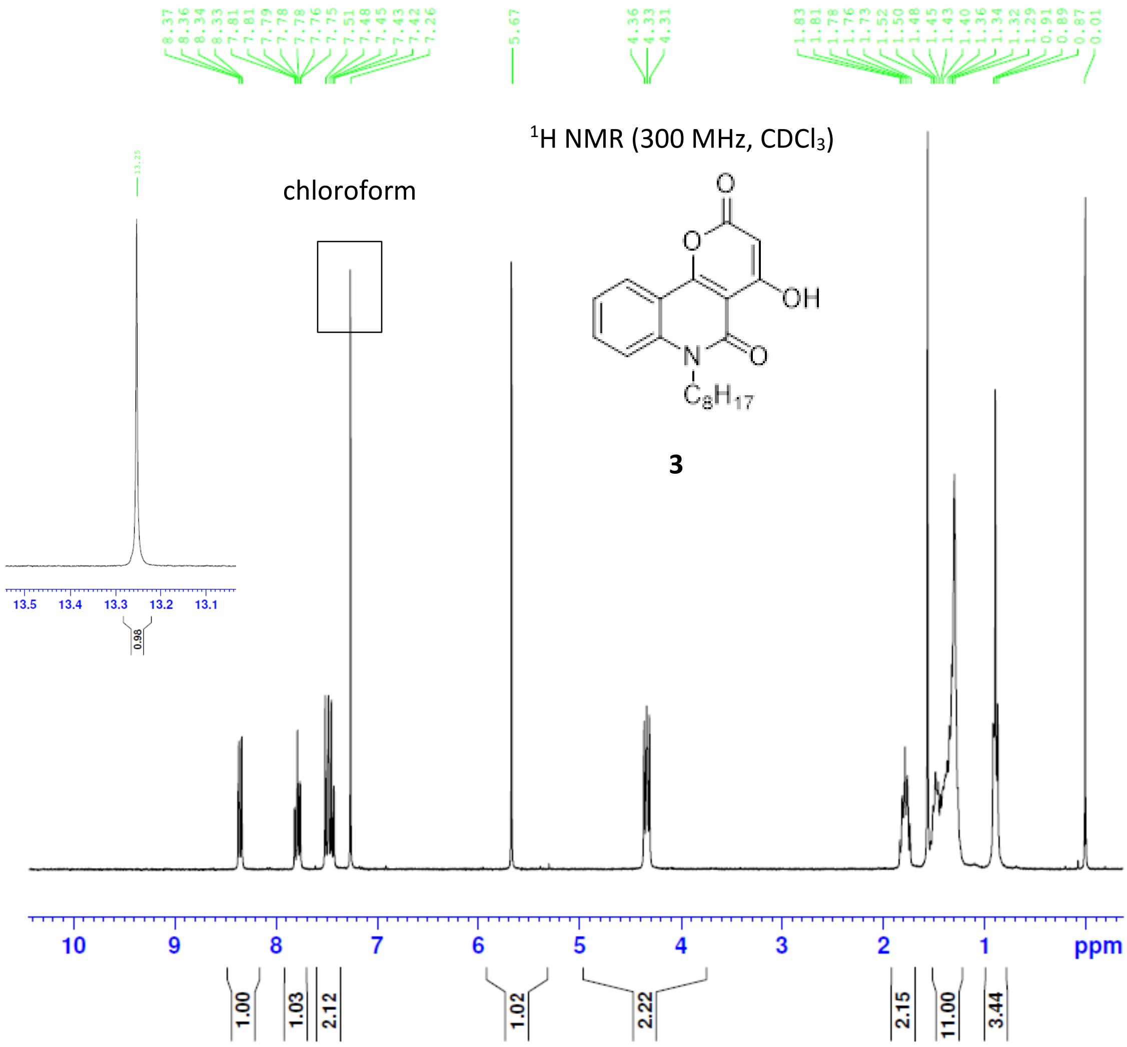

chloroform
^{1}H NMR (300 MHz, CDCl$_3$)
O
OH
N
C$_8$H$_{17}$
3
ppm

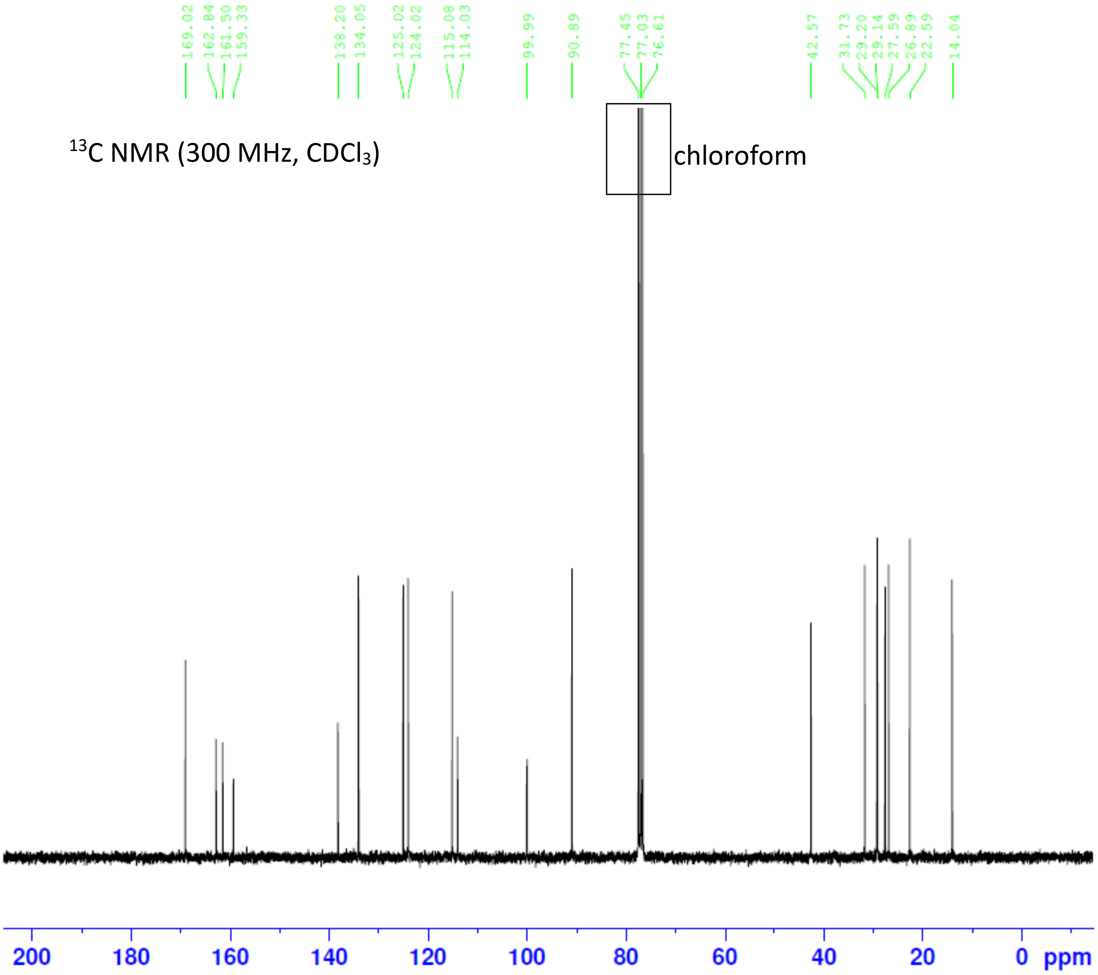
169.02
162.84
161.50
159.33
138.20
134.05
125.02
124.02
115.08
114.03
99.99
90.89
77.45
77.03
76.61
42.57
31.73
29.20
29.14
27.59
26.89
22.59
14.04
13C NMR (300 MHz, CDCl3)
chloroform
200
180
160
140
120
100
80
60
40
20
0
ppm

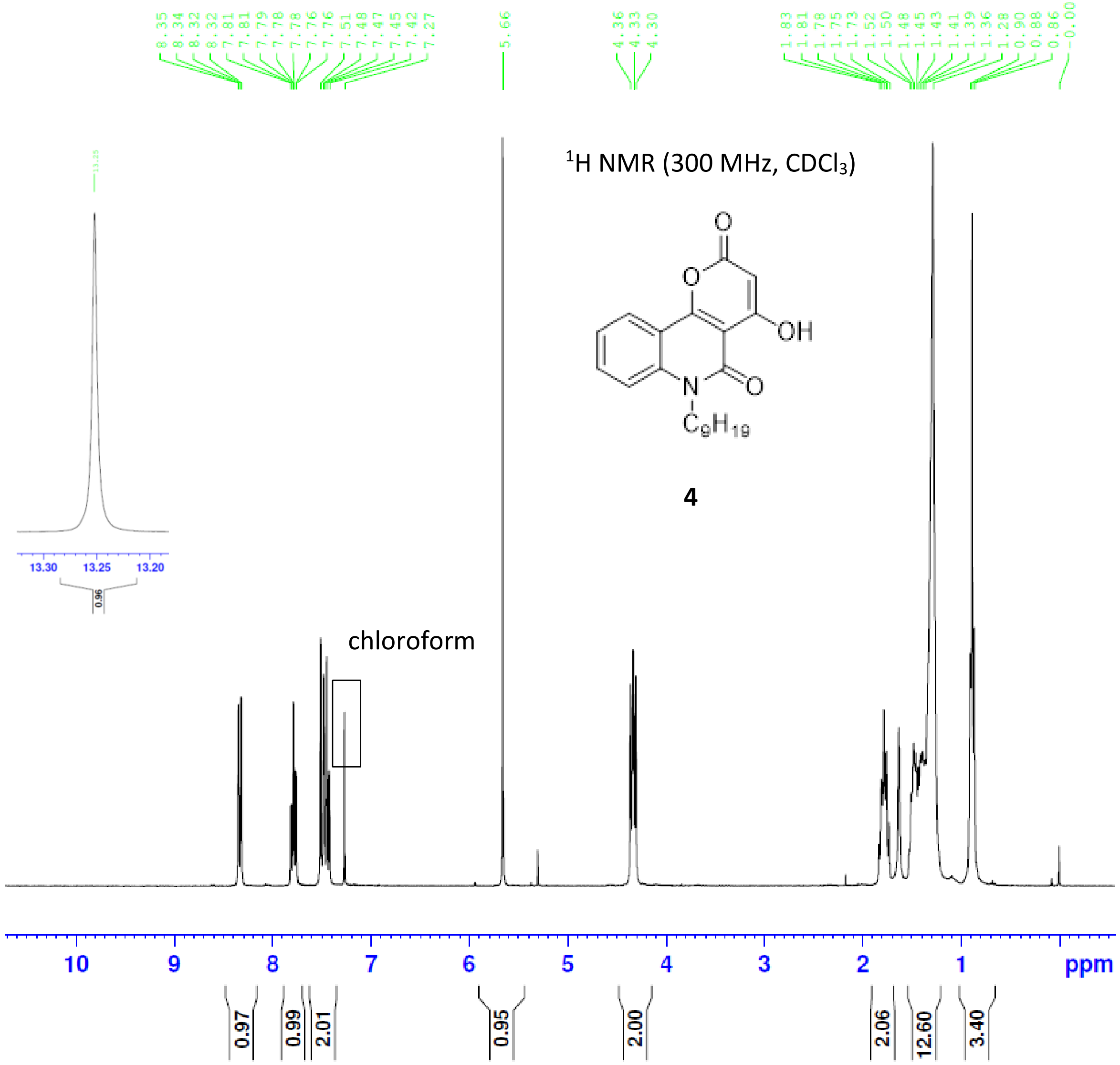

^{1}H NMR (300 MHz, CDCl$_3$)
chloroform
OH
N
O
4
ppm

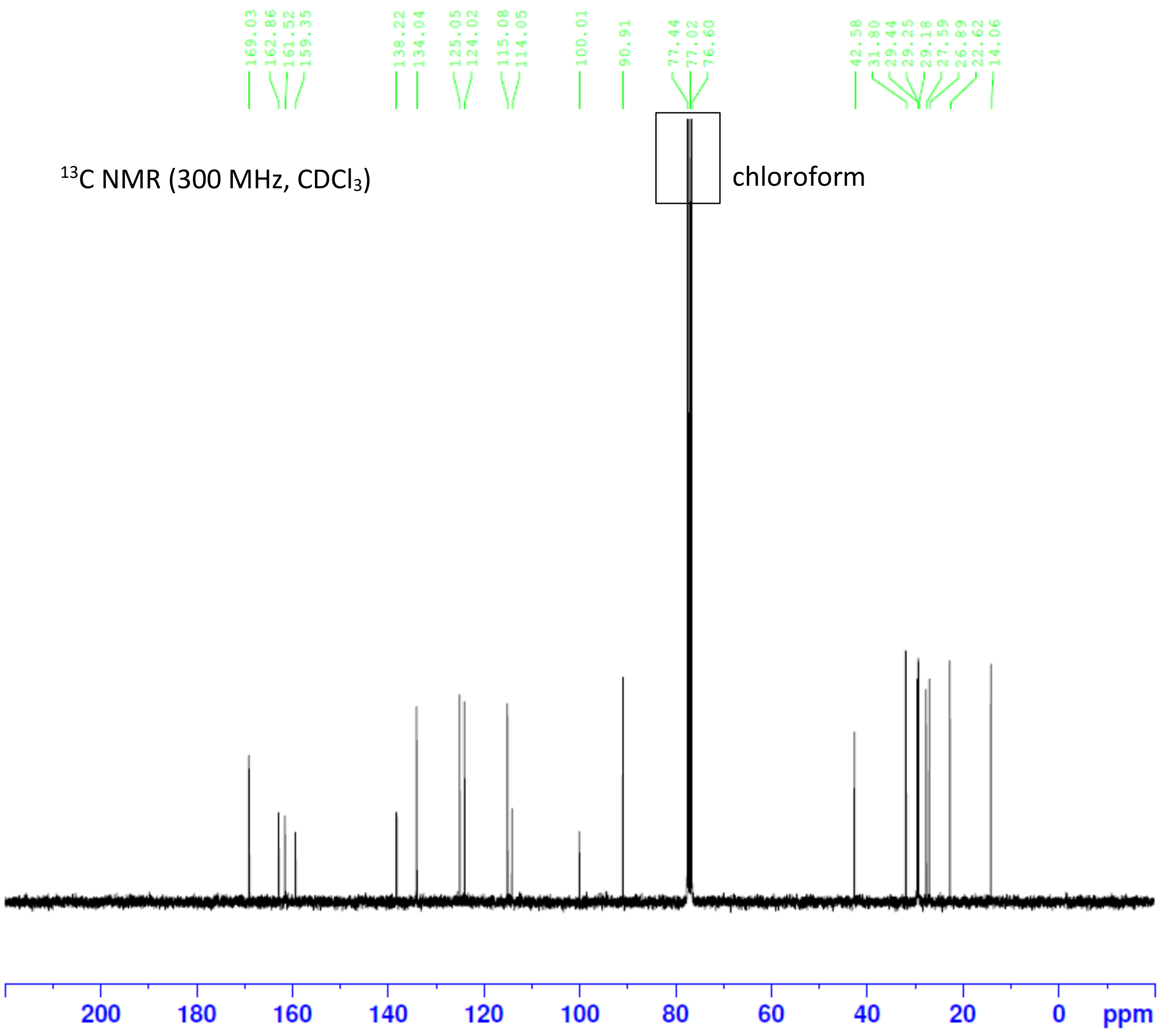
169.03
162.86
161.52
159.35
138.22
134.04
125.05
124.02
115.08
114.05
100.01
90.91
77.44
77.02
76.60
42.58
31.80
29.44
29.25
29.18
27.59
26.89
22.62
14.06
^{13}C NMR (300 MHz, CDCl$_3$)
chloroform
200
180
160
140
120
100
80
60
40
20
0
ppm

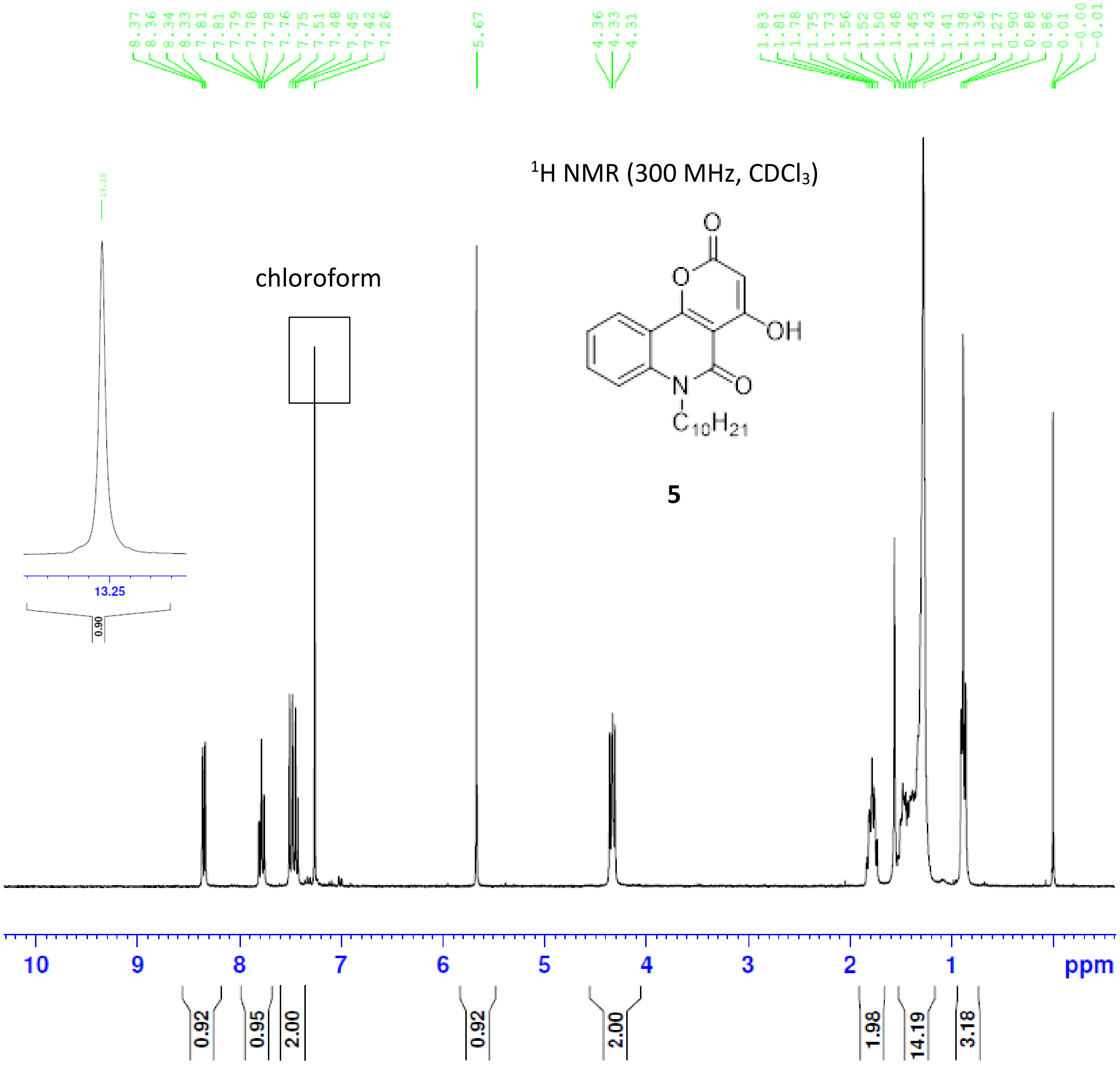

¹H NMR (300 MHz, CDCl₃)
chloroform
OH
5
ppm

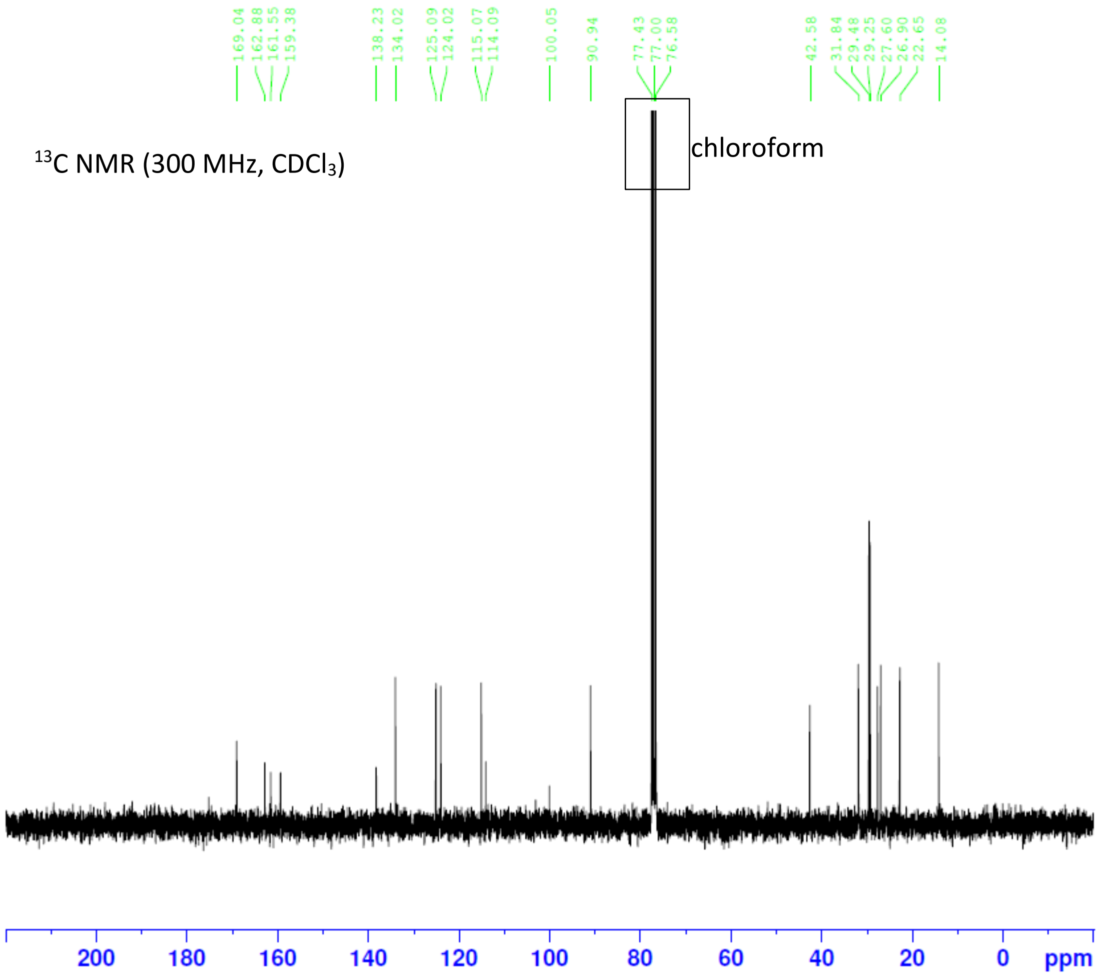

169.04
162.88
161.55
159.38
138.23
134.02
125.09
124.02
115.07
114.09
100.05
90.94
77.43
77.00
76.58
42.58
31.84
29.48
29.25
27.60
26.90
22.65
14.08
¹³C NMR (300 MHz, CDCl₃)
chloroform
200
180
160
140
120
100
80
60
40
20
0
ppm

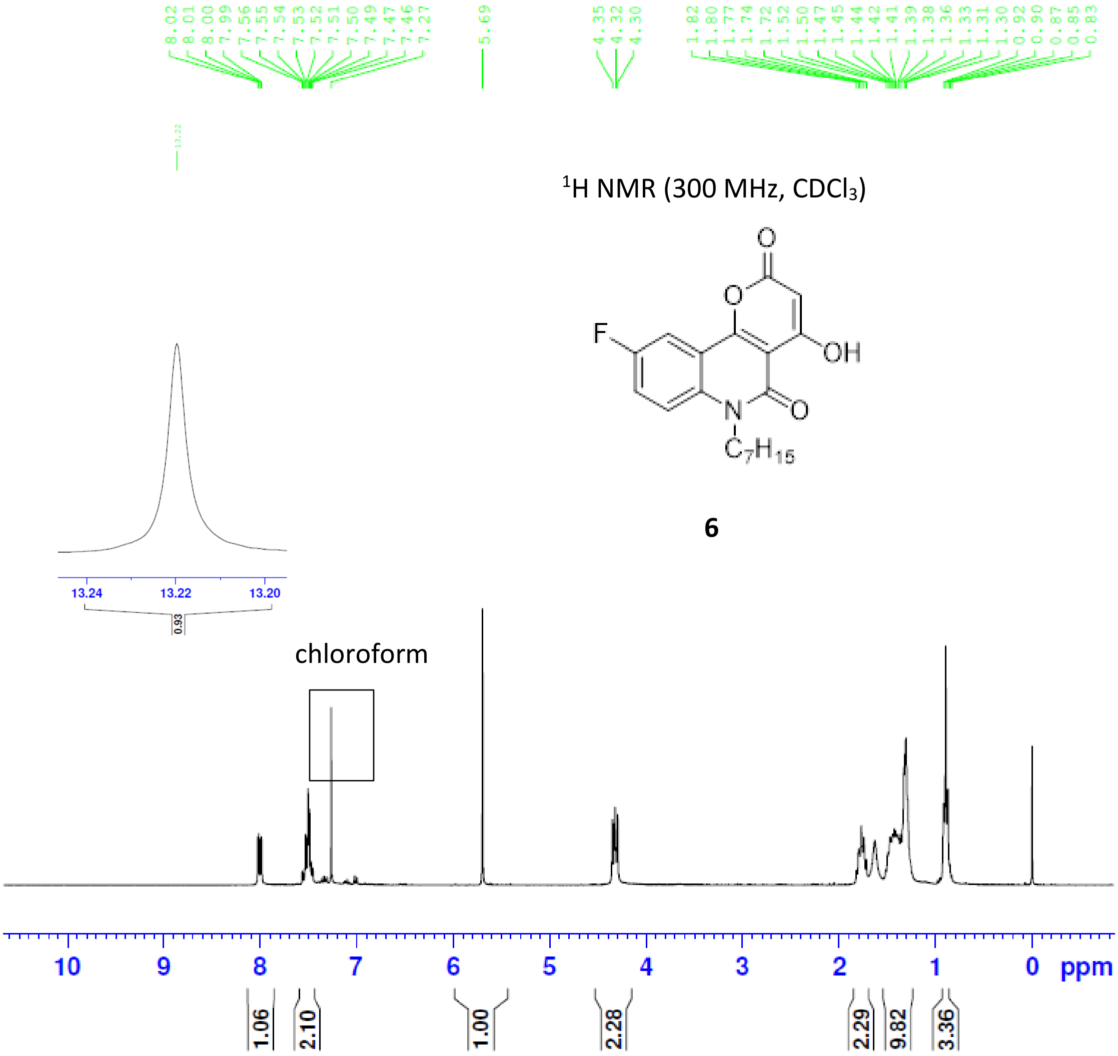

8.02 8.01 8.00 7.99 7.56 7.55 7.54 7.53 7.52 7.51 7.50 7.49 7.47 7.46 7.27
5.69
4.35 4.32 4.30
1.82 1.80 1.77 1.74 1.72 1.52 1.50 1.47 1.45 1.44 1.42 1.41 1.39 1.38 1.36 1.33 1.31 1.30 0.92 0.90 0.87 0.85 0.83
^{1}H NMR (300 MHz, $CDCl_3$)
F
O
OH
N
C_7H_{15}
6
13.24
13.22
13.20
0.93
chloroform
10
9
8
7
6
5
4
3
2
1
0
ppm
1.06
2.10
1.00
2.28
2.29
9.82
3.36

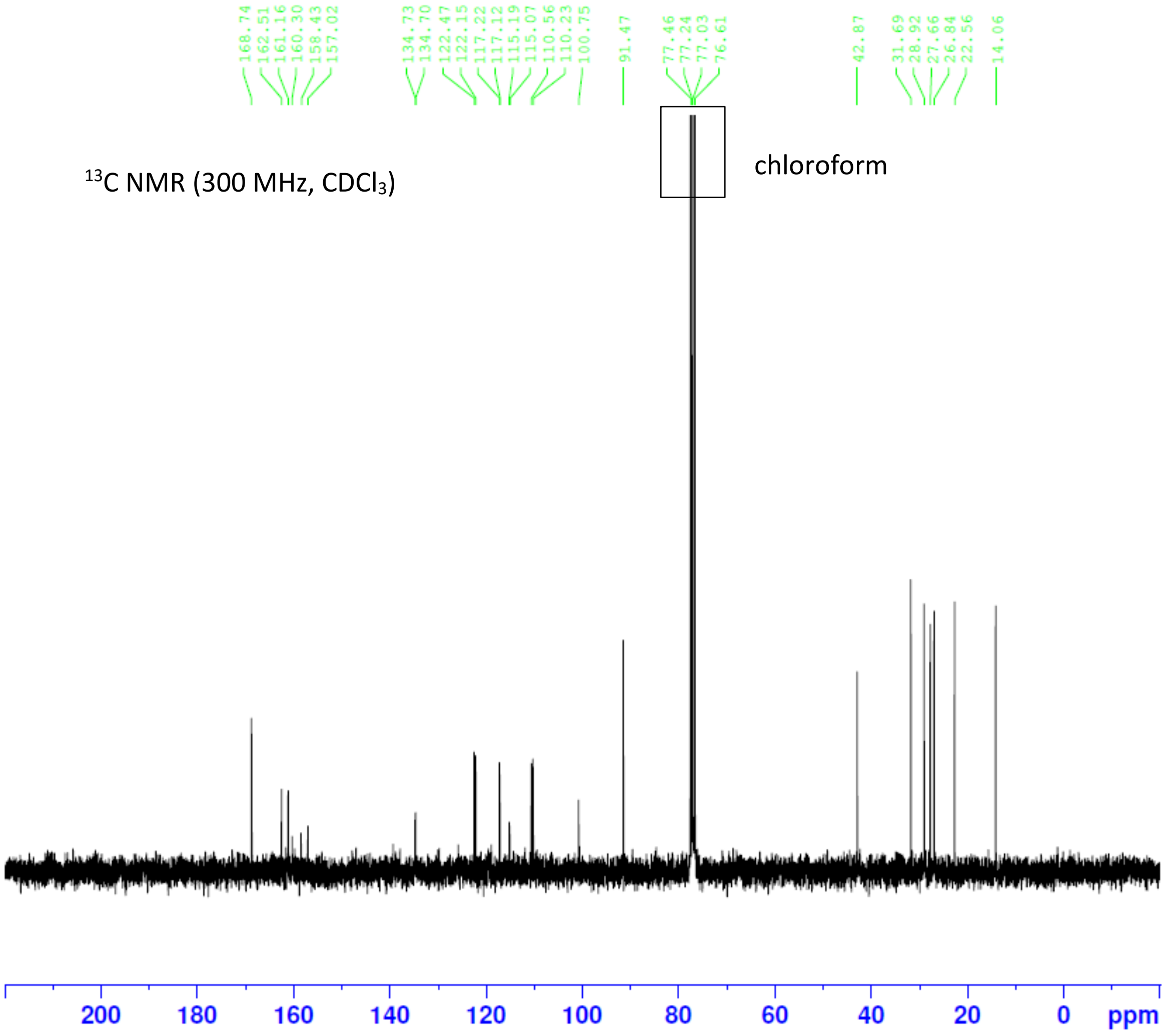
168.74
162.51
161.16
160.30
158.43
157.02
134.73
134.70
122.47
122.15
117.22
117.12
115.19
115.07
110.56
110.23
100.75
91.47
77.46
77.24
77.03
76.61
42.87
31.69
28.92
27.66
26.84
22.56
14.06
13C NMR (300 MHz, CDCl3)
chloroform
200
180
160
140
120
100
80
60
40
20
0
ppm

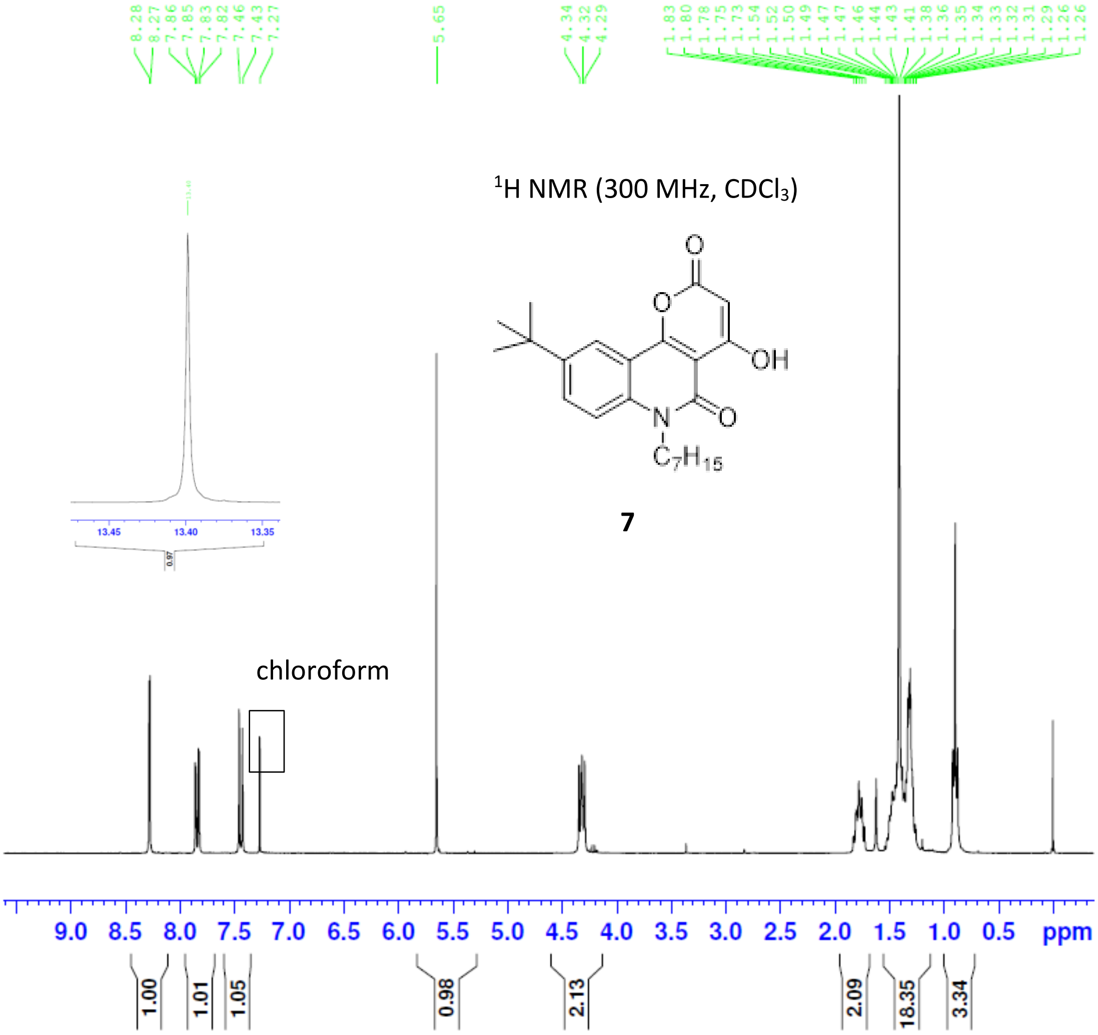

8.28
8.27
7.86
7.85
7.83
7.82
7.46
7.43
7.27
5.65
4.34
4.32
4.29
1.83
1.80
1.78
1.75
1.73
1.54
1.52
1.50
1.49
1.47
1.47
1.46
1.44
1.43
1.41
1.38
1.36
1.35
1.34
1.33
1.32
1.31
1.29
1.26
1.26
^{1}H NMR (300 MHz, $CDCl_3$)
O
O
OH
N
O
C_7H_{15}
13.45
13.40
13.35
0.97
chloroform
9.0 8.5 8.0 7.5 7.0 6.5 6.0 5.5 5.0 4.5 4.0 3.5 3.0 2.5 2.0 1.5 1.0 0.5 ppm
1.00
1.01
1.05
0.98
2.13
2.09
18.35
3.34

7

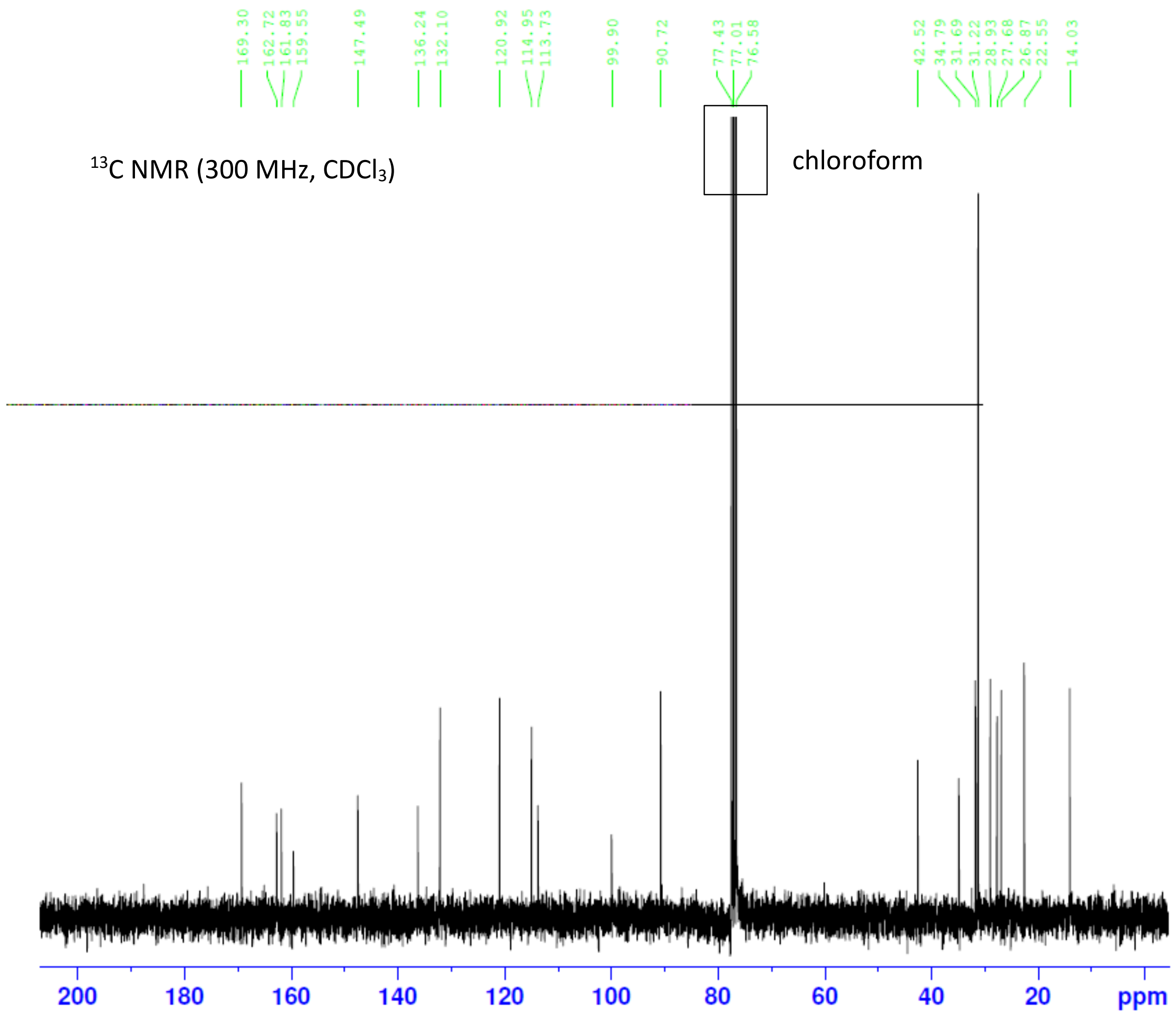
169.30
162.72
161.83
159.55
147.49
136.24
132.10
120.92
114.95
113.73
99.90
90.72
77.43
77.01
76.58
42.52
34.79
31.69
31.22
28.93
27.68
26.87
22.55
14.03
^{13}C NMR (300 MHz, $CDCl_3$)
chloroform
200
180
160
140
120
100
80
60
40
20
ppm

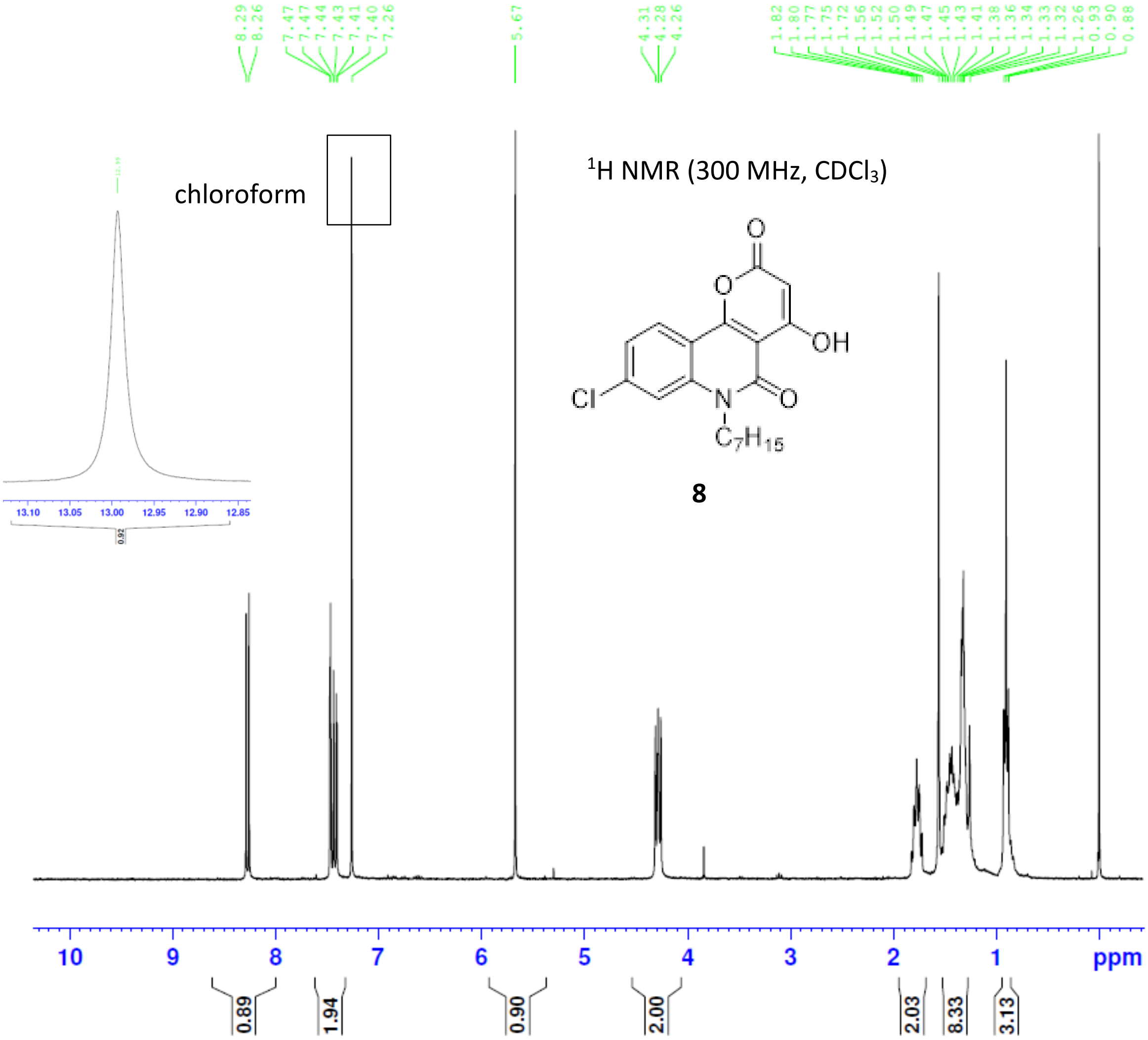
chloroform
^1H NMR (300 MHz, $CDCl_3$)
8
ppm

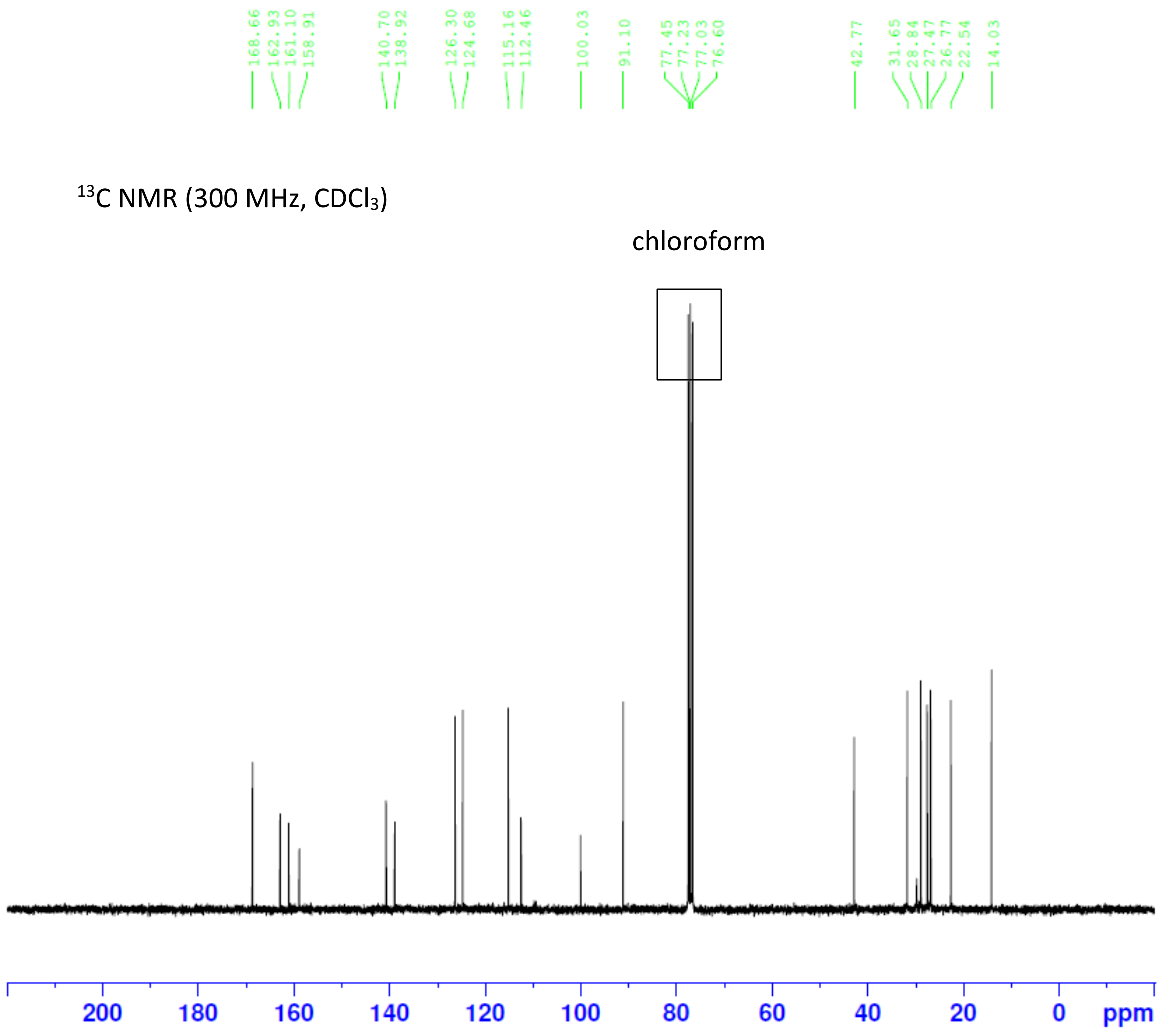
168.66
162.93
161.10
158.91
140.70
138.92
126.30
124.68
115.16
112.46
100.03
91.10
77.45
77.23
77.03
76.60
42.77
31.65
28.84
27.47
26.77
22.54
14.03
^{13}C NMR (300 MHz, $CDCl_3$)
chloroform
200
180
160
140
120
100
80
60
40
20
0
ppm

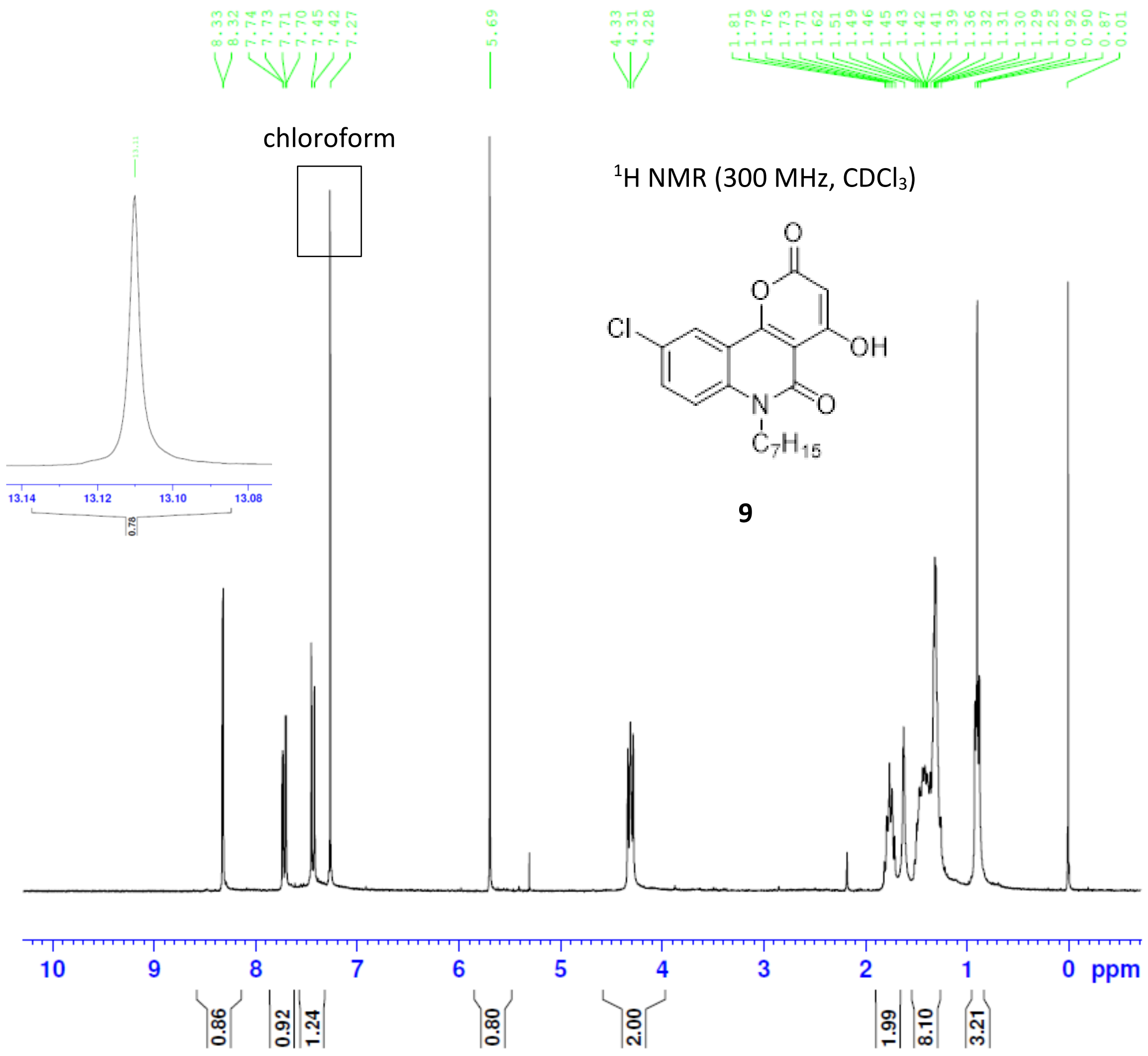

8.33
8.32
7.74
7.73
7.71
7.70
7.45
7.42
7.27
5.69
4.33
4.31
4.28
1.81
1.79
1.76
1.73
1.71
1.62
1.51
1.49
1.46
1.45
1.43
1.42
1.41
1.39
1.36
1.32
1.31
1.30
1.29
1.25
0.92
0.90
0.87
0.01
chloroform
^{1}H NMR (300 MHz, $CDCl_3$)
Cl
O
OH
N
C_7H_{15}
9
13.14
13.12
13.10
13.08
0.78
10
9
8
7
6
5
4
3
2
1
0
ppm
0.86
0.92
1.24
0.80
2.00
1.99
8.10
3.21

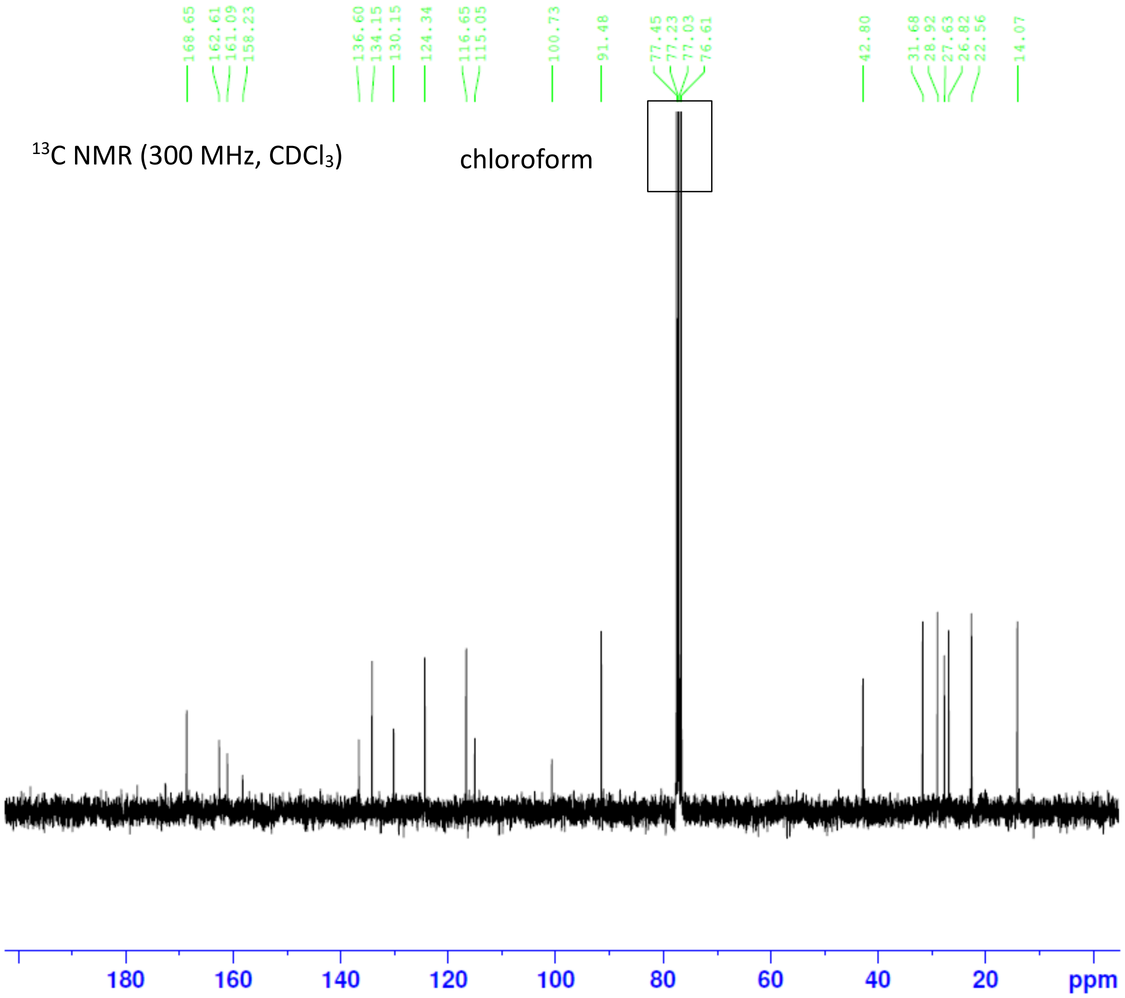
168.65
162.61
161.09
158.23
136.60
134.15
130.15
124.34
116.65
115.05
100.73
91.48
77.45
77.23
77.03
76.61
42.80
31.68
28.92
27.63
26.82
22.56
14.07
13C NMR (300 MHz, CDCl3)
chloroform
180
160
140
120
100
80
60
40
20
ppm

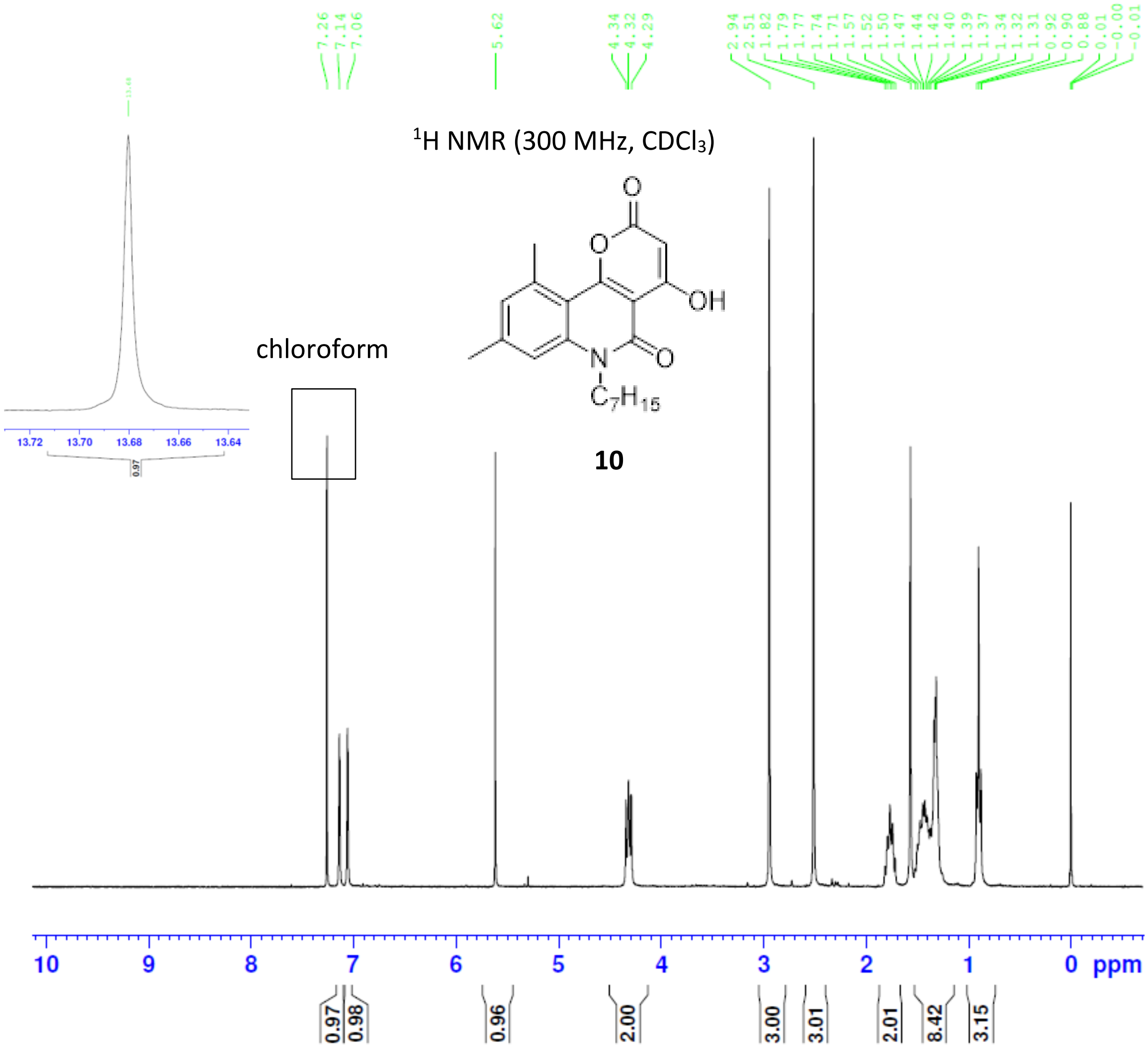

7.26
7.14
7.06
5.62
4.34
4.32
4.29
2.94
2.51
1.82
1.79
1.77
1.74
1.71
1.57
1.52
1.50
1.47
1.44
1.42
1.40
1.39
1.37
1.34
1.32
1.31
0.92
0.90
0.88
0.01
-0.00
-0.01
^{1}H NMR (300 MHz, CDCl$_3$)
OH
O
N
C_7H_{15}
10
chloroform
13.72
13.70
13.68
13.66
13.64
0.97
10
9
8
7
6
5
4
3
2
1
0
ppm
0.97
0.98
0.96
2.00
3.00
3.01
2.01
8.42
3.15

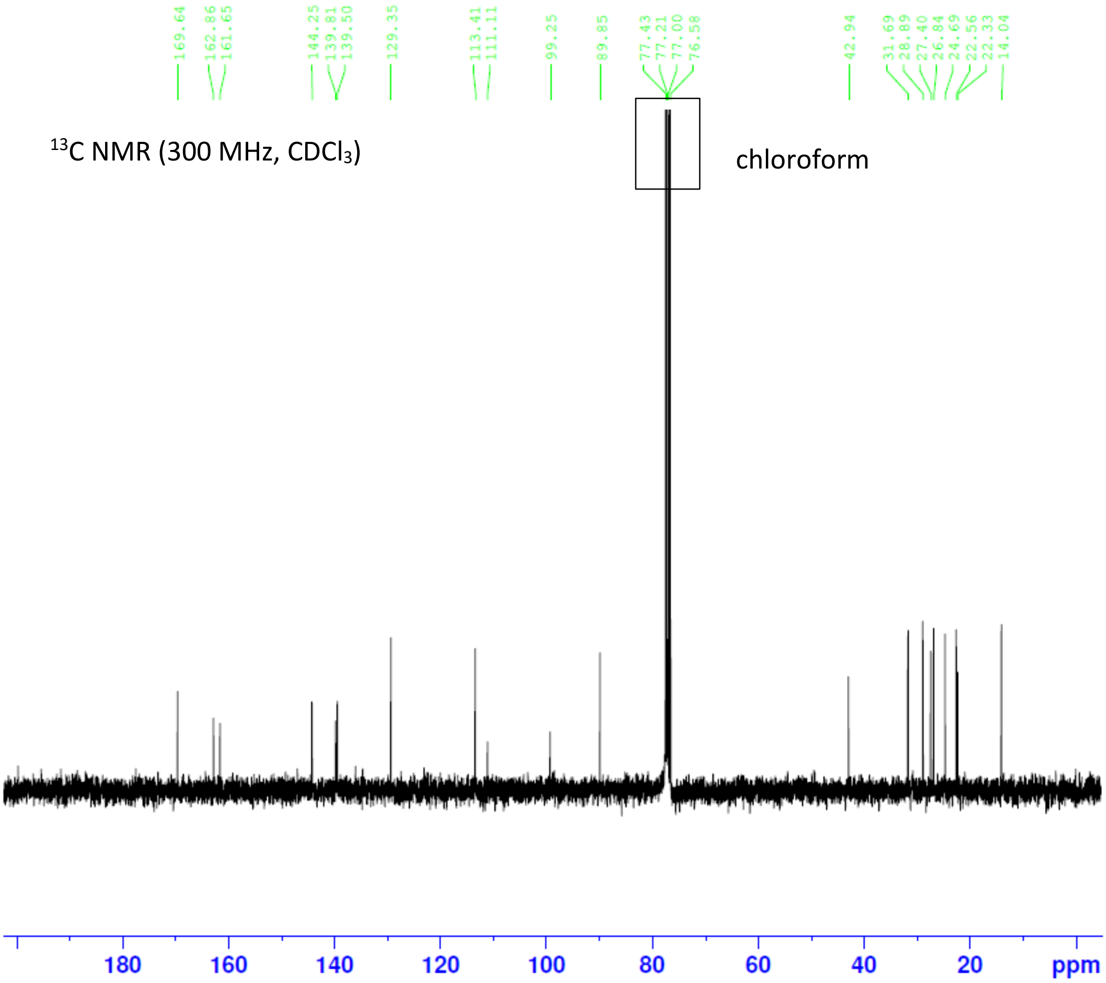
169.64
162.86
161.65
144.25
139.81
139.50
129.35
113.41
111.11
99.25
89.85
77.43
77.21
77.00
76.58
42.94
31.69
28.89
27.40
26.84
24.69
22.56
22.33
14.04
^{13}C NMR (300 MHz, $CDCl_3$)
chloroform
180
160
140
120
100
80
60
40
20
ppm

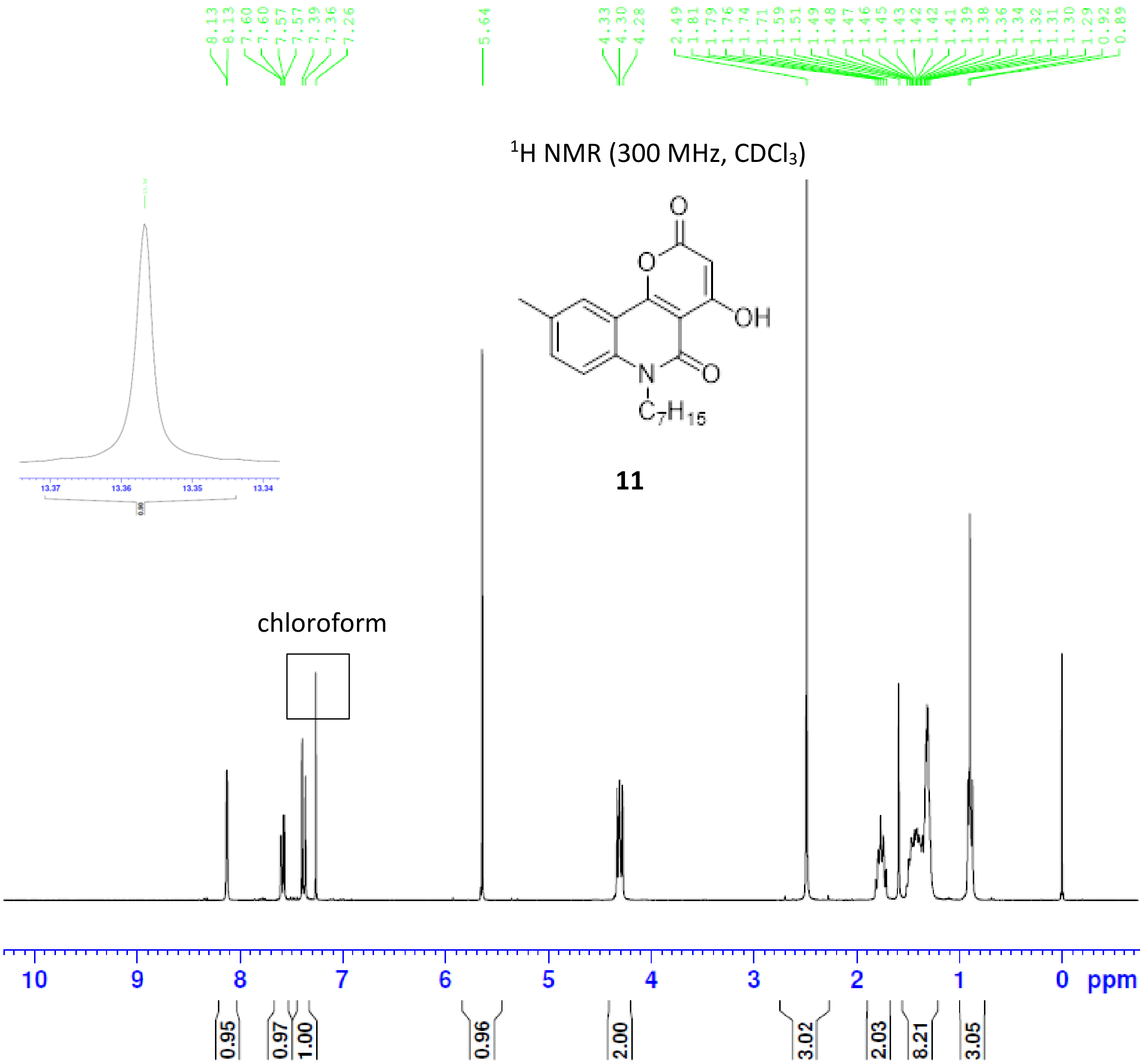

^1H NMR (300 MHz, $CDCl_3$)

11

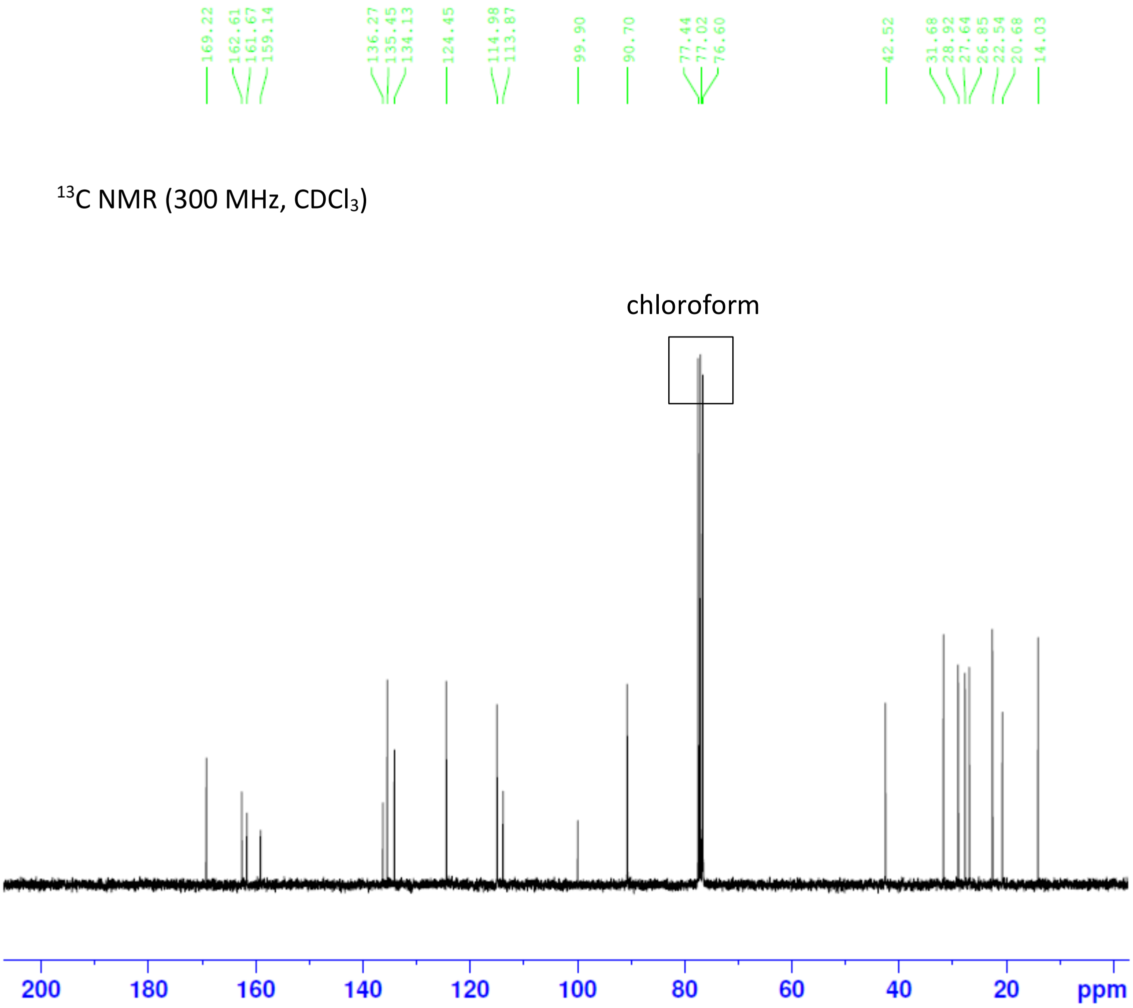
169.22
162.61
161.67
159.14
136.27
135.45
134.13
124.45
114.98
113.87
99.90
90.70
77.44
77.02
76.60
42.52
31.68
28.92
27.64
26.85
22.54
20.68
14.03
13C NMR (300 MHz, CDCl3)
chloroform
200
180
160
140
120
100
80
60
40
20
ppm

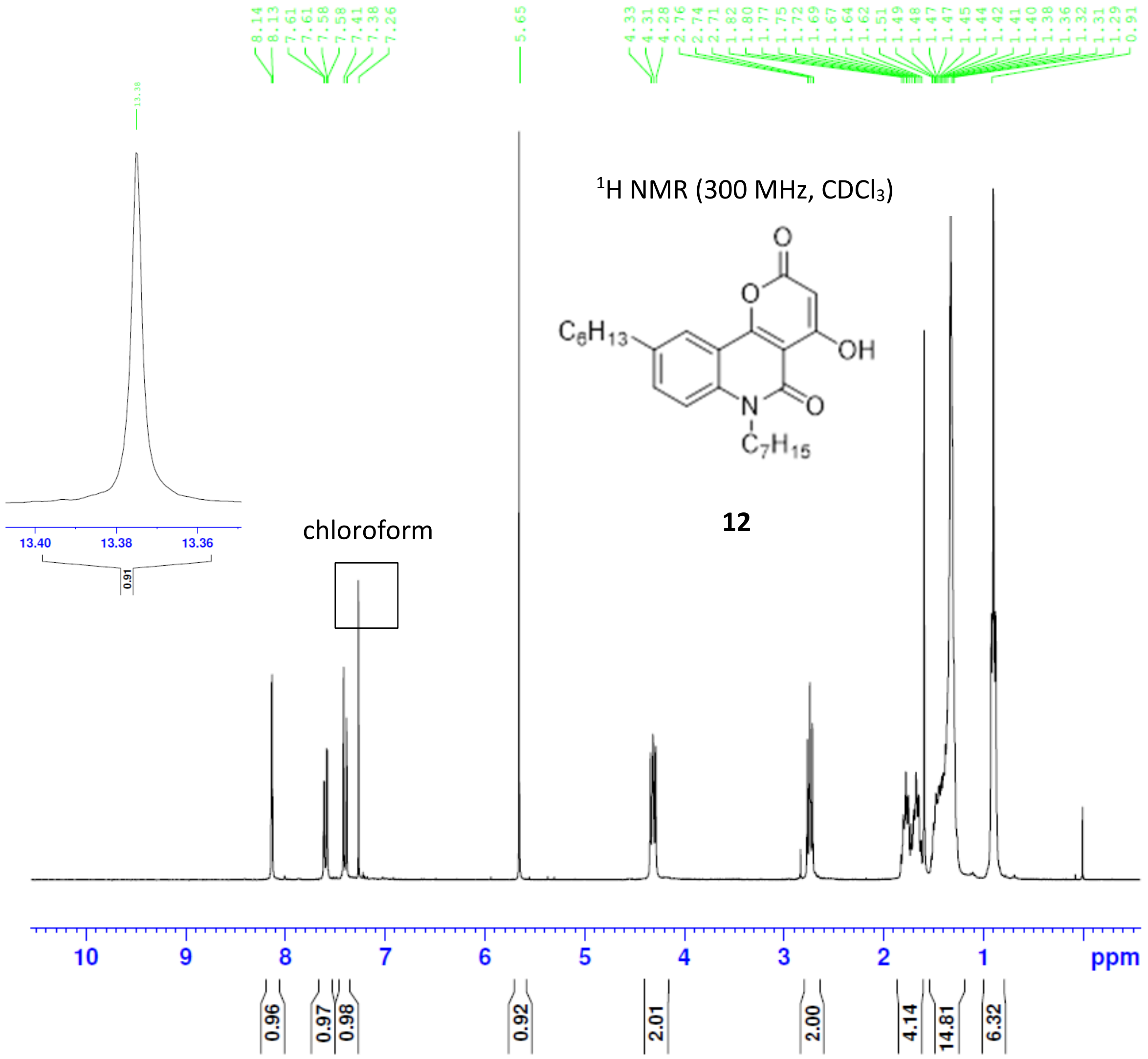
8.14 8.13 7.61 7.61 7.58 7.58 7.41 7.38 7.26
5.65
4.33 4.31 4.28 2.76 2.74 2.71 1.82 1.80 1.77 1.75 1.72 1.69 1.67 1.64 1.62 1.51 1.49 1.48 1.47 1.47 1.45 1.44 1.42 1.41 1.40 1.38 1.36 1.32 1.31 1.29 0.91
13.38
^{1}H NMR (300 MHz, CDCl$_3$)
C_6H_{13}
O
O
OH
N
O
C_7H_{15}
12
13.40
13.38
13.36
0.91
chloroform
10
9
8
7
6
5
4
3
2
1
ppm
0.96
0.97
0.98
0.92
2.01
2.00
4.14
14.81
6.32

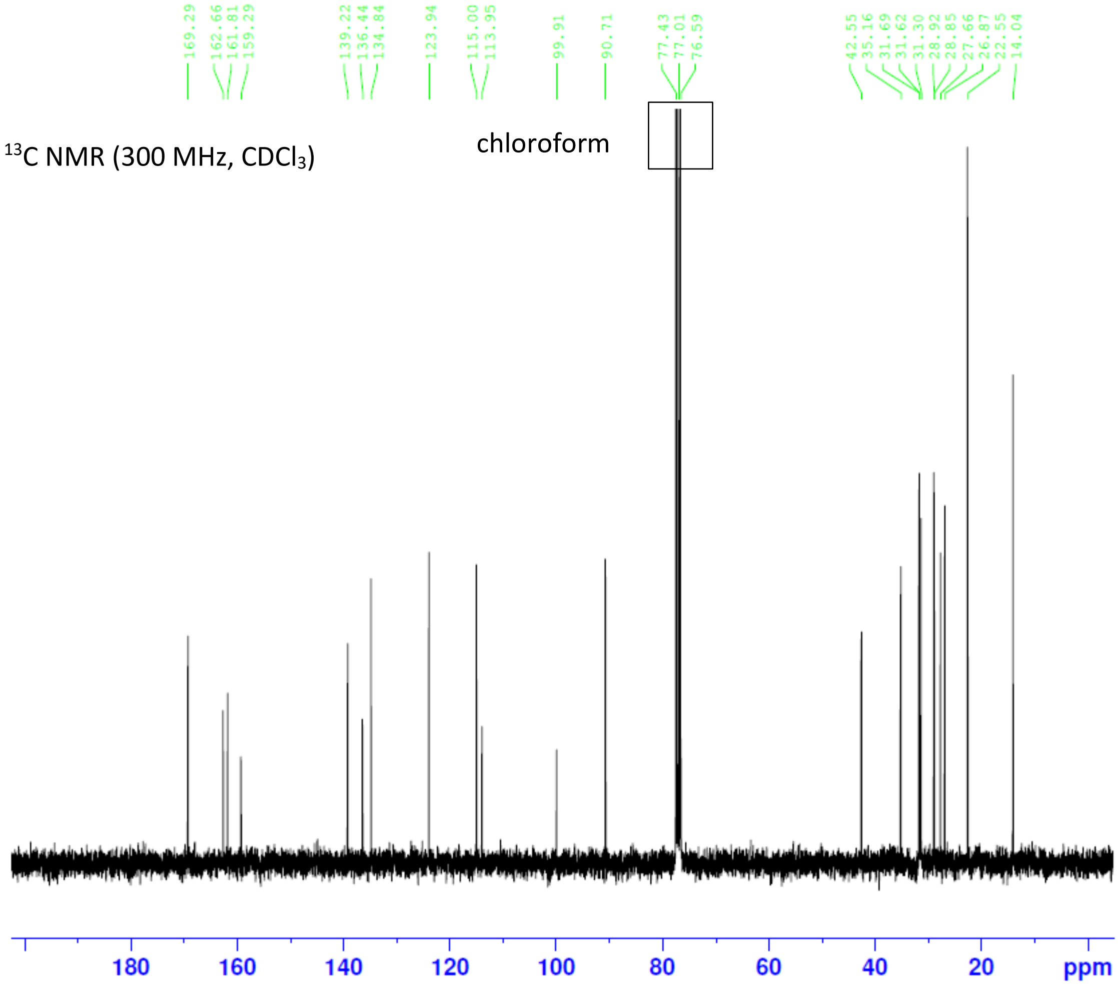
169.29
162.66
161.81
159.29
139.22
136.44
134.84
123.94
115.00
113.95
99.91
90.71
77.43
77.01
76.59
42.55
35.16
31.69
31.62
31.30
28.92
28.85
27.66
26.87
22.55
14.04
^{13}C NMR (300 MHz, $CDCl_3$)
chloroform
180
160
140
120
100
80
60
40
20
ppm

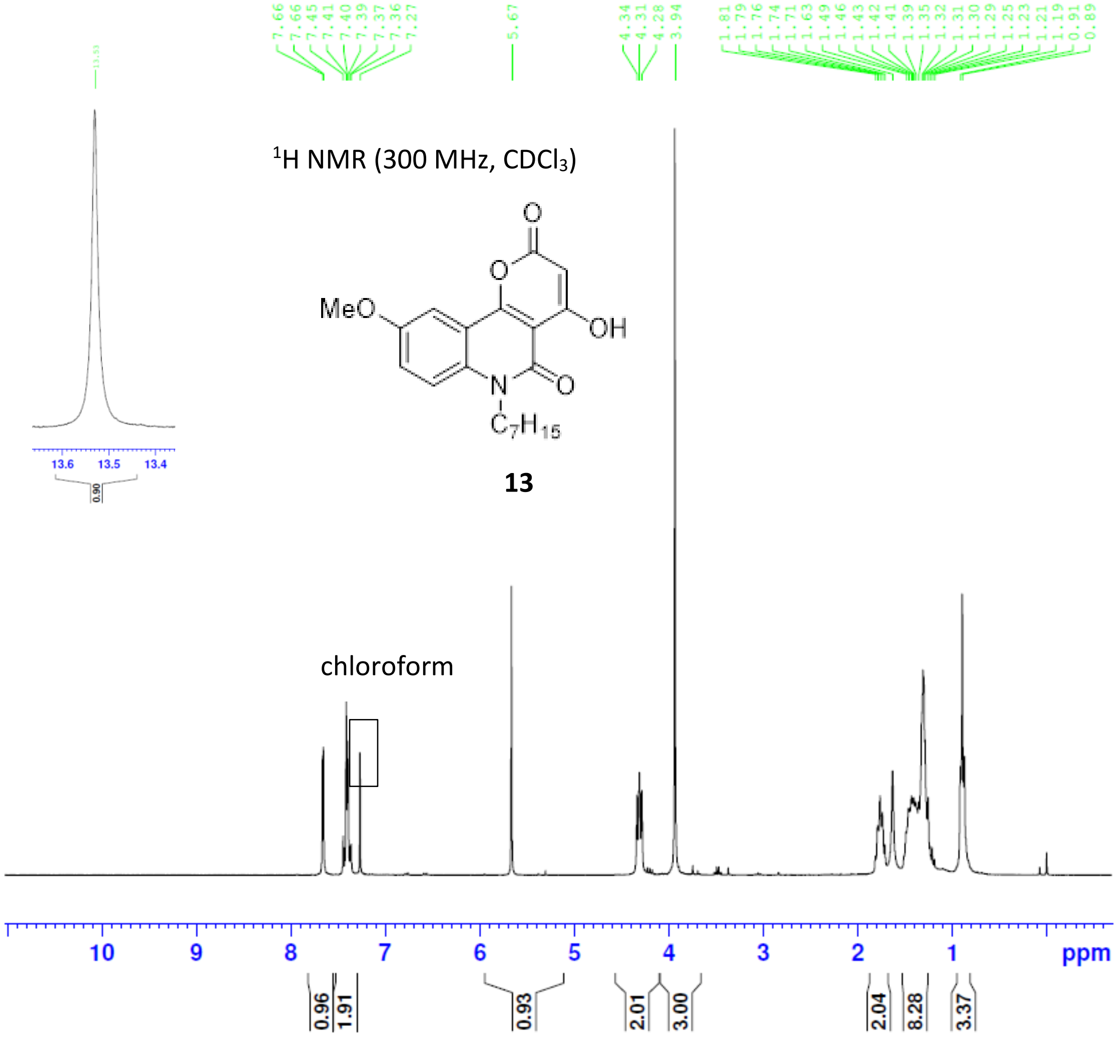

^{1}H NMR (300 MHz, CDCl$_3$)
MeO
O
O
OH
N
O
C$_7$H$_{15}$
13
chloroform
ppm

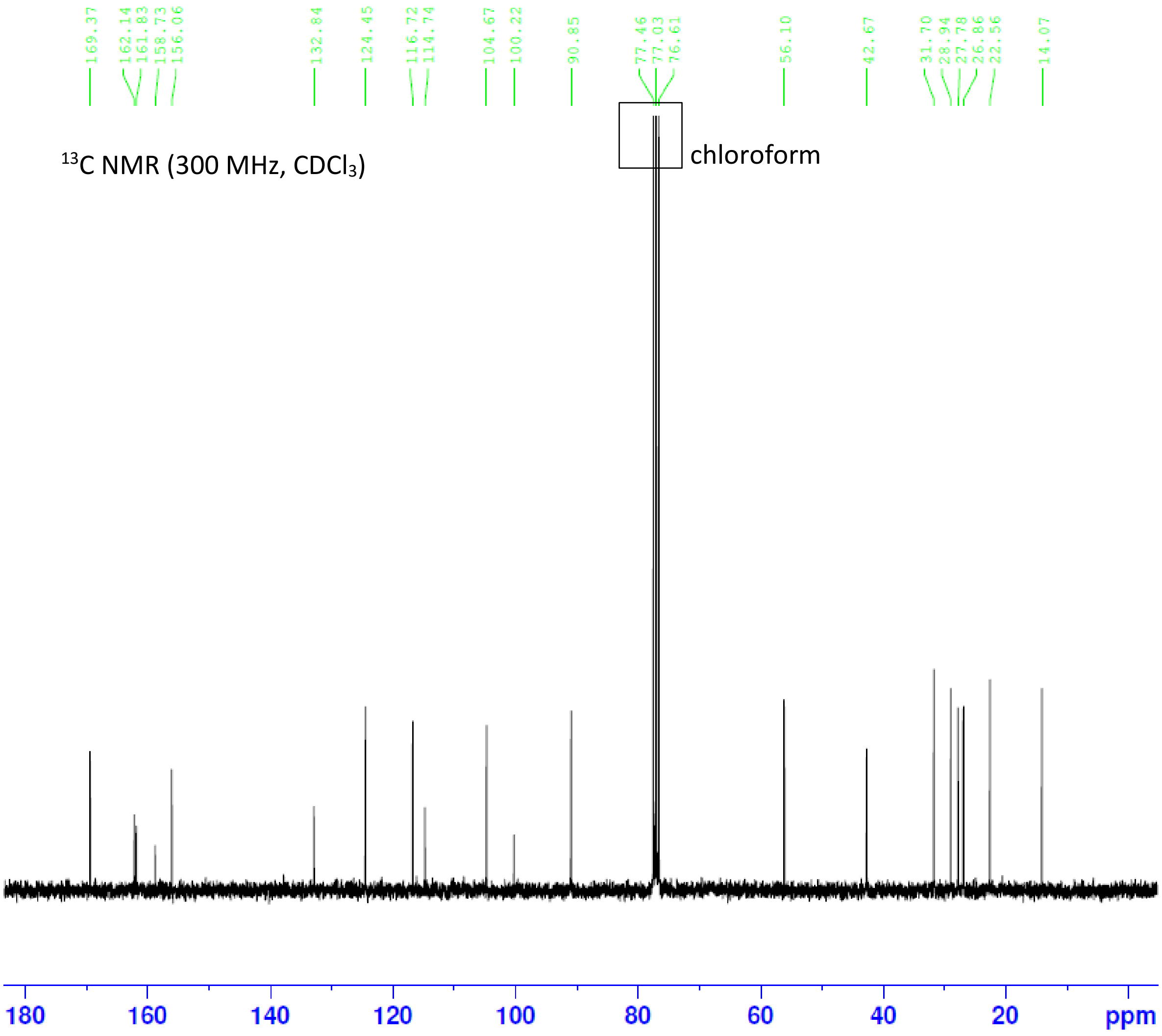
169.37
162.14
161.83
158.73
156.06
132.84
124.45
116.72
114.74
104.67
100.22
90.85
77.46
77.03
76.61
56.10
42.67
31.70
28.94
27.78
26.86
22.56
14.07
^{13}C NMR (300 MHz, $CDCl_3$)
chloroform
180
160
140
120
100
80
60
40
20
ppm

^{1}H NMR (300 MHz, DMSO-d$_6$)

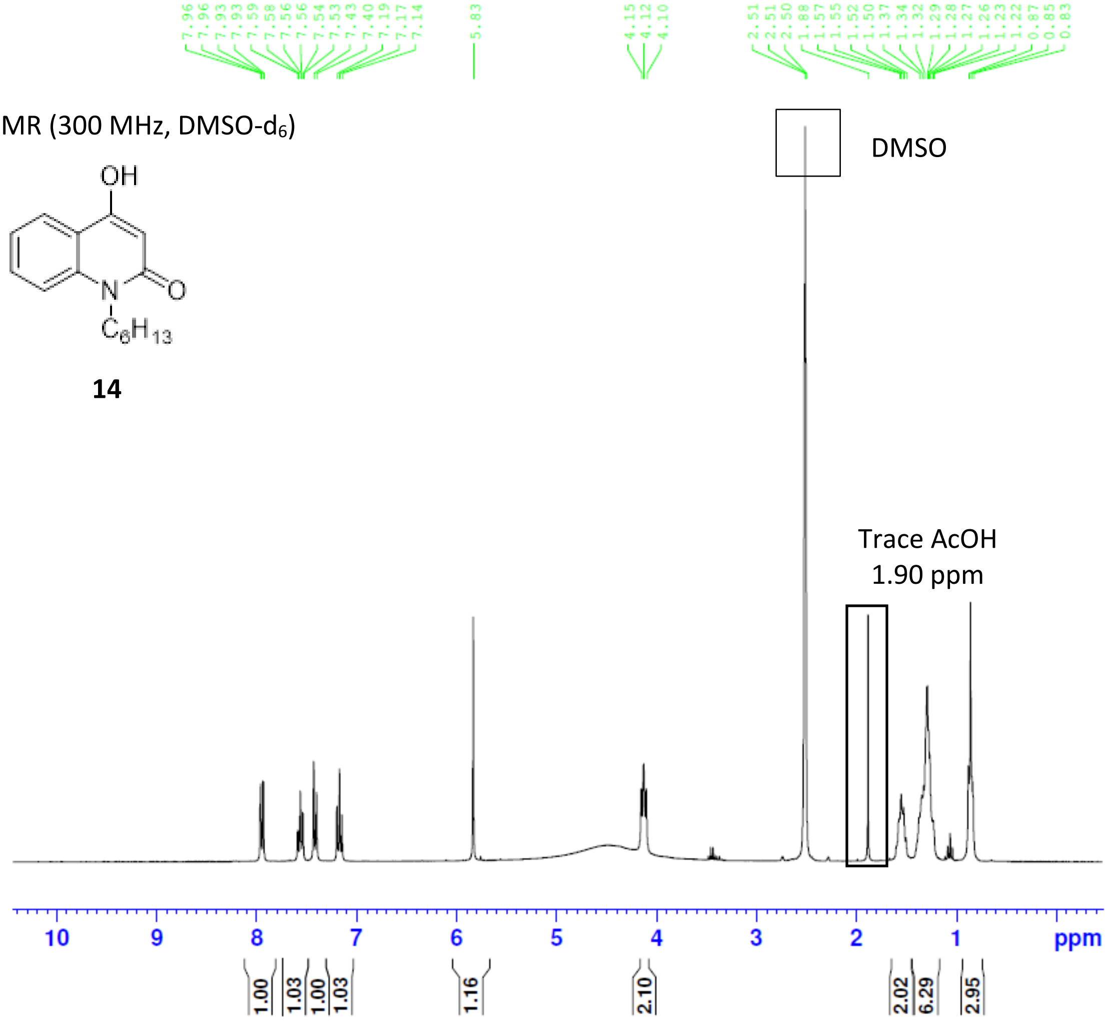

14

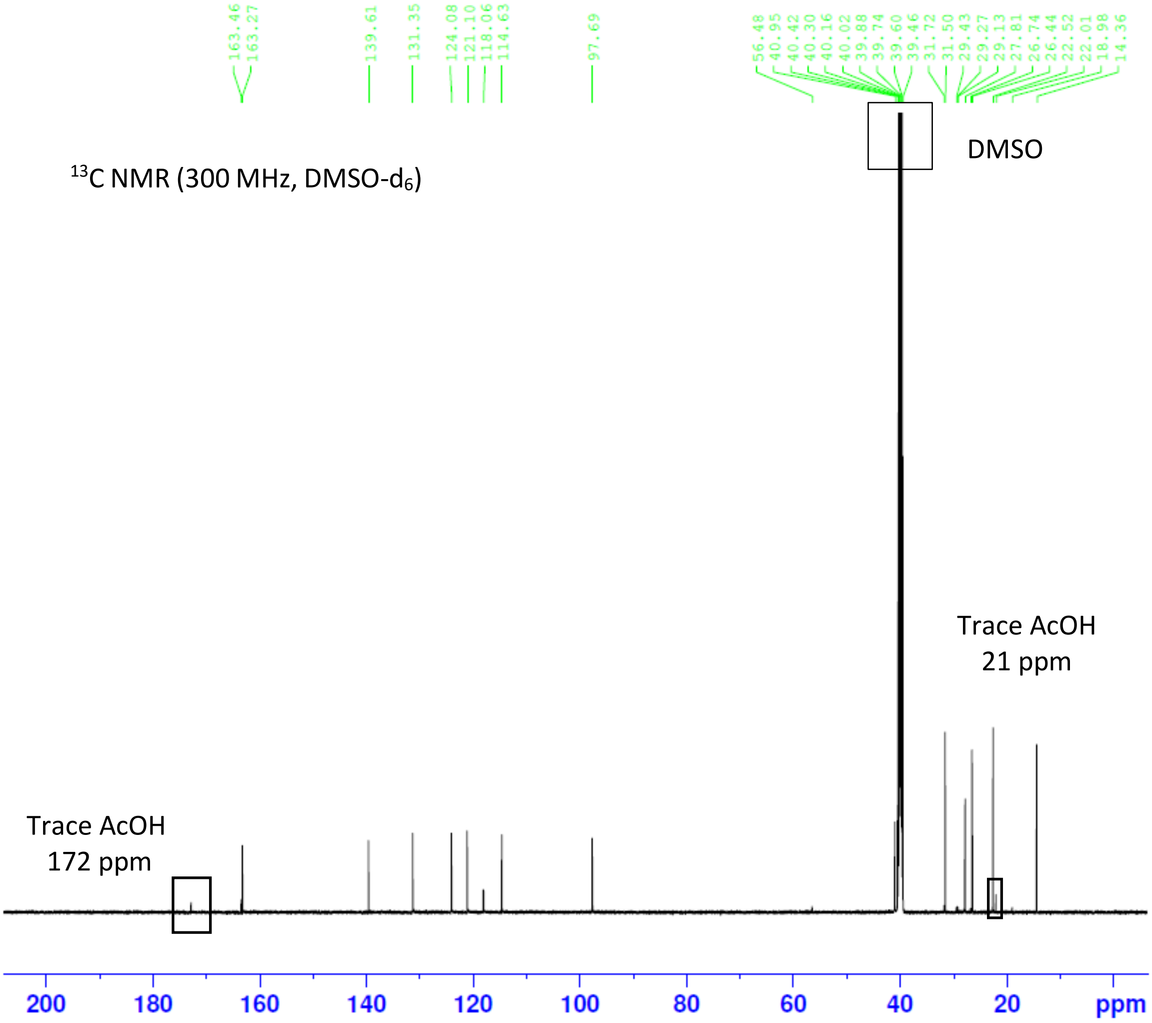
163.46
163.27
139.61
131.35
124.08
121.10
118.06
114.63
97.69
56.48
40.95
40.42
40.30
40.16
40.02
39.88
39.74
39.60
39.46
31.72
31.50
29.43
29.27
29.13
27.81
26.74
26.44
22.52
22.01
18.98
14.36
^{13}C NMR (300 MHz, DMSO-d$_6$)
DMSO
Trace AcOH
21 ppm
Trace AcOH
172 ppm
200
180
160
140
120
100
80
60
40
20
ppm

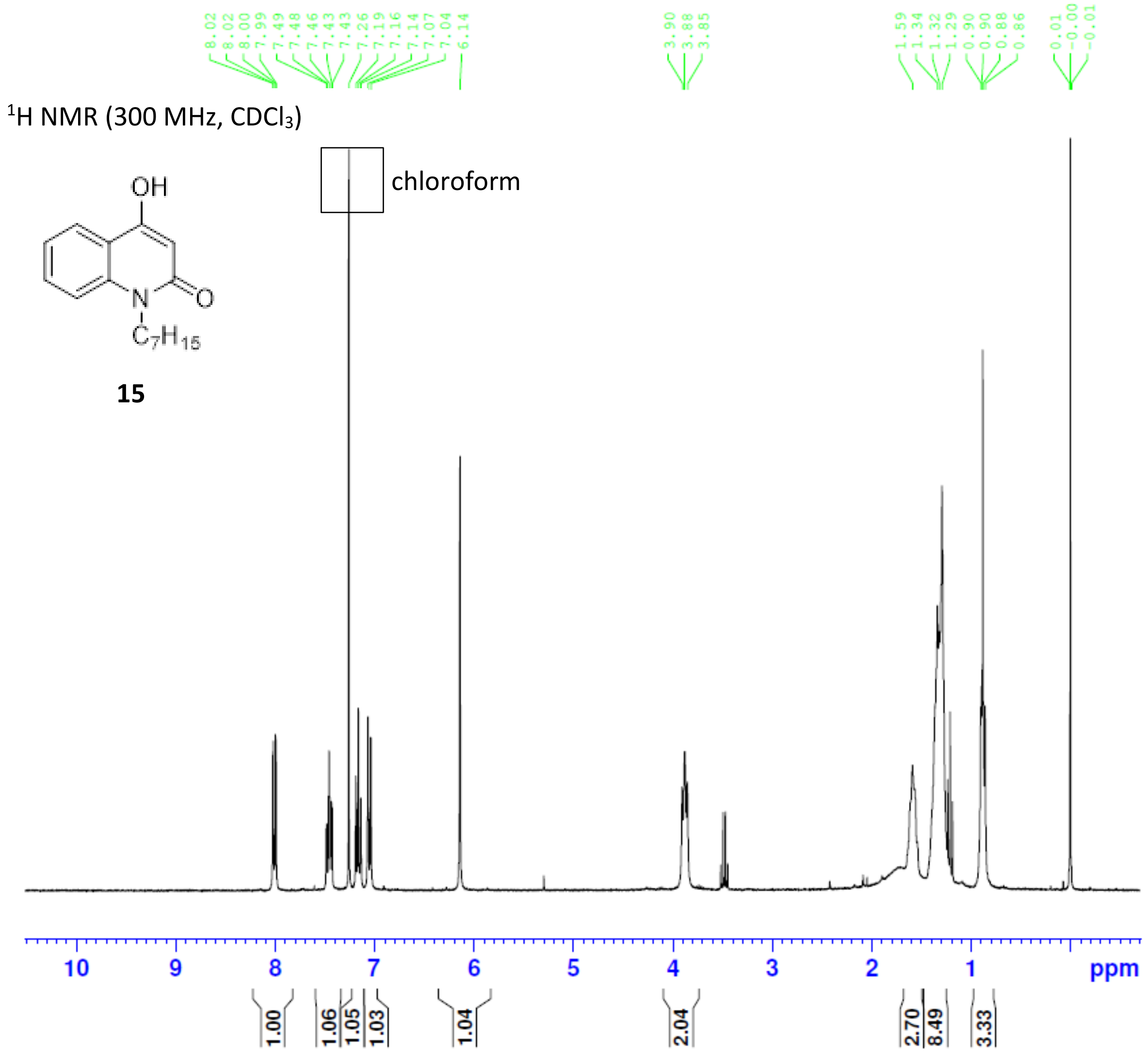

^{1}H NMR (300 MHz, $CDCl_3$)
OH
N
O
C_7H_{15}
15
chloroform
8.02 8.02 8.00 7.99 7.49 7.48 7.46 7.43 7.43 7.26 7.19 7.16 7.14 7.07 7.04 6.14
3.90 3.88 3.85
1.59 1.34 1.32 1.29 0.90 0.90 0.88 0.86
0.01 -0.00 -0.01
10 9 8 7 6 5 4 3 2 1 ppm
1.00 1.06 1.05 1.03 1.04 2.04 2.70 8.49 3.33

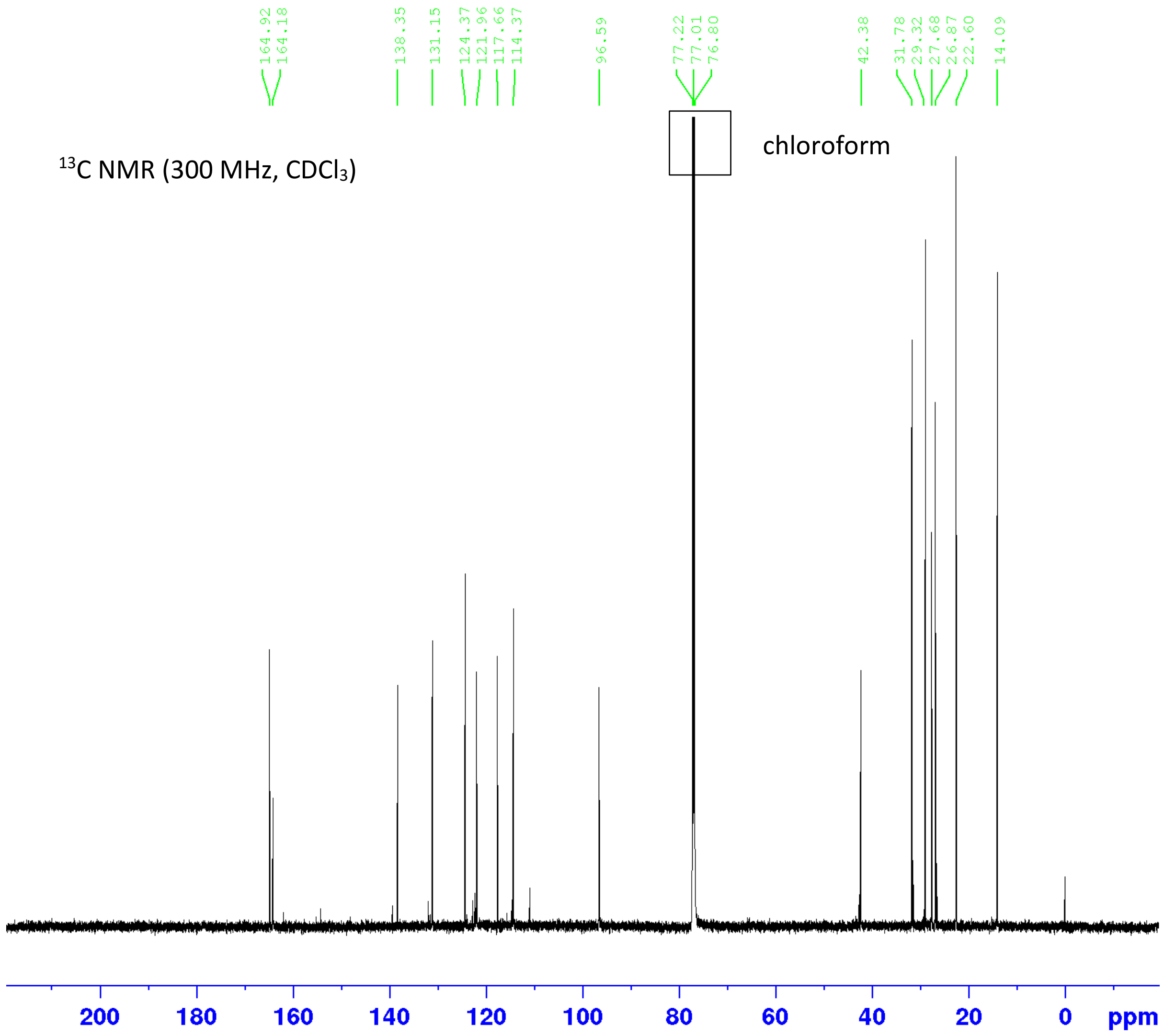

164.92
164.18
138.35
131.15
124.37
121.96
117.66
114.37
96.59
77.22
77.01
76.80
42.38
31.78
29.32
27.68
26.87
22.60
14.09
^{13}C NMR (300 MHz, $CDCl_3$)
chloroform
200
180
160
140
120
100
80
60
40
20
0
ppm

^1H NMR (300 MHz, $CDCl_3$)

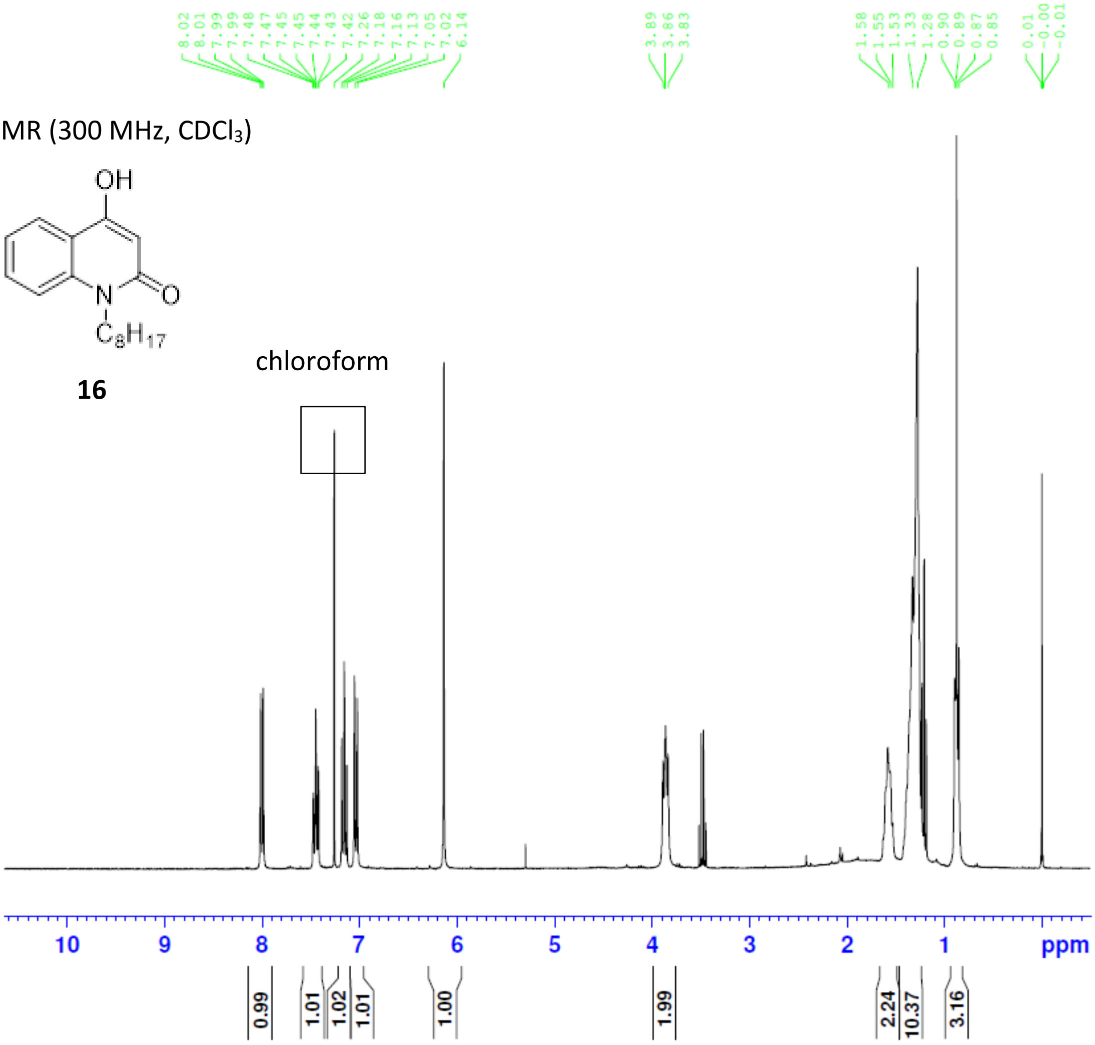

16

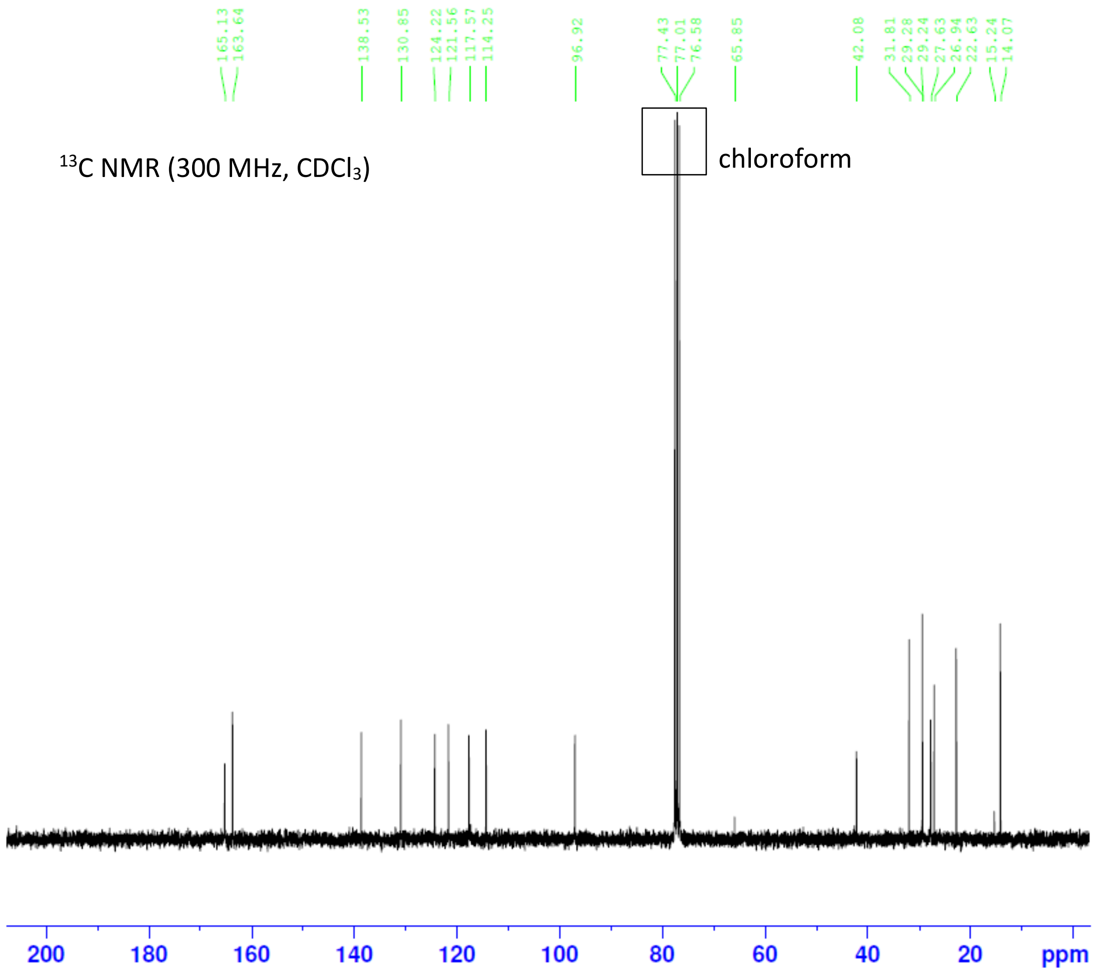

165.13
163.64
138.53
130.85
124.22
121.56
117.57
114.25
96.92
77.43
77.01
76.58
65.85
42.08
31.81
29.28
29.24
27.63
26.94
22.63
15.24
14.07
^{13}C NMR (300 MHz, $CDCl_3$)
chloroform
200
180
160
140
120
100
80
60
40
20
ppm

^{1}H NMR (300 MHz, $CDCl_3$)

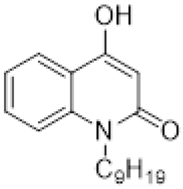

17

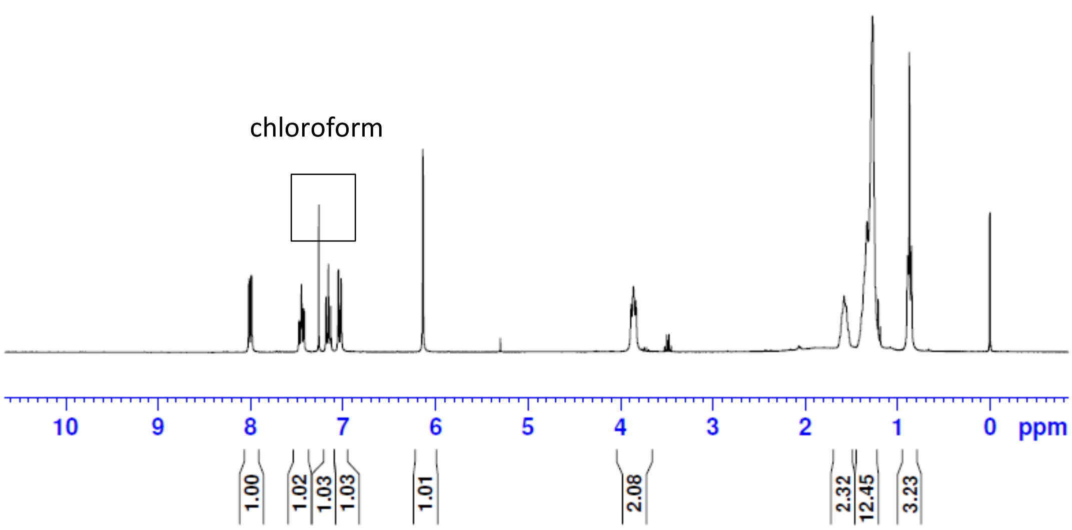

^{13}C NMR (300 MHz, $CDCl_3$)

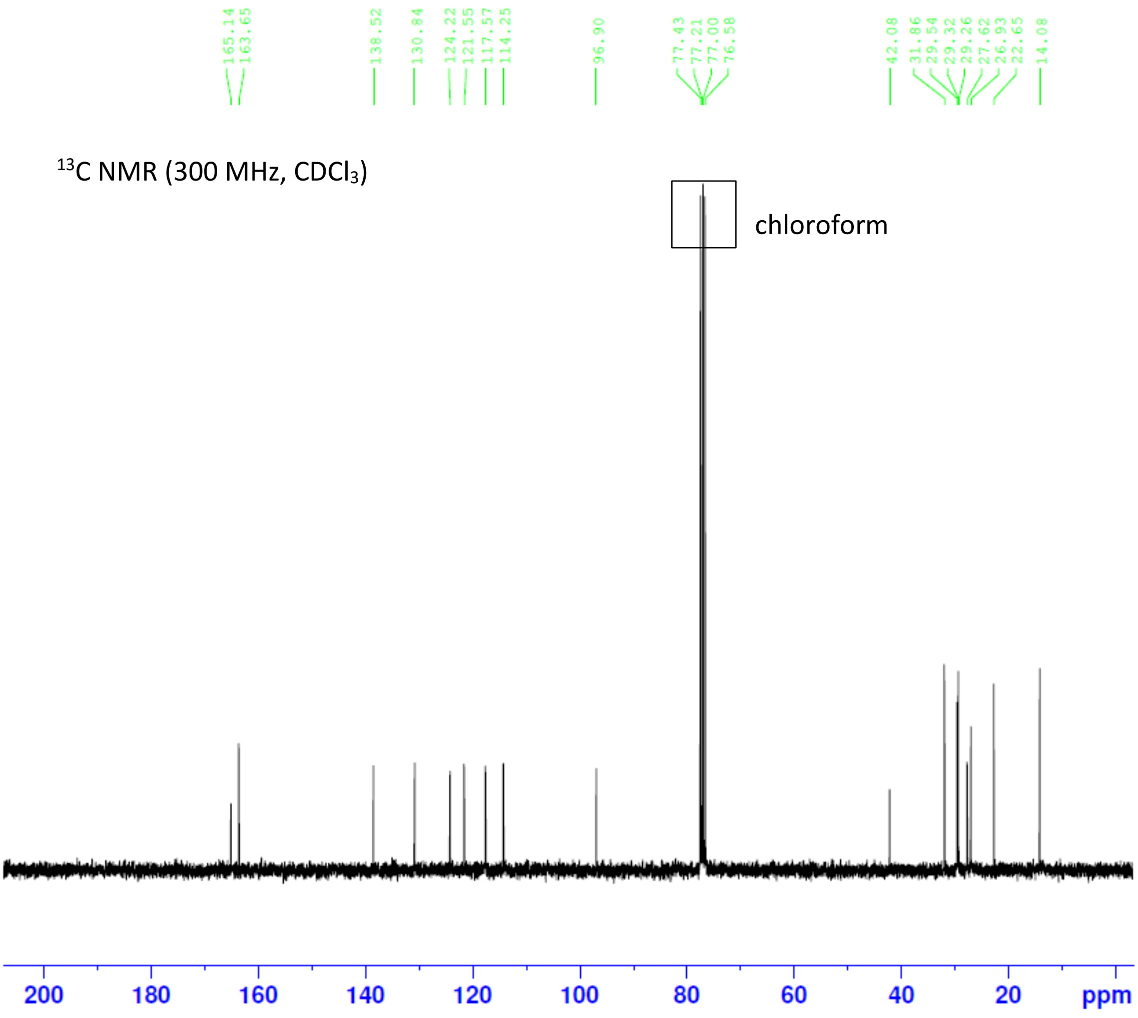

^1H NMR (300 MHz, $CDCl_3$)

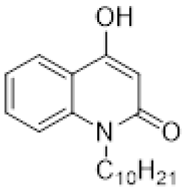

18

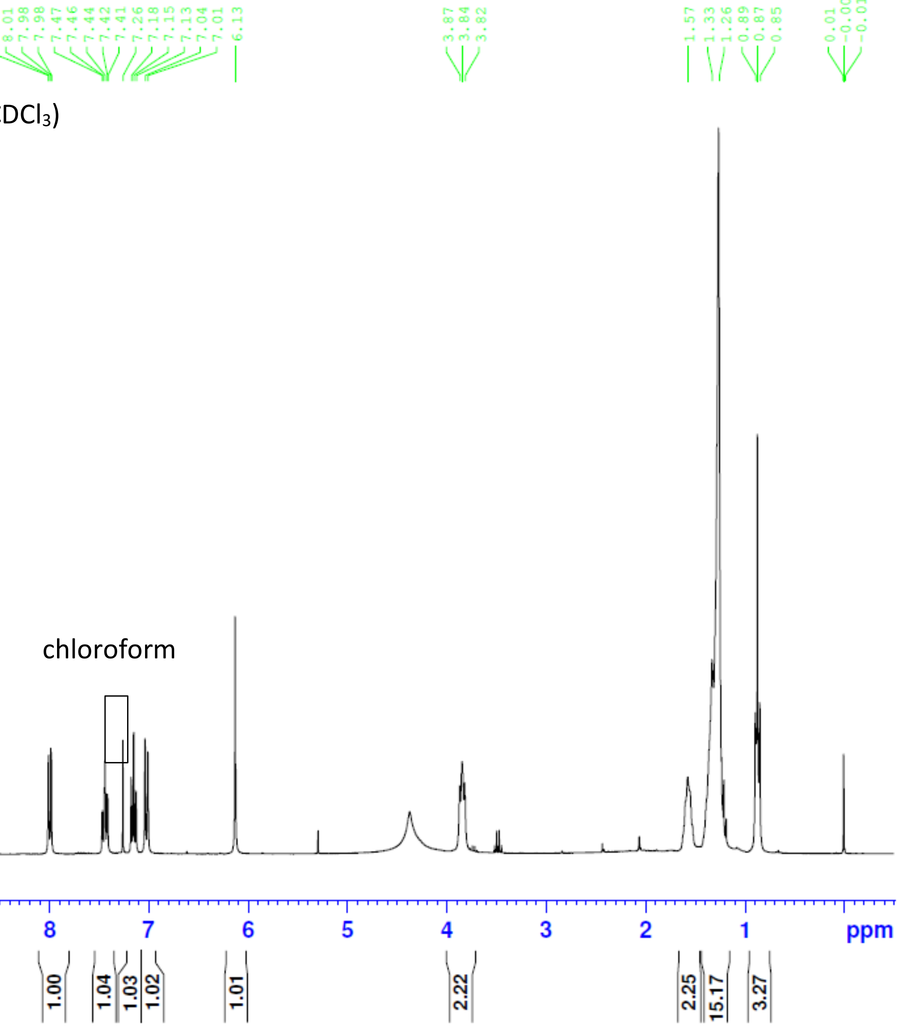

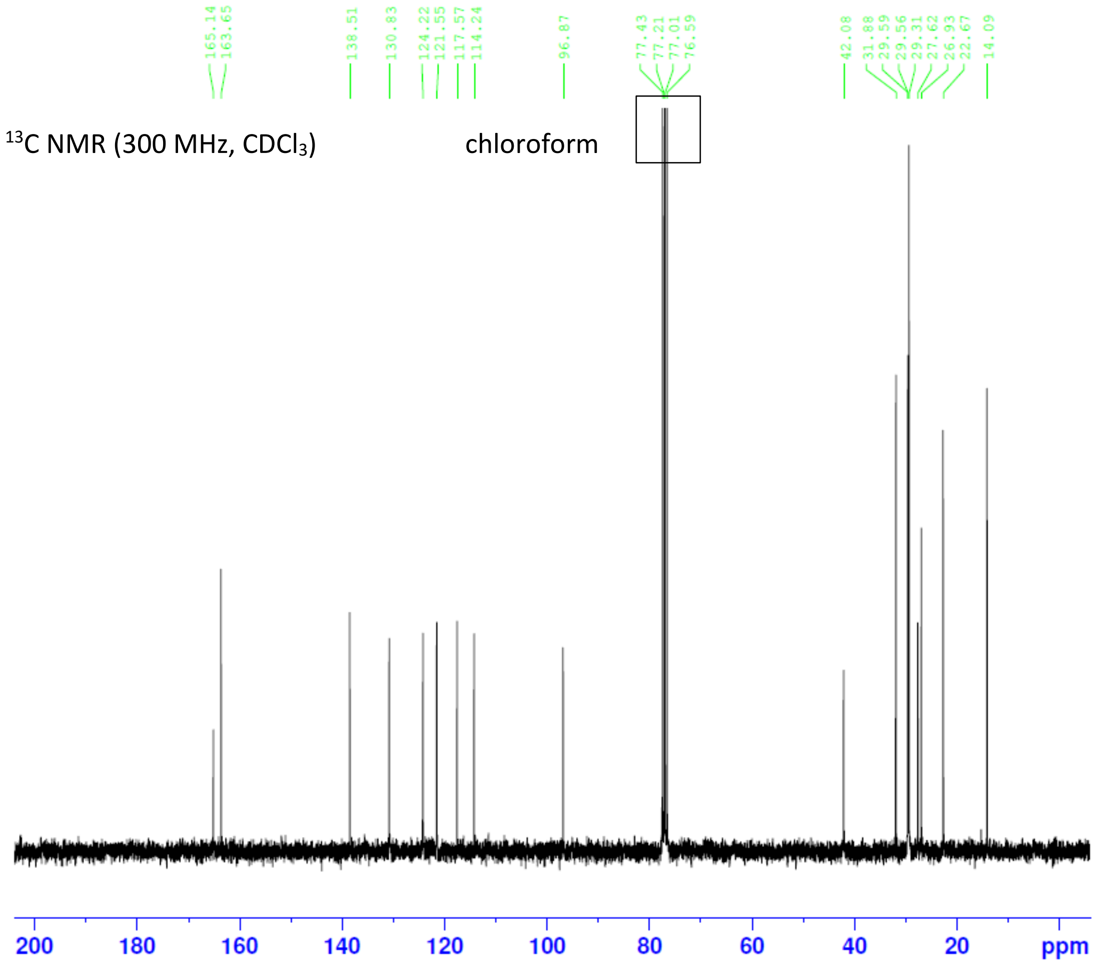

165.14
163.65
138.51
130.83
124.22
121.55
117.57
114.24
96.87
77.43
77.21
77.01
76.59
42.08
31.88
29.59
29.56
29.31
27.62
26.93
22.67
14.09
^{13}C NMR (300 MHz, $CDCl_3$)
chloroform
200
180
160
140
120
100
80
60
40
20
ppm

Supplementary Biological Data

Quinolone antibiotics are known to target the GyrA and ParC proteins in a well established mode of action. Specifically, they disrupt DNA synthesis by inhibiting the catalytic activity of DNA gyrase and topoisomerase IV. Therefore, we investigated the GyrAB and ParCE proteins in the test strains to determine if the susceptible GP_020 (ATCC 43300) MRSA strain had any specific mutational changes relative to the more tolerant strains used in this study. Other strains included in this analysis were ATCC 25923 (GP_001), ATCC 33591 (GP_021), and ATCC 700699 (GP_035).

In all alignments, a green to red shift denotes a hot-spot mutational site as per previous studies[13-14] . Note that only GP_035 shows evidence of quinolone resistance with S84L in GyrA, S80F in ParC, and N139S in ParE.

Alignment of GyrA protein sequence from *S. aureus* test strains

```
GP_020        MAELPQSRINERNITSEMRESFLDYAMSVIVARALPDVRDGLKPVHRRILYGLNEQGMTP 60
GP_001        MAELPQSRINERNITSEMRESFLDYAMSVIVARALPDVRDGLKPVHRRILYGLNEQGMTP 60
GP_021        MAELPQSRINERNITSEMRESFLDYAMSVIVARALPDVRDGLKPVHRRILYGLNEQGMTP 60
GP_035        MAELPQSRINERNITSEMRESFLDYAMSVIVARALPDVRDGLKPVHRRILYGLNEQGMTP 60
              ************************************************************

GP_020        DKSYKKSARIVGDVMGKYHPHGDSSIYEAMVRMAQDFSYRYPLVDGQGNFGSMDGDGAAA 120
GP_001        DKSYKKSARIVGDVMGKYHPHGDSSIYEAMVRMAQDFSYRYPLVDGQGNFGSMDGDGAAA 120
GP_021        DKSYKKSARIVGDVMGKYHPHGDSSIYEAMVRMAQDFSYRYPLVDGQGNFGSMDGDGAAA 120
GP_035        DKSYKKSARIVGDVMGKYHPHGDLSIYEAMVRMAQDFSYRYPLVDGQGNFGSMDGDGAAA 120
              *********************** ************************************

GP_020        MRYTEARMTKITLELLRDINKDTIDFIDNYDGNEREPSVLPARFPNLLANGASGIAVGMA 180
GP_001        MRYTEARMTKITLELLRDINKDTIDFIDNYDGNEREPSVLPARFPNLLANGASGIAVGMA 180
GP_021        MRYTEARMTKITLELLRDINKDTIDFIDNYDGNEREPSVLPARFPNLLANGASGIAVGMA 180
GP_035        MRYTEARMTKITLELLRDINKDTIDFIDNYDGNEREPSVLPARFPNLLANGASGIAVGMA 180
              ************************************************************

GP_020        TNIPPHNLTELINGVLSLSKNPDISIAELMEDIEGPDFPTAGLILGKSGIRRAYETGRGS 240
GP_001        TNIPPHNLTELINGVLSLSKNPDISIAELMEDIEGPDFPTAGLILGKSGIRRAYETGRGS 240
GP_021        TNIPPHNLTELINGVLSLSKNPDISIAELMEDIEGPDFPTAGLILGKSGIRRAYETGRGS 240
GP_035        TNIPPHNLTELINGVLSLSKNPDISIAELMEDIEGPDFPTAGLILGKSGIRRAYETGRGS 240
              ************************************************************

GP_020        IQMRSRAVIEERGGGRQRIVVTEIPFQVNKARMIEKIAELVRDKKIDGITDLRDETSLRT 300
GP_001        IQMRSRAVIEERGGGRQRIVVTEIPFQVNKARMIEKIAELVRDKKIDGITDLRDETSLRT 300
GP_021        IQMRSRAVIEERGGGRQRIVVTEIPFQVNKARMIEKIAELVRDKKIDGITDLRDETSLRT 300
GP_035        IQMRSRAVIEERGGGRQRIVVTEIPFQVNKARMIEKIAELVRDKKIDGITDLRDETSLRT 300
              ************************************************************

GP_020        GVRVVIDVRKDANASVILNNLYKQTPLQTSFGVNMIALVNGRPKLINLKEALVHYLEHQK 360
GP_001        GVRVVIDVRKDANASVILNNLYKQTPLQTSFGVNMIALVNGRPKLINLKEALVHYLEHQK 360
GP_021        GVRVVIDVRKDANASVILNNLYKQTPLQTSFGVNMIALVNGRPKLINLKEALVHYLEHQK 360
GP_035        GVRVVIDVRKDANASVILNNLYKQTPLQTSFGVNMIALVNGRPKLINLKEALVHYLEHQK 360
              ************************************************************

GP_020        TVVRRRTQYNLRKAKDRAHILEGLRIALDHIDEIISTIRESETDKVAMESLQQRFKLSEK 420
GP_001        TVVRRRTQYNLRKAKDRAHILEGLRIALDHIDEIISTIRESETDKVAMESLQQRFKLSEK 420
GP_021        TVVRRRTQYNLRKAKDRAHILEGLRIALDHIDEIISTIRESETDKVAMESLQQRFKLSEK 420
GP_035        TVVRRRTQYNLRKAKDRAHILEGLRIALDHIDEIISTIRESDTDKVAMKSLQQRFKLSEK 420
              *****************************************:******:***********
```

```
GP_020      QAQAILDMRLRRLTGLERDKIEAEYNELLNYISELEAILADEEVLLQLVRDELTEIRDRF 480
GP_001      QAQAILDMRLRRLTGLERDKIEAEYNELLNYISELEAILADEEVLLQLVRDELTEIRDRF 480
GP_021      QAQAILDMRLRRLTGLERDKIEAEYNELLNYISELEAILADEEVLLQLVRDELTEIRDRF 480
GP_035      QAQAILDMRLRRLTGLERDKIEAEYNELLNYISELETILADEEVLLQLVRDELTEIRDRF 480
            ************************************:***********************

GP_020      GDDRRTEIQLGGFEDLEDEDLIPEEQIVITLSHNNYIKRLPVSTYRAQNRGGRGVQGMNT 540
GP_001      GDDRRTEIQLGGFEDLEDEDLIPEEQIVITLSHNNYIKRLPVSTYRAQNRGGRGVQGMNT 540
GP_021      GDDRRTEIQLGGFEDLEDEDLIPEEQIVITLSHNNYIKRLPVSTYRAQNRGGRGVQGMNT 540
GP_035      GDDRRTEIQLGGFEDLEDEDLIPEEQIVITLSHNNYIKRLPVSTYRAQNRGGRGVQGMNT 540
            ************************************************************

GP_020      LEEDFVSQLVTLSTHDHVLFFTNKGRVYKLKGYEVPELSRQSKGIPVVNAIELENDEIIS 600
GP_001      LEEDFVSQLVTLSTHDHVLFFTNKGRVYKLKGYEVPELSRQSKGIPVVNAIELENDEIIS 600
GP_021      LEEDFVSQLVTLSTHDHVLFFTNKGRVYKLKGYEVPELSRQSKGIPVVNAIELENDEIIS 600
GP_035      LEEDFVSQLVTLSTHDHVLFFTNKGRVYKLKGYEVPELSRQSKGIPVVNAIELENDEVIS 600
            *********************************************************:**

GP_020      TMIAVKDLESEDNFLVFATKRGVVKRSALSNFSRINRNGKIAISFREDDELIAVRLTSGQ 660
GP_001      TMIAVKDLESEDNFLVFATKRGVVKRSALSNFSRINRNGKIAISFREDDELIAVRLTSGQ 660
GP_021      TMIAVKDLESEDNFLVFATKRGVVKRSALSNFSRINRNGKIAISFREDDELIAVRLTSGQ 660
GP_035      TMIAVKDLESEDNFLVFATKRGVVKRSALSNFSRINRNGKIAISFREDDELIAVRLTSGQ 660
            ************************************************************

GP_020      EDILIGTSHASLIRFPESTLRPLGRTATGVKGITLREGDEVVGLDVAHANSVDEVLVVTE 720
GP_001      EDILIGTSHASLIRFPESTLRPLGRTATGVKGITLREGDEVVGLDVAHANSVDEVLVVTE 720
GP_021      EDILIGTSHASLIRFPESTLRPLGRTATGVKGITLREGDEVVGLDVAHANSVDEVLVVTE 720
GP_035      EDILIGTSHASLIRFPESTLRPLGRTATGVKGITLREGDEVVGLDVAHANSVDEVLVVTE 720
            ************************************************************

GP_020      NGYGKRTPVNDYRLSNRGGKGIKTATITERNGNVVCITTVTGEEDLMIVTNAGVIIRLDV 780
GP_001      NGYGKRTPVNDYRLSNRGGKGIKTATITERNGNVVCITTVTGEEDLMIVTNAGVIIRLDV 780
GP_021      NGYGKRTPVNDYRLSNRGGKGIKTATITERNGNVVCITTVTGEEDLMIVTNAGVIIRLDV 780
GP_035      NGYGKRTPVNDYRLSNRGGKGIKTATITERNGNVVCITTVTGEEDLMIVTNAGVIIRLDV 780
            ************************************************************

GP_020      ADISQNGRAAQGVRLIRLGDDQFVSTVAKVKEDADEEN---EDEQSTVSEDGTEQQREAV 837
GP_001      ADISQNGRAAQGVRLIRLGDDQFVSTVAKVKEDADEEN---EDEQSTVSEDGTEQQREAV 837
GP_021      ADISQNGRAAQGVRLIRLGDDQFVSTVAKVKEDADEEN---EDEQSTVSEDGTEQQREAV 837
GP_035      ADISQNGRAAQGVRLIRLGDDQFVSTVAKVKEDAEDETNEDEQSTSTVSEDGTEQQREAV 840
            **********************************::*.   *:. ***************

GP_020      VNDETPGNAIHTEVIDSEVNDEDGRIEVRQDFMDRVEEDIQQSSDDDEE      886
GP_001      VNDETPGNAIHTEVIDSEVNDEDGRIEVRQDFMDRVEEDIQQSSDDDEE      886
GP_021      VNDETPGNAIHTEVIDSEVNDEDGRIEVRQDFMDRVEEDIQQSSDDDEE      886
GP_035      VNDETPGNAIHTEVIDSEENDEDGRIEVRQDFMDRVEEDIQQSSDEDEE      889
            ****************** **************************:***
```

Alignment of GyrB protein sequence from *S. aureus* test strains

```
GP_020      MVTALSDVNNTDNYGAGQIQVLEGLEAVRKRPGMYIGSTSERGLHHLVWEIVDNSIDEAL  60
GP_001      MVTALSDVNNTDNYGAGQIQVLEGLEAVRKRPGMYIGSTSERGLHHLVWEIVDNSIDEAL  60
GP_021      MVTALSDVNNTDNYGAGQIQVLEGLEAVRKRPGMYIGSTSERGLHHLVWEIVDNSIDEAL  60
GP_035      MVTALSDVNNTDNYGAGQIQVLEGLEAVRKRPGMYIGSTSERGLHHLVWEIVDNSIDEAL  60
            ************************************************************

GP_020      AGYANQIEVVIEKDNWIKVTDNGRGIPVDIQEKMGRPAVEVILTVLHAGGKFGGGGYKVS  120
GP_001      AGYANQIEVVIEKDNWIKVTDNGRGIPVDIQEKMGRPAVEVILTVLHAGGKFGGGGYKVS  120
GP_021      AGYANQIEVVIEKDNWIKVTDNGRGIPVDIQEKMGRPAVEVILTVLHAGGKFGGGGYKVS  120
GP_035      AGYANKIEVVIEKDNWIKVTDNGRGIPVDIQEKMGRPAVEVILTVLHAGGKFGGGGYKVS  120
            *****:******************************************************

GP_020      GGLHGVGSSVVNALSQDLEVYVHRNETIYHQAYKKGVPQFDLKEVGTTDKTGTVIRFKAD  180
GP_001      GGLHGVGSSVVNALSQDLEVYVHRNETIYHQAYKKGVPQFDLKEVGTTDKTGTVIRFKAD  180
GP_021      GGLHGVGSSVVNALSQDLEVYVHRNETIYHQAYKKGVPQFDLKEVGTTDKTGTVIRFKAD  180
GP_035      GGLHGVGSSVVNALSQDLEVYVHRNETIYHQAYKKGVPQFDLKEVGTTDKTGTVIRFKAD  180
            ************************************************************

GP_020      GEIFTETTVYNYETLQQRIRELAFLNKGIQITLRDERDEENVREDSYHYEGGIKSYVELL  240
GP_001      GEIFTETTVYNYETLQQRIRELAFLNKGIQITLRDERDEENVREDSYHYEGGIKSYVELL  240
GP_021      GEIFTETTVYNYETLQQRIRELAFLNKGIQITLRDERDEENVREDSYHYEGGIKSYVELL  240
GP_035      GEIFTETTVYNYETLQQRIRELAFLNKGIQITLRDERDEENVREDSYHYEGGIKSYVELL  240
            ************************************************************

GP_020      NENKEPIHDEPIYIHQSKDDIEVEIAIQYNSGYATNLLTYANNIHTYEGGTHEDGFKRAL  300
GP_001      NENKEPIHDEPIYIHQSKDDIEVEIAIQYNSGYATNLLTYANNIHTYEGGTHEDGFKRAL  300
GP_021      NENKEPIHDEPIYIHQSKDDIEVEIAIQYNSGYATNLLTYANNIHTYEGGTHEDGFKRAL  300
GP_035      NENKEPIHDEPIYIHQSKDDIEVEIAIQYNSGYATNLLTYANNIHTYEGGTHEDGFKRAL  300
            ************************************************************

GP_020      TRVLNSYGLSSKIMKEDKDRLSGEDTREGMTAIISIKHGDPQFEGQTKTKLGNSEVRQVV  360
GP_001      TRVLNSYGLSSKIMKEDKDRLSGEDTREGMTAIISIKHGDPQFEGQTKTKLGNSEVRQVV  360
GP_021      TRVLNSYGLSSKIMKEDKDRLSGEDTREGMTAIISIKHGDPQFEGQTKTKLGNSEVRQVV  360
GP_035      TRVLNSYGLSSKIMKEEKDRLSGEDTREGMTAIISIKHGDPQFEGQTKTKLGNSEVRQVV  360
            ****************:*******************************************

GP_020      DKLFSEHFERFLYENPQVARTVVEKGIMAARARVAAKKAREVTRRKSALDVASLPGKLAD  420
GP_001      DKLFSEHFERFLYENPQVARTVVEKGIMAARARVAAKKAREVTRRKSALDVASLPGKLAD  420
GP_021      DKLFSEHFERFLYENPQVARTVVEKGIMAARARVAAKKAREVTRRKSALDVASLPGKLAD  420
GP_035      DKLFSEHFERFLYENPQVARTVVEKGIMAARARVAAKKAREVTRRKSALDVASLPGKLAD  420
            ************************************************************

GP_020      CSSKSPEECEIFLVEGDSAGGSTKSGRDSRTQAILPLRGKILNVEKARLDRILNNNEIRQ  480
GP_001      CSSKSPEECEIFLVEGDSAGGSTKSGRDSRTQAILPLRGKILNVEKARLDRILNNNEIRQ  480
GP_021      CSSKSPEECEIFLVEGDSAGGSTKSGRDSRTQAILPLRGKILNVEKARLDRILNNNEIRQ  480
GP_035      CSSKSPEECEIFLVEGDSAGGSTKSGRDSRTQAILPLRGKILNVEKARLDRILNNNEIRQ  480
            ************************************************************

GP_020      MITAFGTGIGGDFDLAKARYHKIVIMTDADVDGAHIRTLLLTFFYRFMRPLIEAGYVYIA  540
GP_001      MITAFGTGIGGDFDLAKARYHKIVIMTDADVDGAHIRTLLLTFFYRFMRPLIEAGYVYIA  540
GP_021      MITAFGTGIGGDFDLAKARYHKIVIMTDADVDGAHIRTLLLTFFYRFMRPLIEAGYVYIA  540
GP_035      MITAFGTGIGGDFDLAKARYHKIVIMTDADVDGAHIRTLLLTFFYRFMRPLIEAGYVYIA  540
            ************************************************************

GP_020      QPPLYKLTQGKQKYYVYNDRELDKLKSELNPTPKWSIARYKGLGEMNADQLWETTMNPEH  600
GP_001      QPPLYKLTQGKQKYYVYNDRELDKLKSELNPTPKWSIARYKGLGEMNADQLWETTMNPEH  600
GP_021      QPPLYKLTQGKQKYYVYNDRELDKLKSELNPTPKWSIARYKGLGEMNADQLWETTMNPEH  600
GP_035      QPPLYKLTQGKQKYYVYNDRELDKLKSELNPTPKWSIARYKGLGEMNADQLWETTMNPEH  600
            ************************************************************

GP_020      RALLQVKLEDAIEADQTFEMLMGDVVENRRQFIEDNAVYANLDF    644
GP_001      RALLQVKLEDAIEADQTFEMLMGDVVENRRQFIEDNAVYANLDF    644
GP_021      RALLQVKLEDAIEADQTFEMLMGDVVENRRQFIEDNAVYANLDF    644
GP_035      RALLQVKLEDAIEADQTFEMLMGDVVENRRQFIEDNAVYANLDF    644
            ********************************************
```

Alignment of ParC protein sequence from *S. aureus* test strains

```
GP_020        MSEIIQDLSLEDVLGDRFGRYSKYIIQERALPDVRDGLKPVQRRILYAMYSSGNTHDKNF  60
GP_001        MSEIIQDLSLEDVLGDRFGRYSKYIIQERALPDVRDGLKPVQRRILYAMYSSGNTHDKNF  60
GP_035        MSEIIQDLSLEDVLGDRFGRYSKYIIQERALPDVRDGLKPVQRRILYAMYSSGNTHDKNF  60
GP_021        MSEIIQDLSLEDVLGDRFGRYSKYIIQERALPDVRDGLKPVQRRILYAMYSSGNTHDKNF  60
              ************************************************************

GP_020        RKSAKTVGDVIGQYHPHGDSSVYEAMVRLSQDWKLRHVLIEMHGNNGSIDNDPPAAMRYT  120
GP_001        RKSAKTVGDVIGQYHPHGDSSVYEAMVRLSQDWKLRHVLIEMHGNNGSIDNDPPAAMRYT  120
GP_035        RKSAKTVGDVIGQYHPHGDFSVYEAMVRLSQDWKLRHVLIEMHGNNGSIDNDPPAAMRYT  120
GP_021        RKSAKTVGDVIGQYHPHGDSSVYEAMVRLSQDWKLRHVLIEMHGNNGSIDNDPPAAMRYT  120
              ******************* ****************************************

GP_020        EAKLSLLAEELLRDINKETVSFISNYDDTTLEPMVLPSRFPNLLVNGSTGISAGYATDIP  180
GP_001        EAKLSLLAEELLRDINKETVSFISNYDDTTLEPMVLPSRFPNLLVNGSTGISAGYATDIP  180
GP_035        EAKLSLLAEELLRDINKETVSFIPNYDDTTLEPMVLPSRFPNLLVNGSTGISAGYATDIP  180
GP_021        EAKLSLLAEELLRDINKETVSFIPNYDDTTLEPMVLPSRFPNLLVNGSTGISAGYATDIP  180
              *********************** ************************************

GP_020        PHNLAEVIQATLKYIDNPDITVNQLMKYIKGPDFPTGGIIQGIDGIKKAYESGKGRIIVR  240
GP_001        PHNLAEVIQATLKYIDNPDITVNQLMKYIKGPDFPTGGIIQGIDGIKKAYESGKGRIIVR  240
GP_035        PHNLAEVIQATLKYIDNPDITVNQLMKYIKGPDFPTGGIIQGIDGIKKAYESGKGRIIVR  240
GP_021        PHNLAEVIQATLKYIDNPDITVNQLMKYIKGPDFPTGGIIQGIDGIKKAYESGKGRIIVR  240
              ************************************************************

GP_020        SKVEEETLRNGRKQLIITEIPYEVNKSSLVKRIDELRADKKVDGIVEVRDETDRTGLRIA  300
GP_001        SKVEEETLRNGRKQLIITEIPYEVNKSSLVKRIDELRADKKVDGIVEVRDETDRTGLRIA  300
GP_035        SKVEEETLRNGRKQLIITEIPYEVNKSSLVKRIDELRADKKVDGIVEVRDETDRTGLRIA  300
GP_021        SKVEEETLRNGRKQLIITEIPYEVNKSSLVKRIDELRADKKVDGIVEVRDETDRTGLRIA  300
              ************************************************************

GP_020        IELKKDVNSESIKNYLYKNSDLQISYNFNMVAISDGRPKLMGIRQIIDSYLNHQIEVVAN  360
GP_001        IELKKDVNSESIKNYLYKNSDLQISYNFNMVAISDGRPKLMGIRQIIDSYLNHQIEVVAN  360
GP_035        IELKKDVNSESIKNYLYKNSDLQISYNFNMVAISDGRPKLMGIRQIIDSYLNHQIEVVAN  360
GP_021        IELKKDVNSESIKNYLYKNSDLQISYNFNMVAISDGRPKLMGIRQIIDSYLNHQIEVVAN  360
              ************************************************************

GP_020        RTKFELDNAEKRMHIVEGLIKALSILDKVIELIRSSKNKRDAKENLIEVYEFTEEQAEAI  420
GP_001        RTKFELDNAEKRMHIVEGLIKALSILDKVIELIRSSKNKRDAKENLIEVYEFTEEQAEAI  420
GP_035        RTKFELDNAEKRMHIVEGLIKALSILDKVIELIRSSKNKRDAKENLIEVFEFTEEQAEAI  420
GP_021        RTKFELDNAEKRMHIVEGLIKALSILDKVIELIRSSKNKRDAKENLIEVYEFTEEQAEAI  420
              *************************************************:**********

GP_020        VMLQLYRLTNTDIVALEGEHKELEALIKQLRHILDNHDALLNVIKEELNEIKKKFKSERL  480
GP_001        VMLQLYRLTNTDIVALEGEHKELEALIKQLRHILDNHDALLNVIKEELNEIKKKFKSERL  480
GP_035        VMLQLYRLTNTDIVALEGEHKELEALIKQLRHILDNHDALLNVIKEELNEIKKKFKSERL  480
GP_021        VMLQLYRLTNTDIVALEGEHKELEALIKQLRHILDNHDALLNVIKEELNEIKKKFKSERL  480
              ************************************************************

GP_020        SLIEAEIEEIKIDKEVMVPSEEVILSMTRHGYIKRTSIRSYNASGVEDIGLKDGDSLLKH  540
GP_001        SLIEAEIEEIKIDKEVMVPSEEVILSMTRHGYIKRTSIRSYNASGVEDIGLKDGDSLLKH  540
GP_035        SLIEAEIEEIKIDKEVMVPSEEVILSMTRHGYIKRTSIRSFNASGVEDIGLKDGDSLLKH  540
GP_021        SLIEAEIEEIKIDKEVMVPSEEVILSMTRHGYIKRTSIRSFNASGVEDIGLKDGDSLLKH  540
              ****************************************:*******************

GP_020        QEVNTQDTVLVFTNKGRYLFIPVHKLADIRWKELGQHVSQIVPIEEDEVVINVFNEKDFN  600
GP_001        QEVNTQDTVLVFTNKGRYLFIPVHKLADIRWKELGQHVSQIVPIEEDEVVINVFNEKDFN  600
GP_035        QEVNTQDTVLVFTNKGRYLFIPVHKLADIRWKELGQHVSQIVPIEEDEVVINVFNEKDFN  600
GP_021        QEVNTQDTVLVFTNKGRYLFIPVHKLADIRWKELGQHVSQIVPIEEDEVVINVFNEKDFN  600
              ************************************************************

GP_020        TDAFYVFATQNGMIKKSTVPLFKTTRFNKPLIATKVENDDLISVMRFEKDQLITIITNK  660
GP_001        TDAFYVFATQNGMIKKSTVPLFKTTRFNKPLIATKVENDDLISVMRFEKDQLITIITNK  660
GP_035        TDAFYVFATQNGMIKKSTVPLFKTTRFNKPLIATKVENDDLISVMRFEKDQLITVITNK  660
GP_021        TDAFYVFATQNGMIKKSTVPLFKTTRFNKPLIATKVENDDLISVMRFEKDQLITVITNK  660
              *******************************************************:****
```

```
GP_020      GMSLTYNTSELSDTGLRAAGVKSINLKAEDFVVMTEGVSENDTILMATQRGSLKRISFKI 720
GP_001      GMSLTYNTSELSDTGLRAAGVKSINLKAEDFVVMTEGVSENDTILMATQRGSLKRISFKI 720
GP_035      GMSLTYNTSELSDTGLRAAGVKSINLKAEDFVVMTEGVSENDTILMATQRGSLKRISFKI 720
GP_021      GMSLTYNTSELSDTGLRAAGVKSINLKAEDFVVMTEGVSENDTILMATQRGSLKRISFKI 720
            ************************************************************

GP_020      LQVAKRAQRGITLLKELKKNPHRIVAAHVVTGEHSQYTLYSKSNEEHGLINDIHKSEQYT 780
GP_001      LQVAKRAQRGITLLKELKKNPHRIVAAHVVTGEHSQYTLYSKSNEEHGLINDIHKSEQYT 780
GP_035      LQVAKRAQRGITLLKELKKNPHRIVAAHVVTGEHSQYTLYSKSNEEHGLINDIHKSEQYT 780
GP_021      LQVAKRAQRGITLLKELKKNPHRIVAAHVVTGEHSQYTLYSKSNEEHGLINDIHKSEQYT 780
            ************************************************************

GP_020      NGSFIVDTDDFGEVIDMYIS 800
GP_001      NGSFIVDTDDFGEVIDMYIS 800
GP_035      NGSFIVDTDDFGEVIDMYIS 800
GP_021      NGSFIVDTDDFGEVIDMYIS 800
            ********************
```

Alignment of ParE protein sequence from *S. aureus* test strains

```
GP_035      MNKQNNYSDDSIQVLEGLEAVRKRPGMYIGSTDKRGLHHLVYEIVDNSVDEVLNGYGNEI  60
GP_021      MNKQNNYSDDSIQVLEGLEAVRKRPGMYIGSTDKRGLHHLVYEIVDNSVDEVLNGYGNEI  60
GP_020      MNKQNNYSDDSIQVLEGLEAVRKRPGMYIGSTDKRGLHHLVYEIVDNSVDEVLNGYGNEI  60
GP_001      MNKQNNYSDDSIQVLEGLEAVRKRPGMYIGSTDKRGLHHLVYEIVDNSVDEVLNGYGNEI  60
            ************************************************************

GP_035      DVTINKDGSISIEDNGRGMPTGIHKSGKPTVEVIFTVLHAGGKFGQGGYKTSGGLHGVGA  120
GP_021      DVTINKDGSISIEDNGRGMPTGIHKSGKPTVEVIFTVLHAGGKFGQGGYKTSGGLHGVGA  120
GP_020      DVTINKDGSISIEDNGRGMPTGIHKSGKPTVEVIFTVLHAGGKFGQGGYKTSGGLHGVGA  120
GP_001      DVTINKDGSISIEDNGRGMPTGIHKSGKPTVEVIFTVLHAGGKFGQGGYKTSGGLHGVGA  120
            ************************************************************

GP_035      SVVNALSEWLEVEIHRDGSIYHQSFKNGGSPSSGLVKKGKTKKTGTKVTFKPDDTIFKAS  180
GP_021      SVVNALSEWLEVEIHRDGNIYHQSFKNGGSPSSGLVKKGKTKKTGTKVTFKPDDTIFKAS  180
GP_020      SVVNALSEWLEVEIHRDGNIYHQSFKNGGSPSSGLVKKGKTKKTGTKVTFKPDDTIFKAS  180
GP_001      SVVNALSEWLEVEIHRDGNIYHQSFKNGGSPSSGLVKKGKTKKTGTKVTFKPDDTIFKAS  180
            ******************.*****************************************

GP_035      TSFNFDVLSERLQESAFLLKNLKITLNDLRSGKERQEHYHYEEGIKEFVSYVNEGKEVLH  240
GP_021      TSFNFDVLSERLQESAFLLKNLKITLNDLRSGKERQEHYHYEEGIKEFVSYVNEGKEVLH  240
GP_020      TSFNFDVLSERLQESAFLLKNLKITLNDLRSGKERQENYHYEEGIKEFVSYVNEGKEVLH  240
GP_001      TSFNFDVLSERLQESAFLLKNLKITLNDLRSGKERQEHYHYEEGIKEFVSYVNEGKEVLH  240
            *************************************:**********************

GP_035      DVATFSGEANGIEVDVAFQYNDQYSESILSFVNNVRTKDGGTHEVGFKTAMTRVFNDYAR  300
GP_021      DVATFSGEANGIEVDVAFQYNDQYSESILSFVNNVRTKDGGTHEVGFKTAMTRVFNDYAR  300
GP_020      DVATFSGEANGIEVDVAFQYNDQYSESILSFVNNVRTKDGGTHEVGFKTAMTRVFNDYAR  300
GP_001      DVATFSGEANGIEVDVAFQYNDQYSESILSFVNNVRTKDGGTHEVGFKTAMTRVFNDYAR  300
            ************************************************************

GP_035      RINELKTKDKNLDGNDIREGLTAVVSVRIPEELLQFEGQTKSKLGTSEARSAVDSVVADK  360
GP_021      RINELKTKDKNLDGNDIREGLTAVVSVRIPEELLQFEGQTKSKLGTSEARSAVDSVVADK  360
GP_020      RINELKTKDKNLDGNDIREGLTAVVSVRIPEELLQFEGQTKSKLGTSEARSAVDSVVADK  360
GP_001      RINELKTKDKNLDGNDIREGLTAVVSVRIPEELLQFEGQTKSKLGTSEARSAVDSVVADK  360
            ************************************************************

GP_035      LPFYLEEKGQLSKSLVKKAIKAQQAREAARKAREDARSGKKNKRKDTLLSGKLTPAQSKN  420
GP_021      LPFYLEEKGQLSKSLVKKAIKAQQAREAARKAREDARSGKKNKRKDTLLSGKLTPAQSKN  420
GP_020      LPFYLEEKGQLSKSLVKKAIKAQQAREAARKAREDARSGKKNKRKDTLLSGKLTPAQSKN  420
GP_001      LPFYLEEKGQLSKSLVKKAIKAQQAREAARKAREDARSGKKNKRKDTLLSGKLTPAQSKN  420
            ************************************************************

GP_035      TEKNELYLVEGDSAGGSAKLGRDRKFQAILPLRGKVINTEKARLEDIFKNEEINTIIHTI  480
GP_021      TEKNELYLVEGDSAGGSAKLGRDRKFQAILPLRGKVINTEKARLEDIFKNEEINTIIHTI  480
GP_020      TDKNELYLVEGDSAGGSAKLGRDRKFQAILPLRGKVINTEKARLEDIFKNEEINTIIHTI  480
GP_001      TDKNELYLVEGDSAGGSAKLGRDRKFQAILPLRGKVINTEKARLEDIFKNEEINTIIHTI  480
            *:**********************************************************

GP_035      GAGVGTDFKIEDSNYNRVIIMTDADTDGAHIQVLLLTFFFKYMKPLVQAGRVFIALPPLY  540
GP_021      GAGVGTDFKIEDSNYNRVIIMTDADTDGAHIQVLLLTFFFKYMKPLVQAGRVFIALPPLY  540
GP_020      GAGVGTDFKIEDSNYNRVIIMTDADTDGAHIQVLLLTFFFKYMKPLVQAGRVFIALPPLY  540
GP_001      GAGVGTDFKIEDSNYNRVIIMTDADTDGAHIQVLLLTFFFKYMKPLVQAGRVFIALPPLY  540
            ************************************************************

GP_035      KLEKGKGKTKRVEYAWTDEELNKLQKELGKGFTLQRYKGLGEMNPEQLWETTMNPETRTL  600
GP_021      KLEKGKGKTKRVEYAWTDEELNKLQKELGKGFTLQRYKGLGEMNPEQLWETTMNPETRTL  600
GP_020      KLEKGKGKTKRVEYAWTDEELNKLQKELGKGFTLQRYKGLGEMNPEQLWETTMNPETRTL  600
GP_001      KLEKGKGKTKRVEYAWTDEELNKLQKELGKGFTLQRYKGLGEMNPEQLWETTMNPETRTL  600
            ************************************************************

GP_035      IRVQVEDEVRSSKRVTTLMGDKVQPRREWIEKHVEFGMQEDQSILDNSEVQVLENDQFDE  660
GP_021      IRVQVEDEVRSSKRVTTLMGDKVQPRREWIEKHVEFGMQEDQSILDNSEVQVLENDQFDE  660
GP_020      IRVQVEDEVRSSKRVTTLMGDKVQPRREWIEKHVEFGMQEDQSILDNSEVQVLENDQFDE  660
GP_001      IRVQVEDEVRSSKRVTTLMGDKVQPRREWIEKHVEFGMQEDQSILDNSEVQVLENDQFDE  660
            ************************************************************
```

```
GP_035      EEI     663
GP_021      EEI     663
GP_020      EEI     663
GP_001      EEI     663
            ***
```

Alignment of Cytochrome d ubiquinol oxidase subunit I protein sequence from *S. aureus* strains used in this study

```
GP021      MDTVEISRFLTAMTLAVHIIFATIGVGMPLMFAIAEFLGIRKNDLQYIAMAKRWAKAYTI   60
GP035      MDTVEISRFLTAMTLAVHIIFATIGVGMPLMFAIAEFLGIRKNDLQYIAMAKRWAKAYTI   60
GP020      MDTVEISRFLTAMTLAVHIIFATIGVGMPLMFAIAEFLGIRKNDLQYIAMAKRWAKAYTI   60
GP001      MDTVEISRFLTAMTLAVHIIFATIGVGMPLMFAIAEFLGIRKNDLQYIAMAKRWAKAYTI   60
           ************************************************************

GP021      TVAVGVVTGTIIGLQLSLIWPTFMEMGGHVIALPLFMETFAFFFEAIFLSIYLYTWDRFK   120
GP035      TVAVGVVTGTIIGLQLSLIWPTFMEMGGHVIALPLFMETFAFFFEAIFLSIYLYTWDRFK   120
GP020      TVAVGVVTGTIIGLQLSLIWPTFMEMGGHVIALPLFMETFAFFFEAIFLSIYLYTWDRFK   120
GP001      TVAVGVVTGTIIGLQLSLIWPTFMEMGGHVIALPLFMETFAFFFEAIFLSIYLYTWDRFK   120
           ************************************************************

GP021      NKWTHFLISIPVIIGGSFSAFFITSVNSFMNTPAGFELKNGKMVNVQPIEAMFNPSFIVR   180
GP035      NKWTHFLISIPVIIGGSFSAFFITSVNSFMNTPAGFELKNGKMVNVQPIEAMFNPSFIVR   180
GP020      NKWTHFLISIPVIIGGSFSAFFITSVNSFMNTPAGFELKNGKMVNVQPIEAMFNPSFIVR   180
GP001      NKWTHFLISIPVIIGGSFSAFFITSVNSFMNTPAGFELKNGKMVNVQPIEAMFNPSFIVR   180
           ************************************************************

GP021      SFHVITTAGMTMAFVIASIAAFKLLRNRQPKDTVYHKKALKMSMIVGFFSTLLSMLAGDL   240
GP035      SFHVITTAGMTMAFVIASIAAFKLLRNRQPKDTVYHKKALKMSMIVGFFSTLLSMLAGDL   240
GP020      SFHVITTAGMTMAFVIASIAAFKLLRNRQPKDTVYHKKALKMSMIVGFFSTLLSMLAGDL   240
GP001      SFHVITTAGMTMAFVIASIAAFKLLRNRQPKDTVYHKKALKMSMIVGFFSTLLSMLAGDL   240
           ************************************************************

GP021      SAKFLHKFQPEKLAAYEWHFDTSSHAKLLLFGVLDEKTQQVKGAIELPGLLSFLADNSVK   300
GP035      SAKFLHKFQPEKLAAYEWHFDTSSHAKLLLFGVLDEKTQQVKGAIELPGLLSFLADNSVK   300
GP020      SAKFLHKFQPEKLAAYEWHFDTSSHAKLLLFGVLDEKTQQVKGAIELPGLLSFLADNSVK   300
GP001      SAKFLHKFQPEKLAAYEWHFDTSSHAKLLLFGMLDEKTQQVKGAIELPGLLSFLADNSVK   300
           ********************************:***************************

GP021      KVQGLNDFPKSLHPPMIVHYFFDLMVTMGILCFVISGVYVLTLMFKKLRKFSTHKWMLY    360
GP035      TKVQGLNDFPKSLHPPMIVHYFFDLMVTMGILCFVISGVYVLTLMFKKLRKFSTHKWMLY   360
GP020      TKVQGLNDFPKSLHPPMIVHYFFDLMVTMGILCFVISGVYVLTLMFKKLRNFSTHKWMLY   360
GP001      TKVQGLNDFPKSLHPPMIVHYFFDLMVTMGILCFVISGVYVLTLMFKKLRNFSTHKWMLY   360
           **************************************************:*********

GP021      GILLTGPASMLAIEFGWFLTEMGRQPWIVRGYMRVAEAATQAGGITFVTILFGILYIILM   420
GP035      GILLTGPASMLAIEFGWFLTEMGRQPWIVRGYMRVAEAATQAGGITFVTILFGILYIILM   420
GP020      GILLTGPASMLAIEFGWFLTEMGRQPWIVRGYMRVAEAATQAGGITFVTILFGILYTILM   420
GP001      GILLTGPASMLAIEFGWFLTEMGRQPWIVRGYMRVAEAATQAGGITFVTILFGILYTILM   420
           ******************************************************** ***

GP021      YTCAYVLIRMFKNKPAYEDVNRLAKKQGGEIEK   453
GP035      YTCAYVLIRMFKNKPAYEDVNRLAKKQGGEIEK   453
GP020      YTCAYVLIRMFKNKPAYEDVNRLAKKQGGEIEK   453
GP001      YTCAYVLIRMFKNKPAYEDVNRLAKKQGGEIEK   453
           *********************************
```

<u>Alignment of Cytochrome d ubiquinol oxidase subunit II protein sequence from *S. aureus* strains used in this study</u>

```
GP001      MIYAFIGITVLWLFLFCYIIIASIDFGAGFFALHSKLTGDEKKINHLISRYLNPVWEVTN     60
GP035      MIYAFIGITVLWLFLFCYIIIASIDFGAGFFALHSKLTGDEKKINHLISRYLNPVWEVTN     60
GP021      MIYAFIGITVLWLFLFCYIIIASIDFGAGFFALHSKLTGDEKKINHLISRYLNPVWEVTN     60
GP020      MIYAFIGITVLWLFLFCYIIIASIDFGAGFFALHSKLTGDEKKINHLISRYLNPVWEVTN     60
           ************************************************************

GP001      VFFVFFFVGFVGFFPESIKYLGTVLLIPGSIALIMISLRNSFYAFENYGQDTKLAWMIMY     120
GP035      VFFVFFFVGFVGFFPESIKYLGTVLLVPGSIALIMISLRNSFYAFENYGQDTKLAWMIMY     120
GP021      VFFVFFFVGFVGFFPESIKYLGTVLLIPGSIALIMISLRNSFYAFENYGQDTKLAWMIMY     120
GP020      VFFVFFFVGFVGFFPESIKYLGTVLLIPGSIALIMISLRNSFYAFENYGQDTKLAWMIMY     120
           **************************:*********************************

GP001      GVSGLLIPASLSTALTITEGGYINVRNNVIDLDWVQLLLSPFAWSVVFLAIISVLYISSG     180
GP035      GVSGLLIPASLSTALTITEGGYINVRNNVIDLDWVQLLLSPFAWSVVFLAIISVLYISSG     180
GP021      GVSGLLIPASLSTALTITEGGYINVRNNVIDLDWVQLLLSPFAWSVVFLAIISVLYISSG     180
GP020      GVSGLLIPASLSTALTITEGGYINVRNNVIDLDWVQLLLSPFAWSVVFLAIISVLYISSG     180
           ************************************************************

GP001      FLTYYAKKANDEPAYNLTRQWHIFLGPPMIIICLFVFLSLRIQNSEHFLFSCF-------     233
GP035      FLTYYAKKANDEPAYNLTRQWHIFLGPPMIIICLFVFLSLRIQNSEHFYSAVFDYWWMFA     240
GP021      FLTYYAKKANDEPAYNLTRQWHIFLGPPMIIICLFVFLSLRIQNSEHFYSAVFDYWWMFG     240
GP020      FLTYYAKKANDEPAYNLTRQWHIFLGPPMIIICLFVFLSLRIQNSEHFYSAVFDYWWMFA     240
           ************************************************  : *

GP001      ------------------------------------------------------------     233
GP035      ISFLFFALASLLTFFKKKHGLAFVFVILQMMFAFFGYGISKLPYLLYPFVKITDAYVNPE     300
GP021      ISFLFFALASLLTFFKKKHGLAFVFVILQMMFAFFGYGISKLPYLLYPFVKITDAYVNPE     300
GP020      ISFLFFALASLLTFFKKKHGLAFVFVILQMMFAFFGYGISKLPYLLYPFVKITDAYVNPE     300


GP001      --------------------------------------- 233
GP035      MGWTLVIVFILGLLLLLPSLILLLRLFVFDKEYVEGKKS 339
GP021      MGWTLVIVFILGLLLLLPSLILLLRLFVFDKEYVEGKKS 339
GP020      MGWTLVIVFILGLLLLLPSLILLLRLFVFDKEYVEGKKS 339
```

Supplementary Biological Methods

Minimum Inhibitory Concentration (MIC) Testing – Part 1

Working solutions and Plate preparation. All test compounds were reported to exhibit poor solubility and therefore all dilutions were prepared in 100% DMSO to achieve maximum solubility. Adequate amounts of working solution, at a highest concentration of 5.12 mg/mL, were prepared for each compound by diluting the stock solution in 100% DMSO in a 96-well non-binding surface (NBS) plate (Corning®Costar®, #3641) (mother plate). The compounds were then serially diluted in 100% DMSO to achieve 8 concentrations ranging between 0.16 – 5.12 mg/mL. Following the addition of inoculum, the resulting DMSO concentration would be 5% in each well. Once dilutions were prepared, 5 μL of each testing concentration from the mother plate was transferred manually into the appropriate wells of 96-well NBS plates (test plates). The assay was conducted in duplicate (n=2). All solutions were thoroughly pipette-mixed during serial dilution in order to maximise carry over of compound. However, this may not have ensured effective transfer of the compounds into subsequent dilutions, thereby reducing the accuracy of expected testing concentrations. The control antibiotic, vancomycin, was serially diluted in water in the mother plate and 10 μL of each concentration was manually transferred, resulting in a concentration range of 1.25 – 40 mg/mL in the test plates. All test plates contained both a negative and positive control. A DMSO control consisting of a final DMSO concentration range (after the addition of inoculum) between 0.3 – 10% was also included to ensure viability of the strains at the final resulting DMSO concentration of 5%.

Bacterial preparation: Three Gram-positive bacterial strains were tested. The strains were cultured on 5% sheep blood agar and incubated overnight at 37°C. Once growth and purity were established, a few bacterial colonies were selected and dissolved in Cation Adjusted Mueller-Hinton Broth (CAMHB, BD™, #212322) and the resulting culture was once again incubated overnight at 37°C. Following this, bacterial strains were diluted 40-fold in CAMHB and incubated for a further 2-3 hours prior to performing the broth microdilution assay.

Table S1. Bacterial strains used in the first round of testing

Strain Number	Organism Name	Strain Description	Special Growth Requirements	Strain ID
**GP_001**	*Staphylococcus aureus*	MSSA	N/A	ATCC 25923
**GP_020**	*Staphylococcus aureus*	MRSA	N/A	ATCC 43300
**GP_021**	*Staphylococcus aureus*	MRSA	N/A	ATCC 33591

MIC broth microdilution assay. Mid-log phase cultures for each strain was diluted in CAMHB and transferred to each well of the compound-containing 96-well plates to give a final cell density of 5 × 10^5 CFU/mL, and a final concentration range of 8 – 256 µg/mL for the 23 test compounds as well as a range of 0.125 – 4 µg/mL for vancomycin. 8 µg/mL was chosen as the lowest tested concentration for the test compounds since previous testing resulted in MICs ranging 16 - >32 µg/mL. The plates were covered and incubated for between 18 – 20 hours at 37°C.

Reads and Analysis. Following incubation, for all strains, optical density was read at 600 nm (OD600) using the Tecan M1000 Pro Spectrophotometer. Analysis was performed using Microsoft Excel. MIC was determined as the lowest concentration at which OD600 demonstrated ≥ 80% growth inhibition compared to growth control.

Quality control and Control compounds. Plates contained both a positive growth control (contained inoculum only) and a negative growth control (CAMHB only). All positive and negative controls were observed to have passed. All three strains also grew at 5% DMSO. MICs for the control antibiotic, vancomycin, were observed to have fallen within the expected range for the three bacterial strains.

Table S2. MICs obtained for control antibiotic

Compound Name	GP_001 (MSSA) MIC (µg/mL)	GP_020 (MRSA) MIC (µg/mL)	GP_021 (MRSA) MIC (µg/mL)
**Vancomycin**	2 2	1 1	2 2

Minimum Inhibitory Concentration (MIC) Testing – Part 2

Working solutions and plate preparations. Adequate amounts of working solution, at a highest concentration of 2.56 mg/mL, were prepared for each compound by diluting the stock solution in 100% DMSO in a 96-well NBS plate (mother plate). The compounds were then serially diluted in 100% DMSO to achieve 12 concentrations ranging between 0.0012 – 2.56 mg/mL. However, there was insufficient amounts of compounds **1, 4, 5** and **6** to prepare a top concentration of 2.56 mg/mL. As such, these compounds were prepared at a concentration of 1.28 mg/mL and diluted in 100% DMSO to give 12 concentrations ranging between 0.0006- 1.28 mg/mL. Following the addition of inoculum, the resulting DMSO concentration would be

5% in each well. The control antibiotics were serially diluted in water in the mother plate and 5 µL of each concentration was manually transferred, resulting in a concentration range of 0.0012-2.56 mg/mL in the test plates. All test plates contained both a positive and negative control. The assay was conducted in duplicate (n=2).

Bacterial preparation. 14 Gram-positive bacterial strains were tested. The strains GP_001, 017, 020, 021, 024, 026, 033, 035, 036 and 064 were cultured on 5% sheep blood agar and incubated overnight at 37°C. Once growth and purity were established, a few bacterial colonies were selected and dissolved in CAMHB and the resulting culture was once again incubated overnight at 37°C (GP_064 was incubated with 6 µg/mL of vancomycin). Following this, bacterial strains were diluted 40-fold in CAMHB and incubated for a further 2-3 hours prior to performing the broth microdilution assay. Streptococcus strains GP_013, 014, 023 and 237 were also cultured on 5% sheep blood agar and incubated overnight at 5% CO_2. Due to its slow growing nature, no overnight cultures were prepared for these strains. Instead, bacterial colonies were dissolved directly from the agar plate in CAMHB to form a 0.5 McFarland solution for the assay (direct colony suspension method as per CLSI guidelines).

Table S3. Bacterial strains used in the second round of testing

Species	Strain Number	Organism Name	Strain Description	Special Growth Requirements	Strain ID
Staphylococcus	GP_001	*Staphylococcus aureus*	MSSA	N/A	ATCC 25923
	GP_017	*Staphylococcus epidermidis*	FDA strain PCI 1200, NRS 231	N/A	ATCC 12228
	GP_020	*Staphylococcus aureus*	MRSA	N/A	ATCC 43300
	GP_021	*Staphylococcus aureus*	MRSA	N/A	ATCC 33591
	GP_033	*Staphylococcus epidermidis*	VISE	N/A	NRS 60
	GP_035	*Staphylococcus aureus*	MRSA, VISA	N/A	ATCC 700699
	GP_036	*Staphylococcus aureus*	MRSA, DapRSA	N/A	Clinical Isolate
	GP_064	*Staphylococcus aureus*	VRSA	Overnights in 6 µg/mL of vancomycin	NARSA, VRS 1
Enterococcus	GP_024	*Enterococcus faecium*	Type Strain	N/A	ATCC 35667
	GP_026	*Enterococcus faecium*	VRE	N/A	ATCC 700221
Streptococcus	GP_013	*Streptococcus pneumoniae*	Type Strain	Culture in 5% CO_2 and 5% horse blood	ATCC 33400
	GP_014	*Streptococcus pyogenes*	Type Strain	Culture in 5% and 5% horse blood	ATCC 12344
	GP_023	*Streptococcus pneumoniae*	MDR	Culture in 5% CO_2 and 5% horse blood	ATCC 700677
	GP_237	*Streptococcus pyogenes*	Macrolide resistant	Culture in 5% CO_2 and 5% horse blood	ATCC BAA-1414

As before, the prepared inoculum for each strain was diluted in CAMHB and transferred to each well of the compound-containing 96-well plates to give a final cell density of 5×10^5 CFU/mL, and a final concentration range of 0.03 – 64 µg/mL for 4 compounds and 0.06 – 128 µg/mL for the remaining 5 compounds as well as the control antibiotics (detailed in section 5.3). The plates were covered and incubated for between 18 – 20 hours at 37°C. The assay for the Streptococcus strains was conducted with CAMHB consisting of 5% horse blood and incubated for between 18 – 20 hours at 37°C in 5% CO_2.

Reads and analysis. Following incubation, for all strains - except GP_024 and GP_026 – optical density was read at 600 nm (OD600) using the Tecan M1000 Spectrophotometer.
Due to the slow growing nature of strains GP_024 and GP_026, Resazurin (final concentration 0.002%) was added to the plates and incubated for between 3 – 4 hours, at which point a clear distinction between negative control (blue in colour) and growth (pink in colour) was observed. Optical density for the two strains were read at 570 nm and 600 nm (OD600-570) using the Tecan M1000 Pro Spectrophotometer. Analysis was performed using Microsoft Excel. For strains GP_024 and GP_026, MIC was determined as the lowest concentration at which OD600-570 demonstrated ≥ 80% growth inhibition compared to growth control. For the rest of the strains, MIC was determined as the lowest concentration at which OD600 demonstrated ≥ 80% growth inhibition compared to growth control.

Quality control and Control compounds. Plates contained both a positive growth control (contained inoculum only) and a negative growth control (CAMHB only). All positive and negative controls were observed to have passed. MICs for the control antibiotics were observed to have fallen within the expected range for the three bacterial strains.

Table S4. Strain specific control antibiotic MICs for *Staphylococcus aureus* strains

Compound Name	GP_001 (MSSA) MIC (µg/mL)		GP_020 (MRSA) MIC (µg/mL)		GP_021 (MRSA) MIC (µg/mL)		GP_035 (MRSA, VISA) MIC (µg/mL)		GP_036 (MRSA, DapRSA) MIC (µg/mL)	
**Vancomycin**	1	2	1	1	2	2	8	8	2	2
**Daptomycin**	2	2	1	1	2	2	16	16	4	2
**Dalbavancin**	≤ 0.06	0.125	≤ 0.06	≤ 0.06	0.125	0.125	1	1	0.125	0.125
**Meropenem**	≤ 0.06	≤ 0.06	1	1	32	32	64	64	8	8
**Gentamicin**	0.25	0.25	64	64	4	2	>128	>128	32	64

Table S5. Strain specific control antibiotic MICs for *Staphylococcus epidermidis* strains

Compound Name	GP_017 (PCI 1200, NRS 231) MIC (μg/mL)		GP_033 (VISE) MIC (μg/mL)	
**Vancomycin**	1	2	4	4
**Daptomycin**	2	2	2	4
**Dalbavancin**	≤ 0.06	≤ 0.06	0.25	0.5
**Meropenem**	≤ 0.06	≤ 0.06	4	4
**Gentamicin**	0.125	0.125	0.125	0.25

Table S6. Strain specific control antibiotic MICs for *Enterococcus* spp.

Compound Name	GP_024 (Type Strain) MIC (μg/mL)		GP_026 (VRE) MIC (μg/mL)	
**Vancomycin**	1	0.5	128	64
**Daptomycin**	16	16	16	16
**Dalbavancin**	0.125	0.125	8	8
**Meropenem**	32	16	>128	>128
**Gentamicin**	32	16	>128	>128

References

1. Lu, Z.; Twieg, R. J.; Huang, S. D., *Tetrahedron Lett.*, **2003,** *44*, 6289-6292.
2. Pintado-Sierra, M.; Rasero-Almansa, A. M.; Corma, A.; Iglesias, M.; Sánchez, F., *J. Catal.*, **2013,** *299*, 137-145.
3. Sun, W.-B.; Zhang, P.-Z.; Jiang, T.; Li, C.-K.; An, L.-T.; Shoberu, A.; Zou, J.-P., *Tetrahedron*, **2016,** *72*, 6477-6483.
4. Martínez-Asencio, A.; Ramón, D. J.; Yus, M., *Tetrahedron*, **2011,** *67*, 3140-3149.
5. Shen, Q.; Ogata, T.; Hartwig, J. F., *J. Am. Chem. Soc.*, **2008,** *130*, 6586-6596.
6. Magro, A. A. N.; Eastham, G. R.; Cole-Hamilton, D. J., *Chem. Commun.*, **2007**, 3154-3156.
7. Weickmann, D.; Frey, W.; Plietker, B., *Chem. Eur. J.*, **2013,** *19*, 2741-2748.
8. Li, T.-T.; Liu, G.-Q.; Wang, Y.-M.; Cui, B.; Sun, H.; Li, Y.-M., *Tetrahedron*, **2015,** *71*, 7003-7009.
9. Zeng, L.; Fu, H.; Qiao, R.; Jiang, Y.; Zhao, Y., *Adv. Synth. Catal.*, **2009,** *351*, 1671-1676.
10. Docampo, M. L.; Pellón, R. F.; Estevez-Braun, A.; Ravelo, A. G., *Eur. J. Org. Chem.*, **2007,** 4111-4115.
11. Ukrainets, I. V.; Mospanova, E. V.; Shishkina, S. V., *Chem. Heterocycl. Cmpd.*, **2012,** *48*, 1200-1203.
12. Afonso, A.; Weinstein, J.; Gentles, M. J. Antiviral quinolinone compounds. US5179107(A), 12th January, 1993.
13. Sanfilippo, C. M.; Hesje, C. K.; Haas, W.; Morris, T. W., *Chemother.*, **2011,** *57*, 363-371.

14 Miklos, F.; Dora, S.; Csercsik, R., *Frontiers Microbiol.*, **2017,** *8*, doi.org/10.3389/fmicb.2017.02261.
